# The receptor-like kinase ARK controls symbiotic balance across land plants

**DOI:** 10.1073/pnas.2318982121

**Published:** 2024-07-16

**Authors:** Mara Sgroi, David Hoey, Karina Medina Jimenez, Sarah L. Bowden, Matthew Hope, Emma J. Wallington, Sebastian Schornack, Armando Bravo, Uta Paszkowski

**Affiliations:** ^a^Crop Science Centre, Department of Plant Sciences, University of Cambridge, Cambridge CB3 0LE, United Kingdom; ^b^Sainsbury Laboratory, University of Cambridge, Cambridge CB2 1LR, United Kingdom; ^c^Donald Danforth Plant Science Center, St. Louis, MO 63132; ^d^National Institute of Agricultural Botany, Cambridge CB3 0LE, United Kingdom

**Keywords:** arbuscular mycorrhizal symbiosis, phylogenetics, bryophyte, signaling, receptor-like kinases

## Abstract

The mutualistic arbuscular mycorrhizal (AM) symbiosis arose in land plants more than 450 million years ago and is still widely found in all major land plant lineages. Despite its broad taxonomic distribution, little is known about the molecular components underpinning symbiosis outside of flowering plants. The ARBUSCULAR RECEPTOR-LIKE KINASE (ARK) is required for sustaining AM symbiosis in distantly related angiosperms. Here, we demonstrate that ARK has an equivalent role in symbiosis maintenance in the bryophyte *Marchantia paleacea* and is part of a broad AM genetic program conserved among land plants. In addition, our comparative transcriptome analysis identified evolutionarily conserved expression patterns for several genes in the core symbiotic program required for presymbiotic signaling, intracellular colonization, and nutrient exchange. This study provides insights into the molecular pathways that consistently associate with AM symbiosis across land plants and identifies an ancestral role for *ARK* in governing symbiotic balance.

## Significance

The arbuscular mycorrhizal (AM) symbiosis is considered one of the key adaptations that enabled colonization of land by plants. Here, we identified evolutionarily conserved genes, molecular pathways, and transcription patterns whose association with AM symbiosis arose before the diversification of land plants. Among these, the *ARBUSCULAR RECEPTOR-LIKE KINASE* (*ARK*) plays a crucial role in maintaining mutualism in AM symbiosis. We demonstrate functional conservation of ARK from angiosperms to the liverwort *Marchantia paleacea,* which confirms that its requirement for regulation of symbiosis sustenance evolved before the most recent common ancestor of all land plants.

Plants colonized land between 515 and 470 million years ago (mya) (1). A growing body of evidence suggests that plant–fungal symbioses were a central component of this evolutionary transition (2–9). The favored candidate for this supporting act is arbuscular mycorrhizal (AM) symbiosis, as its monophyletic origin dates back to the most recent common ancestor (MRCA) of tracheophytes and bryophytes (6, 7, 9–13). AM symbiosis is today the most prevalent form of plant–fungal symbiosis on Earth, occurring across all major land plant clades (14).

The association with the fungus improves plant mineral nutrition, in particular phosphate, in exchange for organic carbon (15–23). The primary sites of symbiotic nutrient exchange are arbuscules, highly branched tree-like hyphal structures which develop within plant cells. Each arbuscule is surrounded by a plant-derived periarbuscular membrane (PAM), which creates an extensive surface area for nutrient and signal exchange, thereby contributing to plant and fungal fitness. Plant mutants defective in either the uptake of phosphate or the delivery of organic carbon to the fungus result in shriveled arbuscules and in decreased overall root colonization, suggesting that malnourishment of either symbiont is linked to arbuscule development (24–29). Fungal fitness however also relies on the serine/threonine ARBUSCULAR RECEPTOR-LIKE KINASE 1 (ARK1), which is necessary for maintaining fungal vigor and thus symbiotic balance post arbuscule development (30–32). *ARK* is only found in the genomes of AM-competent plants (21, 30) suggesting a conserved role in symbiosis across land plants.

Our existing knowledge of the molecular mechanisms governing AM symbiosis predominantly relies on evidence obtained from a limited number of angiosperm models, while the molecular characterization of bryophytes is at its dawn (13, 33–36). To address the knowledge gap, we focused on examining the extent to which ARK’s regulatory role is conserved among terrestrial plants. We conducted a comprehensive investigation involving phylogenetic analysis, comparative transcriptomics, and gene coelimination analysis, using the liverwort *Marchantia paleacea* as a bryophyte model. We identified a core cluster of 56 AM symbiosis genes conserved in flowering plants and extant bryophytes. The majority of these genes were transcriptionally up-regulated during symbiosis development in *M. paleacea* and in the angiosperm *Medicago truncatula*, suggesting their ancestral recruitment for AM symbiosis prior to the MRCA of land plants. The overlay of gene-trait coelimination and transcriptomic data spotlighted *ARK* as one of the ancestral AM symbiosis genes, and its regulatory role in controlling symbiotic balance was indeed confirmed to be evolutionarily conserved across land plants.

## Results

### Time-Resolved Transcriptional Responses of *M. paleacea* to AM Symbiosis.

To understand the molecular changes associated with the development of AM symbiosis in bryophytes, we characterized the transcriptome of the liverwort *M. paleacea* at 5, 8, and 11 wk post-inoculation (WPI) with *Rhizophagus irregularis*. *R. irregularis* was previously described to colonize the central part of the thallus parenchyma, which will be referred to as the midrib region (22, 34).

**Fig. 1.**
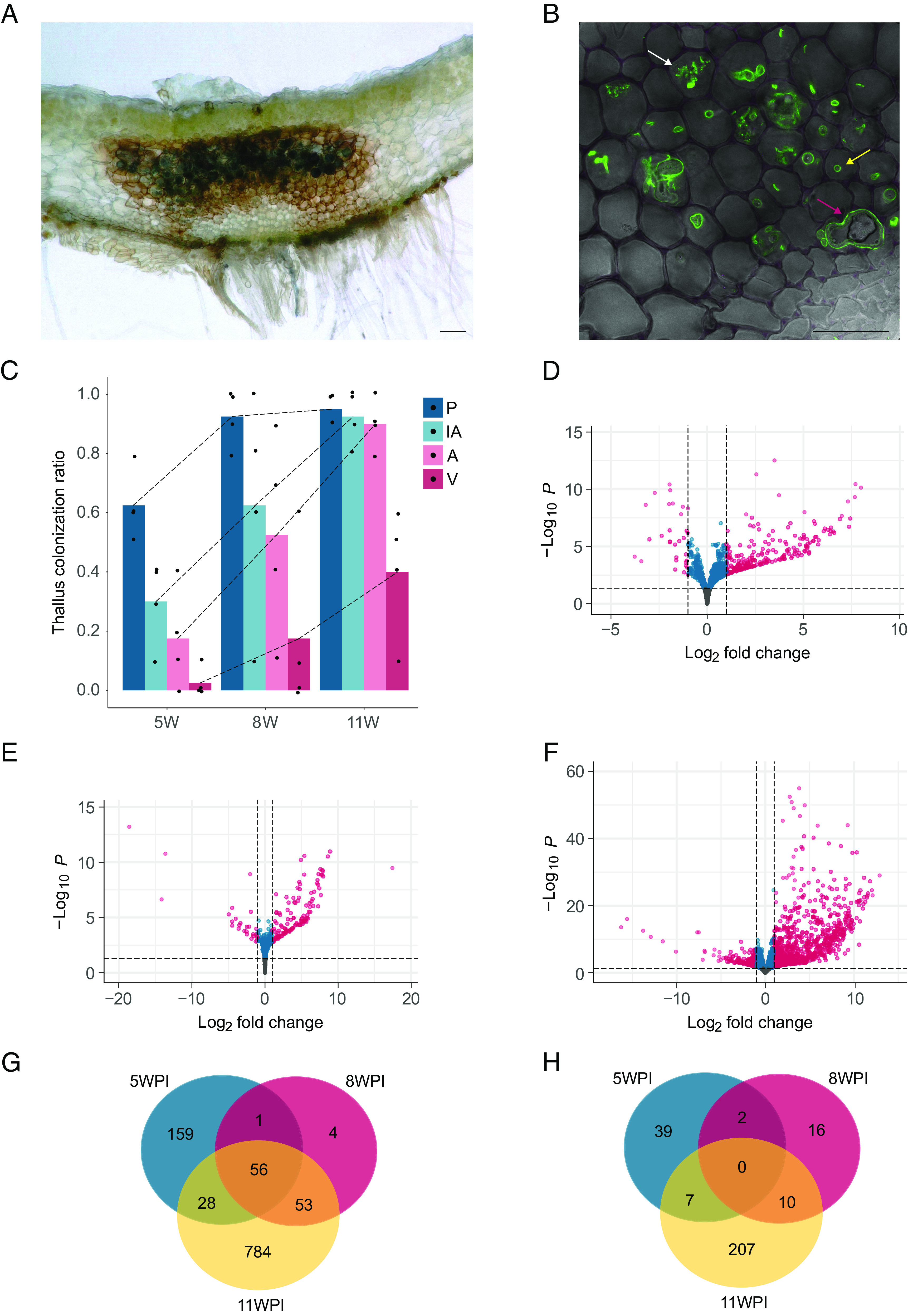
Transcriptional responses of *M. paleacea* to *R. irregularis* colonization. (*A*) Transversal section of a *M. paleacea* thallus colonized by *R. irregularis*; fungal structures in the thallus midrib are stained in ink and recognizable by their dark blue coloration. (Scale bar, 100 μm.) (*B*) Transversal section of an *M. paleacea* thallus colonized by *R. irregularis* and stained with wheat germ agglutinin (WGA)-Alexa Fluor 488; yellow arrow = fungal hypha, white arrow = arbuscule, magenta arrow = fungal vesicle. (Scale bar, 50 μm.) (*C*) Arbuscular mycorrhiza colonization levels of WT *M. paleacea* thalli inoculated with *R. irregularis* at 5, 8, and 11 WPI; P = pigment, IH = intracellular hyphae, A = arbuscules, V = vesicles; each independent replicate was screened for all fungal structures over a total of 10 sections, the overall ratio of positive observation over the total number of observations is displayed on the *y* axis. Bars represent the average number of observations of each fungal structure across replicates, all biological replicates are shown. (*D*) Volcano plot of differentially expressed (DE) genes (adjusted *P*-value < 0.05, Log2 fold change > |1|) between mock and *R. irregularis-*colonized thalli at 5 WPI. Statistically significant DE genes are displayed in magenta. (*E*) Volcano plot of DE genes between mock and *R. irregularis-*colonized thalli at 8 WPI. (*F*) Volcano plot of DE genes between mock and *R. irregularis-*colonized thalli at 11 WPI. (*G*) Venn diagram of up-regulated genes in pairwise comparisons of mock and *R. irregularis-*colonized thalli. (*H*) Venn diagram of down-regulated genes in pairwise comparisons of mock and *R. irregularis-*colonized thalli.

During the initial phases of symbiosis (5 WPI), the primary fungal structures observed within the inoculated thalli were intracellular hyphae (Fig. 1 *A*–*C*). As time progressed, all other fungal structures increased in abundance. For instance, arbuscules increased from an average 20% thallus length colonization at 5 WPI to an average 90% at 11 WPI (Fig. 1*C*). The distinctive purple pigmentation specific to colonized thallus tissue (22) accumulated proportionally to colonization levels across all time points (Fig. 1*C*).

Similarly, the number of DE genes increased over time in response to progressing AM fungal colonization. This represented a fourfold increase between 5 and 11 WPI, from 292 to a total of 1,145 DE genes, with a distinctive bias towards up-regulated genes (80.4%) (Fig. 1 *D*–*H*). The overrepresentation of up-regulated genes mirrors similar observations in distantly related angiosperm models (37–39).

To investigate the identity of DE genes, the transcriptome of *M. paleacea* was functionally annotated. Gene ontology (GO) enrichment analysis identified terms significantly overrepresented (adjusted *P*-value > 0.05) across all stages of AM symbiosis: “gibberellin biosynthetic process”, “gibbrellin-20-oxidase activity”, “oxidoreductase activity”, “nutrient reservoir activity”, “apoplast”, “iron ion binding” (Dataset S2). The strongest transcriptional response to symbiosis at 11 WPI was additionally enriched in terms associated with fatty acid and carbohydrate metabolism and with strigolactone biosynthesis (*SI Appendix,* Fig. S1 and Dataset S2). Overall, GO enrichment analysis highlighted that hormonal signaling pathways and symbiotic nutrient metabolism–related processes, which are commonly associated with angiosperm symbiosis, are also conserved in bryophyte AM symbiosis.

### Identification of Evolutionarily Conserved AM Symbiosis-Associated Genes through Phylogenetics.

We traced the evolutionary history of 104 gene families associated with AM symbiosis in angiosperms to compare expression patterns of symbiosis-regulated genes between *M. paleacea* and tracheophytes (*SI Appendix*, Table S4 and Dataset S3). Each gene family was selected based on at least one of three criteria: a) GO enrichment analysis indicated that the biological process they contribute to is induced in mycorrhizal *M. paleacea*, b) the evolutionary history of the clade suggests that the genes are coeliminated with the loss of AM symbiosis across multiple independent events (21, 40, 41), c) experimental evidence supports a crucial role for the gene in the regulation of AM symbiosis (42, 43). For this analysis, 59 publicly available genomes were selected from all major land plant clades, including all nonflowering plant genomes and a selection of mycorrhizal host and nonhost flowering plant models (*SI Appendix*, Table S5).

**Fig. 2.**
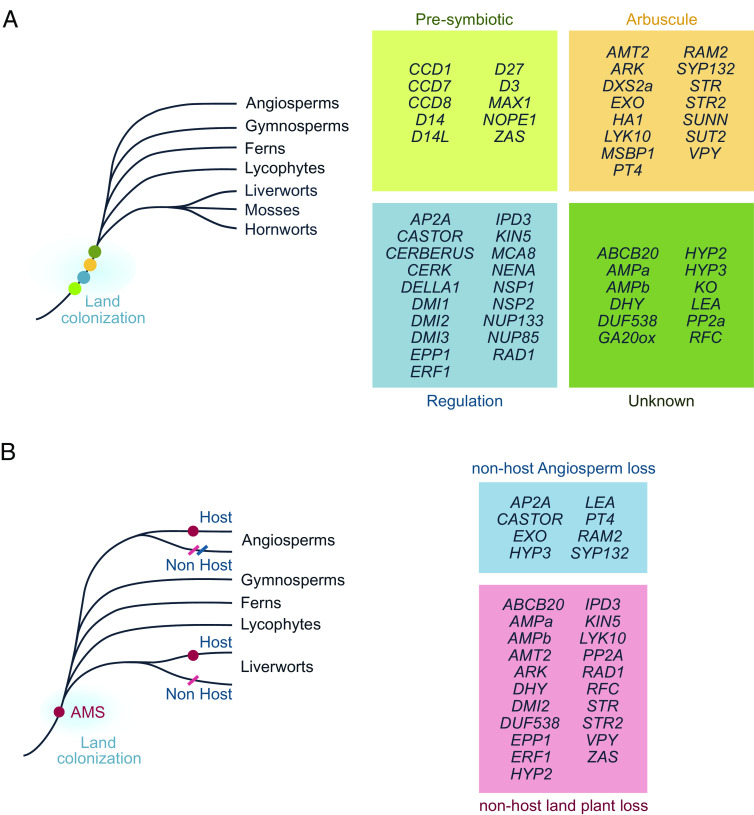
Ancestral AM symbiosis gene families identified through phylogenetics. (*A*) The core conserved AM symbiosis toolkit evolved before the last common ancestor of land plants; evolutionarily conserved genes encompass the following functional groups: presymbiotic signaling, fungal perception and transcriptional regulation, arbuscule-associated genes, and genes of yet unknown function. (*B*) A set of symbiotic genes is conserved across all land plants that are able to engage in AM symbiosis but is recursively coeliminated from the genome of land plants that do not engage in AM symbiosis (pink box); a small set of symbiosis genes is coeliminated with symbiosis abandonment across angiosperms but not in the genus *Marchantia* (light blue box); magenta dot = AM trait, transverse dashes = independent AM trait/gene losses. The phylogenetic trees for each gene family can be found in Dataset S3, and comprehensive information on the results of phylogenetic analysis is available (*SI Appendix*, Table S4). Only genes with clear orthologs in the genus *Marchantia* have been included.

Among the 104 families surveyed, 56 displayed distinct orthologs in *M. paleacea* (Fig. 2*A*, *SI Appendix*, Table S4). The majority (49/56) of the evolutionarily conserved gene families (ECGFs) included a one-to-one ortholog in *M. paleacea,* while in 16/56 ECGFs a single *M. paleacea* “proto-ortholog” corresponded to several angiosperm genes, as a result of one or multiple independent duplications within the tracheophyte lineage (*SI Appendix*, Table S4). These 56 orthogroups span a variety of biological processes required for AM symbiosis: *NOPE1* is necessary for presymbiotic rhizosphere signaling, *RAD1*, *AP2A*, *DELLA1*, *NSP1*, and *NSP2* are involved in transcriptional regulation of the symbiosis, and *AMT2*, *PT4*, *HA1*, *STR*, *STR2*, and *SUT2* are required for nutrient exchange at the arbuscule-interface (Fig. 2*A* and *SI Appendix*, Table S4). Notably, the *SUT2* gene was absent from the latest version of the *M. paleacea* genome but was included in the 1KP transcriptome (Dataset S3). Of the remaining 48 families examined, no clear ortholog was identified in *M. paleacea* or its sister clade *Marchantia polymorpha.* However, in many cases, orthologs were identified in other bryophytes, suggesting that the gene was lost in the genus *Marchantia* (*SI Appendix*, Table S4).

To discern whether ECGFs acquired an AM symbiotic function before the MRCA of land plants, analysis of gene coelimination patterns across multiple independent evolutionary losses was performed. The availability of two liverwort genomes from the same genus with *M. polymorpha* having lost the ability to engage with AM fungi (36, 44), allowed us to extend the study of AM gene coelimination to include bryophytes alongside Angiosperms. We identified 30 gene families only conserved in AM-competent angiosperms (21) and in *M. paleacea* (Fig. 2*B*). Among these gene families, fourteen have experienced the loss of the corresponding *M. polymorpha* gene, indicating that these genes tend to be abandoned when symbiosis is lost in plant species. (Fig. 2*B* and *SI Appendix*, Table S4). For the remaining half of the genes conserved for symbiosis in angiosperms, an ortholog was identified in both the host *M. paleacea* and the nonhost *M. polymorpha*, suggesting that the pattern of coelimination was not conserved for these families (Fig. 2*B*). Overall, we found an unprecedented number of AM genes whose conservation dates back before the MRCA of land plants. The recursive coelimination of a subset of these genes with the loss of AM symbiosis suggests that the conserved genes acquired their symbiotic function in ancestral land plants.

### Comparative Analysis Reveals Deep Homologies in Transcriptional Responses to AM Symbiosis from Bryophytes to Angiosperms.

**Fig. 3.**
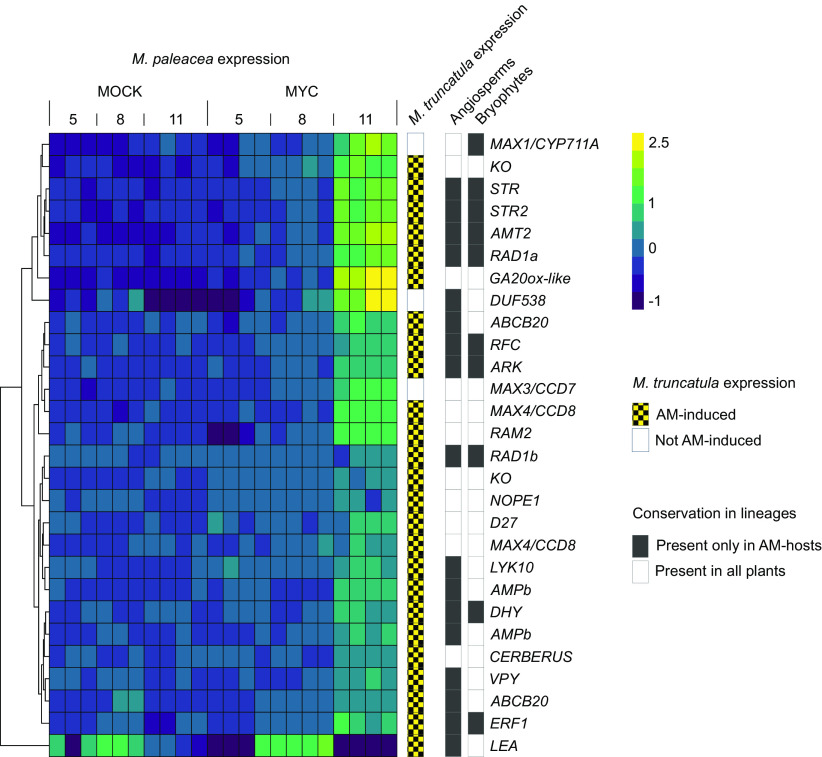
Comparative analysis reveals conserved transcriptional responses to AM symbiosis. Heatmap of variance-stabilized row-centered counts for AM-associated *M. paleacea* evolutionarily conserved genes that were DE during AM symbiosis. *M. truncatula* orthologs DE during AM symbiosis as described previously (23); orthologs significantly DE in both species (L2FC > |1|, adjusted *P*-value < 0.05) are shown in yellow (striped), orthologs not DE in *M. truncatula* are shown in blue (striped); “angiosperm AM” = the gene clade is conserved across all Angiosperms (white) or it is recursively lost after symbiosis abandonment in Angiosperms (gray) (21). “bryophyte AM*”* = The gene is conserved in the host liverwort *M. paleacea* and in the nonhost *M. polymorpha* (white) or lost in the nonhost liverwort *M. polymorpha* (gray).

The symbiotic expression profile of the 56 ECGFs was compared between *M. paleacea* and the angiosperm AM symbiosis model *M. truncatula* (23). Half (28/56) of the genes conserved in *M. paleacea* were DE during symbiosis (Fig. 3 and *SI Appendix*, Table S6). In addition, 89.3% (25/28) of the evolutionarily conserved genes induced in *M. paleacea,* were also up-regulated in *M. truncatula* during symbiosis, demonstrating their conserved transcriptional regulation across land plants (Fig. 3). Several genes with a known role in presymbiotic signaling are co-up-regulated in *M. paleacea* and *M. truncatula*, notably the N-acetylglucosamine transporter *NOPE1* (45) and several members of the strigolactone biosynthesis pathway (Fig. 3). However, the majority of genes coregulated in *M. paleacea* and *M. truncatula* have been linked to downstream stages of symbiosis: transcriptional regulation, arbuscule dynamics, and nutrient exchange, including the well-characterized transcription factors *Required for Arbuscule Development 1* (*RAD1*) and *Ethylene Response Factor 1* (*ERF1*) (Fig. 3 and *SI Appendix*, Table S6). Taken together, the above findings suggest that molecular modules necessary for symbiosis establishment and maintenance in angiosperms are conserved and induced during liverwort AM symbiosis.

Active gibberellin compounds are not known to be produced in bryophytes (44, 46–48). However, gibberellin biosynthetic enzymes were identified and significantly up-regulated in both *M. paleacea* and *M. truncatula,* such as orthologs of *Gibberellin-20-oxidases* (GA20ox) and *ent-Kaurene Oxidase* (*KO*) (Fig. 3 and *SI Appendix*, Table S6). Genes involved in earlier stages of the canonical GA biosynthesis pathway—*ent-Copalyl Diphosphate Synthase* (*MpaCPS*) and *ent-Kaurene Synthase* (*MpaKS*)—as well as the *ent-Kaurenoic Acid Oxidase* (*KAO*) were expressed but not induced (Dataset S1). Thus, the synthesis of Gibberellin-like diterpenoids might take place during bryophyte AM symbiosis.

Despite the overall conservation of transcriptional responses, we identified some differences between *M. paleacea* and *M. truncatula* AM gene expression profiles. In angiosperms, the H^+^-ATPase HA1 is responsible for generating the proton gradient required for phosphate transport across the PAM (49, 50). Although *HA1* is conserved in bryophytes (*Marpal_utg000008g0017601*), it is not induced during symbiosis in *M. paleacea* (*SI Appendix*, Table S6). Instead, three HA proton pumps more distantly related to angiosperm *HA1* were induced in *M. paleacea* (*Marpal_utg000039g0074811*, *Marpal_utg000045g0082001*, and *Marpal_utg000126g0168721*) (Dataset S3). The conservation of a cellular function required for AM symbiosis might thus not be necessarily mediated by conservation of a specific gene.

By overlaying the comparative transcriptomics dataset with the coelimination analysis across angiosperms and liverworts, we identified a core set of ten AM-specific gene families: *ARK*, *RAD1, ERF1, Replication factor C* (*RFC)*, *Ammonium Transporter 2* (*AMT2)*, short-chain Dehydrogenase/reductase (*DHY)*, *Stunted Arbuscule* (*STR*) *and Stunted Arbuscule 2* (*STR2*)*, ABC transporter B family member 20a* (*ABCB20a*), and *ABC transporter B family member 20b* (*ABCB20b*) (Fig. 3). The transcriptional induction of a representative subset of these gene families during AM symbiosis was validated in an independent experiment and confirmed (*SI Appendix*, Fig. S3). In summary, the embryophyte-wide transcriptional induction during symbiosis and the coelimination of these genes with the loss of AM symbiosis provide independent lines of evidence for their mycorrhizal role before the MRCA of land plants.

### The Receptor-Like Kinase ARK Is Required for Symbiosis Maintenance in Bryophytes.

**Fig. 4.**
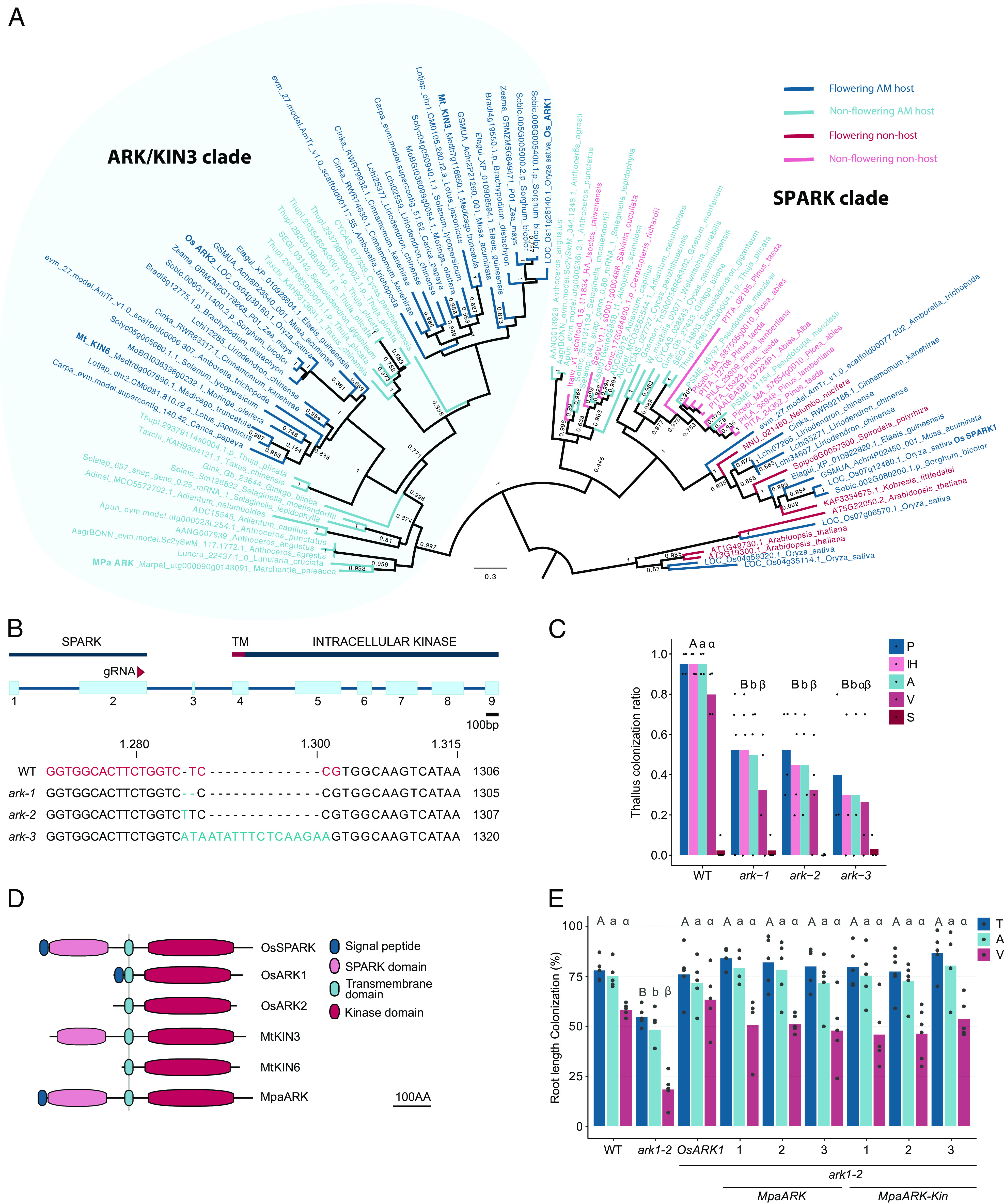
The function of *ARK* in AM symbiosis is evolutionarily in land plants. (*A*). Phylogenetic tree of the URK-2 RLK subfamily comprising the ARK and the SPARK clades. Bootstrap values of important nodes are shown; Dark blue = Angiosperm, Host; Light blue = Nonflowering plant, Host; Magenta = Angiosperm, Nonhost; Pink = Nonflowering plant, Nonhost. (*B*) *MpaARK* gene structure and alignment of independent editing events in three *Mpa_ark* KO lines; exons are boxed and numbered; the position of the gRNA binding site is highlighted by a magenta arrow and magenta text, mutations are highlighted in teal; TM = alpha-helix transmembrane domain. (*C*) AM symbiosis phenotype of WT and *ark* mutant *M. paleacea* thalli at 10 WPI with *R. irregularis*; P = pigment, IH = intracellular hyphae, A = arbuscules, V = vesicles, S = spores; each bar indicates the average ratio of observations of the respective fungal structure over a total of 10, all biological replicates shown; Kruskal–Wallis test was performed to assess significant differences between genotypes, followed by post hoc pairwise comparison using Wilcoxon Rank Sum Test; independent genotypes are grouped into significance classes based on test statistics: Different letters represent significant difference (Wilcoxon Rank Sum, *P*-value > 0.05), “ab” is not significantly different from either “a” or “b.” (*D*) Schematic representation of ARK and SPARK protein structures in *M. paleacea*, *M. truncatula,* and rice. ARK1 and ARK2 in monocots and KIN6 (ARK2) in *M. truncatula* have lost their SPARK domain, which is retained in the homologous SPARK1 clade (31). (*E*) Assessment of the AM symbiosis phenotype of WT, *ark1*−/−, *ark1^OsARK^*, *ark1^MpaARK^*, *and ark1^MpaARK-Kinase^* rice lines inoculated with *R. irregularis,* after 9WPI; each bar indicates the average percentage value of the fungal structure, all biological replicates are shown; T = total colonization, A = arbuscules, V = vesicles; An ANOVA test was performed to compare each fungal structure across genotypes, followed by post hoc comparison using a Tukey's honest significant difference (HSD) test. Different letters represent significant difference (Tukey HSD, *P*-value < 0.05).

We next wished to address the hypothesis that the newly identified set of ancestral AM symbiosis-associated genes are functionally required for AM symbiosis throughout land plants. We concentrated on the *M. paleacea* ortholog of *ARK*, which is exclusively present in AM symbiosis-competent land plants (Fig. 3*A*), and generated CRISPR/Cas9-edited *Mpa_ark* mutants (Fig. 4*B* and *SI Appendix*, Fig. S4*A*).

Nonseed plants share a single copy of *ARK*, while an ancient genome duplication in spermatophytes gave rise to *ARK1* and its closest paralog *ARK2* (Fig. 4*A*) (30). We generated three CRISPR/Cas9 lines carrying indel mutations in the second exon of *MpaARK* (Fig. 4*B*), leading to a premature stop codon ahead of the protein’s transmembrane domain (*SI Appendix*, Fig. S4*A*). Inoculation with *R. irregularis* resulted in 30 to 50% of thallus colonization in all *Mpa_ark KO* alleles, relative to >90% colonization of wild-type *M. paleacea* after 10WPI (Kruskal–Wallis, *P* < 0.05) (Fig. 4*C*). Vesicles occupied 80% of the length of wild type thalli but were only present along 30% thallus length in the three independent *ark* mutant lines (Fig. 4*C*) while arbuscule morphology was equivalent to wild type in all colonized *Mpa_ark* lines (*SI Appendix*, Fig. S6). The *M. paleacea ark* phenocopies rice *ark1* and *ark2*, displaying lower colonization levels, especially for vesicles, while maintaining wild-type arbuscule morphology (30, 32). These findings indicate that the requirement for *ARK* to maintain fungal vigor is evolutionarily conserved across angiosperms and bryophytes.

To address functional conservation, we examined whether the *M. paleacea* ortholog of *ARK1/ARK2* could functionally complement the rice *ark1* mutant. The predicted protein sequence of *MpaARK* encodes an extracellular SPARK (similar protein to ARK) domain, followed by an alpha-helix transmembrane domain and an intracellular active kinase domain (Fig. 4*D*). Monocot *ARK* genes have lost their extracellular *SPARK* domain, which is instead retained in the closely related *SPARK1* clade (Fig. 4*D*) (30). As *MpaARK* encodes a SPARK domain, two variants of the construct were designed, to include 1) the full-length *MpaARK* or 2) the signal peptide, transmembrane, and kinase domains of *MpaARK*, mimicking the domain structure of *OsARK1* (*MpaARK-Kin*) (*SI Appendix*, Fig. S4*B*). As previously reported (32), colonization levels in rice *ark1* were significantly reduced (Fig. 4*E*), which was particularly pronounced for vesicles with 18.8% in the mutant relative to 58.4% in the wild type (TukeyHSD, *P* < 0.05) (Fig. 4*E*). This stark reduction in colonization was fully restored to wild type levels when either wild type *OsARK1*, the full-length *MpaARK*, or the kinase-only *MpaARK-Kin* were introduced in the *ark1* mutant background (TukeyHSD, *P* > 0.05) (Fig. 4*E*). Both the full-length and the kinase-only *MpaARK* sequences also complemented the vesicle phenotype of *ark1* to the same degree as *OsARK1* (TukeyHSD, *P* > 0.05) (Fig. 4*E*), suggesting that the molecular function of this kinase is evolutionarily conserved. Arbuscule morphology was akin to wild type across all genotypes and root types observed, as previously reported for rice *ark1* (30, 32). The combined findings from phylogenetic analysis, transcriptomics, and experimental data presented in this research indicate that the essential role of ARK in regulating symbiotic balance is ancestral to land plants.

## Discussion

We identified a core AM symbiosis genetic programme of 56 genes conserved across land plants, from bryophytes to angiosperms. Overlaying comparative transcriptomic data with phylogenetic analysis further corroborated that a large portion of these genes is also induced during liverwort *M. paleacea* symbiosis, providing evidence for their activity in bryophyte symbiosis. Our findings are supported by earlier phylogenetic studies, which collectively identified 24 of these 56 ECGFs to be conserved in bryophytes (13, 36, 51–53). Among these, functional conservation in bryophyte symbiosis was recently confirmed for the Calcium- and Calmodulin-dependent protein Kinase *CCaMK/DMI3* (13), the transcription factors *CYCLOPS/IPD3* (13) and *MpaWRI* (*MtERF1*) (35), and the strigolactone biosynthesis genes *MAX1*, *CCD8,* and *ZAS* (34).

We found several orthologs of PAM-intrinsic proteins to be conserved in *M. paleacea*, which pushes back the core genetic program associated with arbuscule development and functioning to the MRCA of land plants. In particular, the conservation of the symbiotic ammonium transporter *AMT2* (54) and the symbiotic phosphate transporter *PT4* (24, 25, 55), coupled with evidence that *M. paleacea* is capable of taking up symbiosis-derived phosphate and nitrogen (22), suggests that the molecular mechanisms for symbiotic nutrient uptake are conserved across land plants. Despite previous studies suggesting that phosphate transporters in flowering plants and liverworts have independent origins (36), our analyses point toward a shared origin, indicating that these transporters are in fact orthologs.

Of the 56 ECGFs in *M. paleacea* identified here, 14 lost the ortholog in the symbiosis-incompetent *M. polymorpha*. The early loss of these genes after mutualism abandonment across land plants strongly suggests that their biological function is solely required for AM symbiosis. On the other hand, a second set of 16 genes were found to be symbiosis-specific in angiosperms (21) but conserved across both AM-competent and AM-incompetent bryophytes. The presence of these genes in AM-incompetent bryophytes suggests that they might be required for biological functions that are independent of symbiosis. This is the case for the closest homolog of *VAPYRIN* (*VPY*), *VPY-like*. *VPY-like* is conserved in mycorrhizal angiosperms (40), however, the gene is required for the regulation of a gametophyte developmental stage in the symbiosis-incompetent bryophyte *Physcomitrella patens* (56). *VPY* and other genes within this subgroup might be similarly required in bryophytes for processes other than AM symbiosis, which disappeared in the tracheophyte lineage after the switch from a gametophyte- to a sporophyte-dominant life cycle. Given the recent loss of AM symbiosis in *M. polymorpha* (70.7 to 5 mya) (57), it is also possible that the genetic sequences retained in this nonhost liverwort are undergoing early stages of pseudogenization, as it was previously reported for *CCaMK/DMI3* (13).

Remarkably, orthologs of *ARK* are required to sustain AM symbiosis in *M. paleacea*, rice, and *M. truncatula,* and *ark1* mutants are distinctly characterized by a diminished presence of fungal vesicles in colonized tissue (21, 30). Moreover, the ability of *MpaARK* to fully restore fungal colonization in rice *ark1* mutants confirms that its signaling function is conserved across more than 450 million years of plant evolution and that *ARK* was a component of AM symbiosis in its ancestral form. Therefore, the yet undisclosed mechanism by which *ARK* ensures fungal fitness during symbiosis establishment, but post arbuscule development, is ancestral to land plants. In addition, full-length MpaARK does not hinder downstream signaling at the rice PAM, since the native *MpaARK* sequence was able to rescue the mutant phenotype of *ark1* to the same degree as its variant missing the SPARK domain. We conclude that loss of the SPARK domain from monocot *ARK* genes reflects a decreased reliance on *ARK* for extracellular signaling, a function that may however be provided by other SPARK domain–containing proteins in rice (30).

In summary, this research has revealed an unparalleled degree of conservation across all terrestrial plants of the genetic machinery that underpins AM symbiosis and of its transcriptional regulation. Our data support the view that the common ancestor of embryophytes possessed most of the essential molecular tools required for AM symbiosis as we know it today, as evidenced by the unaltered molecular function of the MpaARK kinase after more than 450 million years of evolution and diversification. The strong natural selection under which *ARK* has been maintained for this extensive period of time is not only indicative of the central importance of its signaling role but also reflecting the substantial benefit that AM symbiosis has provided to land plants throughout their evolution.

## Materials and Methods

### Summary of Plant Material and Growth Conditions for Mycorrhizal Assays.

*M. paleacea* wild type corresponded to the earlier published accession (13). All rice material arose in the *Oryza sativa* ssp. japonica cv. Nipponbare background. For AMS colonization, 4-wk-old *M. paleacea* thalli were transferred to sand pots and inoculated with 4% *R. irregularis* "crude" inoculum (see extended methods in *SI Appendix*). Plants were grown at 22 °C, 16 h day/night cycle with a light intensity of 200 μmol m^−2^ s^−1^ PAR. *M. paleacea* plants were watered three times per week with “artificial rainwater solution” (pH 5.8) (4). For AMS colonization of *O. sativa*, germinated seedlings were transferred into cones containing sand mixed with 300 axenic spores of *R. irregularis*. Rice plants were fertilized twice weekly with Hoagland Solution containing 25 µM KH_2_PO_4_ and grown in 12 h day/night cycles at 28/20 °C and 60% humidity.

The extent of fungal colonization was determined by trypan blue staining. For rice, the previously published protocol was used (32). For *M. paleacea*, thalli were harvested and incubated overnight in 10% (w/v) potassium hydroxide, washed three times with ddH_2_0, incubated in 0.3 M HCl for 15 min at room temperature, then incubated overnight in staining solution [50% (v/v) lactic acid, 25% (v/v) glycerol, 25% (v/v) ddH_2_0, 0.1% (w/v) trypan blue]. For sectioning, stained samples were incubated in destaining solution [50% (v/v) lactic acid, 25% (v/v) glycerol, 25% (v/v) ddH_2_0] for 1 h, then embedded in 3.5% (w/v) agarose gel. Three transverse sections (200 μm) were taken at each of 10 equally distanced positions spanning the length of each thallus, using a Hyrax V50 vibratome (Zeiss, Oberkochen, Germany). Three sections per position were imaged under a Labophot light microscope (Nikon, Minato, Japan) (*SI Appendix,* Fig. S2). For each biological replicate, fungal structures and cell-wall pigment accumulation were quantified by counting their presence or absence at each of the 10 transverse positions imaged. The thallus colonization ratio of biological replicates was calculated by dividing the total number of observations of each structure (x) over the total number of transverse positions imaged (x/10). The following morphological characteristics were quantified with the above method: cell-wall pigment, intracellular hyphae, arbuscules, vesicles, and spores.

Materials and methods are comprehensively detailed in *SI Appendix*.

## Supplementary Material

Appendix 01 (PDF)

Dataset S01 (XLSX)

Dataset S02 (XLSX)

Dataset S03 (PDF)

Dataset S05 (TXT)

Code S01 (R)

## Data Availability

Transcriptome data have been deposited in the National Center for Biotechnology Information Gene Expression Omnibus (GSE245702) (58).
