# Supplementary material for: The receptor-like kinase ARK controls symbiotic balance across land plants
Source: Proc Natl Acad Sci U S A. 2024 Jul 16;121(30):e2318982121. doi: 10.1073/pnas.2318982121 (PMC11287157; doi:10.1073/pnas.2318982121)
Supplement: Supplementary file 1 — Appendix 01 (PDF) [file pnas.2318982121.sapp.pdf]

PNAS

Supporting Information for

The receptor-like kinase ARK controls symbiotic balance across land plants.

Mara Sgroi[a], David Hoey[b], Karina Medina Jimenez[c], Sarah L. Bowden[d], Matthew Hope[d], Emma J. Wallington[d], Sebastian Schornack[b], Armando Bravo[c] and Uta Paszkowski[a]

[a] Crop Science Centre, Department of Plant Sciences, University of Cambridge, 93 Lawrence Weaver Road, Cambridge, CB3 0LE, United Kingdom.

[b] Sainsbury Laboratory, University of Cambridge, Cambridge CB2 1LR, United Kingdom

[c] Donald Danforth Plant Science Center, 975 N. Warson Road, St. Louis, MO, 63132 USA

[d] NIAB, 93 Lawrence Weaver Road, Cambridge, CB3 0LE, United Kingdom

*Mara Sgroi and Uta Paszkowski.

Email: ms2370@cam.ac.uk , up220@cam.ac.uk

This PDF file includes:

Supporting text
Figures S1 to S6
Tables S1 to S6
Legends for Datasets S1 to S3
SI References

Other supporting materials for this manuscript include the following:

Datasets S1 to S3

Extended Methods

Plant materials and growth conditions

Marchantia paleacea wild-type plants were grown axenically on half-strength Gamborg B5 medium (pH 5.8) (Duchefa Biochemie, Haarlem, The Netherlands) on 0.8% (w/v) agar. Plants were grown at 22 °C, with a continuous light intensity of 100 μmol m^{-2} s^{-1} photosynthetically active radiation (PAR). All rice material used in the present study is in *Oryza sativa* ssp. japonica cv. Nipponbare background. The *ark1 TOS17* retro-transposable element-generated insertion allele used in this study corresponds to the previously characterized *ark1-2* mutant line (NF4582) (1, 2). All *OsARK* and *MpaARK* complementation lines were generated in the mutant *ark1-2* background as described below. *O. sativa* seeds were de-husked and sterilized by washing in 70% (v/v) ethanol followed by incubation with 3% (v/v) sodium hypochlorite solution on a platform shaker (Schwabach, Germany) at room temperature (20 min). Seeds were rinsed three times with autoclaved di$H_2$0 before sowing on 0.6% (w/v) agar plates. Seeds were germinated in the dark at 30°C for four days. For seed propagation, seedlings were planted in 0.5L sand pots and watered once a week with RO water and twice a week with half-strength Hoagland solution containing 25 μM KH_2PO_4 and 0.01% (w/v) iron supplement Sequestrene Rapid (Syngenta, Basel, Switzerland). Plants were grown with a 12 h day/night cycle at 28/20 °C in 60% relative humidity.

AMS colonization assay

For AMS colonization of *M. paleacea*, 4-week-old *M. paleacea* thalli were transferred to sand pots (5 × 5 × 6 cm, W × L × H). The AMS pots were inoculated with 4% *R. irregularis* crude inoculum, the mock-inoculated control treatment pots were inoculated with twice-autoclaved 4% (v/v) *R. irregularis* crude inoculum. Crude inoculum was produced by prolonged co-culture of *Tagetes multiflora* and *R. irregularis* (DAOM197198) spores in sand ((3). Inoculated and mock-inoculated plant pots were placed in trays fitted with a closed plastic dome. Plants were grown at 22 °C, 16h day/night cycle with a light intensity of 200 μmol m^{-2} s^{-1} PAR. *M. paleacea* plants were watered three times per week with “artificial rainwater solution” (pH 5.8) (4). For AMS colonization of *O. sativa*, germinated seedlings were transferred into cones (2.5 cm x 12 cm, R x H) containing sand mixed with 300 spores of *R. irregularis* extracted from *Agrobacterium rhizogenes*-transformed carrot hairy root cultures (5).

For trypan blue staining of fungal structures in *M. paleacea*, thalli were harvested at 5, 8 and 11WPI and rinsed in dd$H_2$0. Thalli were incubated overnight in 10% (w/v) potassium hydroxide, washed 3 times with dd$H_2$0, incubated in 0.3 M HCl for 15 min at room temperature, then incubated overnight in staining solution [50% (v/v) lactic acid, 25% (v/v) glycerol, 25% (v/v) dd$H_2$0, 0.1% (w/v) trypan blue]. For sectioning, stained samples were incubated in de-staining solution [50% (v/v) lactic acid, 25% (v/v) glycerol, 25% (v/v) ddH20] for 1 hour, then embedded in

3.5% (w/v) agarose gel. Three transverse sections (200µm) were taken at each of 10 equally distanced positions spanning the length of each thallus, using a Hyrax V50 vibratome (Zeiss, Oberkochen, Germany). Three sections per position were imaged under a Labophot light microscope (Nikon, Minato, Japan) (Fig. S2). For each biological replicate, fungal structures and cell-wall pigment accumulation were quantified by counting their presence or absence at each of the 10 transverse positions imaged. The thallus colonization ratio of biological replicates was calculated by dividing the total number of observations of each structure (x) over the total number of transverse positions imaged (x/10). The following morphological characteristics were quantified with the above method: cell-wall pigment, intracellular hyphae, arbuscules, vesicles, spores.
For trypan blue staining of fungal structures in *O. sativa*, roots were harvested after 7WPI and incubated at 95°C in 10% (w/v) potassium hydroxide (KOH) for 30 min, washed 3 times with ddH20 and incubated in 0.3M HCl at room temperature for 60 min. The roots were removed from HCL and incubated in staining solution (50% (v/v) lactic acid, 25% (v/v) glycerol, 25% (v/v) diH20, 0.1% (w/v) Trypan Blue) at 95°C for 8 min. The staining solution was removed and the root pieces were washed in acidic glycerol (50% (v/v) glycerol, 50% (v/v) 0.3M HCl) to remove excess stain. Ten 2-cm root pieces per biological replicate were mounted onto a glass slide and quantified with a brightfield microscope according to a modified gridline intersect method: the presence of AM fungal structures was recorded along 100 different visual fields using a GXM-L2800 microscope (GT Vision, Stanfield, United Kingdom) and expressed as percentage of the total root length observed (6). For imaging of *M. paleacea* AM colonized sections under confocal laser scanning microscopy (CLSM), 200um sections were treated with Wheat Germ Agglutinin (WGA)-Alexa Fluor™ 488 (Invitrogen, Carlsbad, USA) exhibiting far-red fluorescence. Sections taken using a Hyrax V50 vibratome (Zeiss, Oberkochen, Germany) and incubated in 50% (v/v) ethanol for one hour. Ethanol was removed and 20% (w/v) KOH solution added. After two days, KOH was removed and sections were rinsed with diH2O then submerged in 0.1 M HCL for 1 hour. Roots were then rinsed with 1× phosphate-buffered saline (PBS, pH 7.4) solution and incubated at 4°C in the dark for one week in a 0.2 µg mL^{-1} WGA-Alexa Fluor™ 488 solution in 1× PBS. Visualization of roots took place using a Leica STELLARIS 8 FALCON (Leica Microsystems, Wetzlar, Germany). WGA-Alexa Fluor™ 488 was detected using WLL with an excitation wavelength of 488 nm (10% laser power), emitted wavelengths were collected at 491-533 nm. To detect autofluorescence, an excitation wavelength of 441 nm was used (10% laser power) and emitted wavelengths were collected at 770-790 nm.

Statistical analysis

For pairwise comparisons, sample normal distribution was tested with a Shapiro-Wilk test(7) and equality of variances was assessed with Bartlett's test. The means of samples with normal distribution and equal variances were compared with a Student’s T-test (8). The statistical

difference of samples with non-normal distribution and/or unequal variances were tested with a Mann–Whitney U test(9). Statistically significant differences between multiple groups were assessed using an ANOVA followed by a Tukey's honest significant difference test. If the assumptions of ANOVA were not met by the dataset, statistically significant differences were assessed using the non-parametric Kruskal-Wallis test followed by a pairwise Wilcox post-hoc test. All tests were performed in R (v 4.1.2.). For all tests, the null hypothesis was rejected with a p-value threshold of 0.05. Every graph displays all data points. The exact statistical test used for each dataset is indicated in the corresponding figure legend.

RNA extraction and gene expression analysis by qRT-PCR

For the AMS *M. paleacea* time-course RNAseq and for its independent validation experiment, samples were grown as described in the "AMS colonization assay" section. Samples were harvested after 5, 8 and 11 WPI, washed in distilled water and fixed in ice-cold 100% methanol. For each biological replicate, four thalli were collected and pooled together for subsequent analysis. To maximize the ratio of colonized to non-colonized tissue in each sample, the midribs of collected thalli were excised to remove apical notches, lateral margins and gemma cups as these structures are not colonized by AMS fungi (10). After sectioning, the midribs were washed 3x in ice cold Phosphate-buffered saline (pH 7.4) and snap frozen in liquid nitrogen. The excised midribs were ground in liquid nitrogen with mortar and pestle and total RNA was extracted using PureLink™ Plant RNA Reagent (Thermo Fisher Scientific, Waltham, USA), following manufacturer's instructions. 2% (w/v) polyvinylpyrrolidone (PVP40) was added to the Plant RNA Reagent mixture before extraction to improve RNA purity.

To remove contaminating genomic DNA, one µg of each total RNA sample was treated with Turbo DNA-free DNase (Invitrogen-Thermo Fisher Scientific, Waltham, USA), following manufacturer's instructions. RNA purity was confirmed by PCR with primers spanning the promoter region of *M. paleacea CYCLOPHILIN-B* (Marpal_utg000098g0147841) or the genomic sequence of *O. sativa GAPDH* (LOC_Os08g03290) (Table S1). For qRT-PCR applications, RNA integrity was measured by electrophoresis of 300 ng total RNA in 2.5% (w/v) agarose gel. cDNA was synthesized using SuperScript™ II Reverse Transcriptase according to the manufacturer protocol (Invitrogen, Carlsbad, USA). The total cDNA obtained was diluted 4 folds, then used as a template for qRT-PCR reactions.

The primers in Table S2 were used to amplify the cDNA of their respective target genes by qRT-PCR, with 3 technical replicates per sample, using a standard GoTaq® G2 Flexi reaction mix (Catalog #M7801, Promega, Wisconsin, United States). The qPCR cycle settings were described previously (11). qRT-PCR was performed by measuring the intensity of SYBR Green Fluorescent dye using a C1000 Thermal Cycler with a CFX96 or CFX384 real-time detection system (Bio-Rad Laboratories, Hercules, USA). For each *M. paleacea* sample, the average relative transcript

levels of three technical replicates were normalized against the geometric mean of tree *M. paleacea* housekeeping genes - *MpaACT* (Marpal_utg000003g0005811), *MpaAPT* (Marpal_utg000039g0074951), *MpaELF5* (Marpal_utg000040g0077401) - (Table S2). Expression values are displayed as a function of *MpaACT.*

RNAseq library preparation

For sequencing applications, RNA integrity was assessed using an RNA 6000 pico kit on an Agilent 2100 Bioanalyzer (Agilent Technologies, Santa Clara, USA) and RNA samples with RIN score ≥ 8 were processed into cDNA libraries. cDNA library preparation was performed using 1.1µg of total RNA with a TruSeq Stranded mRNA Library High Throughput Kit (Illumina, San Diego, USA) according to manufacturer's instructions (Catalog #RS-122-9004, DOC, Part #15031047 Rev.E). Library quality was assessed using a DNA1000 chip on a Bioanalyzer 1200 (Agilent Technologies, Santa Clara, USA) and library quantities were measured with a Qubit dsDNA BR Assay Kit (Thermo Fisher Scientific, Waltham, USA). A total of 24 samples from the AMS colonization assay (*R. irregularis* or mock-inoculated conditions, three time points, 3-5 biological replicates) were multiplexed and sequenced on a NextSeq500 (Illumina, San Diego, California, USA) as a 2 × 75nt paired-end run (10 million reads per sample). The NextSeq500/550 High Output v2 Kit (FC-404-2005, Illumina, San Diego, USA) was used for sequencing.

CRISPR/Cas9 constructs and characterization

The CRISPR/Cas9 construct used for targeted gene knock-out in *M. paleacea* was adapted from a previously described construct (*pMpGE010*) designed for gene KO in *M. polymorpha* (12). SgRNA sequences were designed to induce CRISPR-mediated gene editing of the first or second exon of the target gene *MpaARK.* All gRNAs were designed using the CRISPR gDNA Design feature of Geneious® 11.0.4 (13). The best sgRNA sequence (*Mpa_ARK3*) was selected based on its lack of off-target activity (Specificity score = 99.16%) using the *M. paleacea* genome (https://www.ncbi.nlm.nih.gov/datasets/genome/GCA_014180765.2) as off-target database. The sgRNA was also selected based on highest predicted probability of success in gene editing, which was computed by Geneious using a predictive model of sgRNA on-target activity (on-target activity score = 0.55) (14). sgRNA was cloned into the *pMpGE010* destination vector according to the method described in(12, 15).

Rice complementation constructs

The *MpaARK* (*Marpal_utg000090g0143091*) coding sequence was codon-optimized *in silico* for rice using the IDT Codon Optimization Tool (Integrated DNA Technologies, Coralville, Iowa, United States). Two versions of the rice codon-optimized *MpaARK* sequence were designed: a full length *MpaARK* and a shortened version of the *MpaARK* sequence whose SPARK domain

was removed to resemble the SPARK-less rice *ARK1* sequence (Fig. S4*B*). The rice codon-optimized *MpaARK* sequences were ligated *in silico* to the previously described *pOsARK1* promoter (2). For each of the two alternative vector designs (EC85141, EC85143) the full expression cassette was synthesized by GeneArt (Thermo Fisher Scientific, Waltham, USA). Each construct's expression cassette was flanked by Gateway attL1 and L2 sites, which were then used to clone the cassettes ith an LR Clonase II reaction into the binary destination vector *pEW343-R1R2* containing a hyg/hpt cassette for hygromycin selection in tissue culture (Fig. S5) (Table S3). All rice plasmids were transformed into *A. tumefaciens* strain EHA105. *A. tumefaciens* strains were validated by isolating plasmid DNA, retransforming into *E. coli* and restriction digestion prior to transformation of rice callus. All hygromycin-resistant positive transformant lines harboring either *pEW429, pEW430* or *pGWB553+AM14pg\r* inserts were potted into sand, genotyped for the presence of the correct transformation cassette (Table S3) and grown for seed setting.

Plant transformation

M. paleacea constructs were transformed into *Agrobacterium tumefaciens* GV3101 (pMP90) by electroporation. For thallus transformation, *M. paleacea* was grown on M-Media (5) under continuous light (70 μmol m^{-2} s^{-1} PAR) at 22°C for three weeks before cutting. Transformation was then carried out using the *Agrobacterium*-mediated thallus regeneration method (16). The sucrose treatment and selection steps were carried out on ½ MS Media (pH 5.6), the latter step supplemented with cefotaxime (125 μg/mL) and hygromycin B (15-25 μg/mL). Stable transgenic lines were selected and propagated from gemmae and were genotyped from the G1 generation onwards.

All rice binary plasmids designed in this study (Table S3) and the previously described pGWB553+AM14pg\r construct (2) were transformed using mature seed-derived callus from rice *ark1*-2 mutant background (NF4582)(1, 2). Mature dehusked seeds were surface sterilized, thoroughly rinsed with sterile deionized water and air dried in a flow bench. The embryo axes were removed and seeds plated on N6DT medium essentially as previously described (17) supplemented with timentin (150 mg l^{-1}). Plates were sealed, cultured in the dark at 28 °C for 17 days and subcultured 4 days prior to transformation. Callus pieces were inoculated with an overnight culture of *A. tumefaciens* strain EHA105, containing the appropriate construct, resuspended in AAM medium (0.2–0.3 OD600). After 5 min, the *A. tumefaciens* suspension was removed and the inoculated callus transferred to sterile filter paper in a 9 cm petri dish. Plates were sealed with Parafilm and co-cultivated for 3 days at 25 °C/23 °C in the dark, followed by transfer to N6DT with 50 mg l−1 hygromycin. Subsequent tissue culture media were essentially as described previously (18) except for the substitution of NB basic with MS salts and of cefotaxime/vancomycin with timentin, as above. Callus pieces were transferred to fresh medium

at 10-day intervals with shoots removed for rooting after approximately 4–5 weeks. Rooting was undertaken on HF medium with timentin and 25 mg l −1 hygromycin. Genomic DNA was isolated from rooted plantlets (19) and plants were confirmed as transformed by multiplex PCR using primers for the hpt selectable marker gene (Hyg-UP) (Table S1) and a cyclophilin endogenous control gene (OsCYP2_PP) (Table S1) by PCR with Faststart Taq polymerase (Roche, Basel, Switzerland) as recommended by manufacturer (annealing temperature 58°C).

Genotyping

Genomic DNA was used as a template for genotyping by PCR with GoTaq® G2 DNA Polymerase (Promega) in a PTC-225 Peltier thermal cycler (Watertown, USA), unless stated otherwise. PCR conditions were: 95°C initial denaturation (3 min), 30 cycles of - denaturation (30s) at 95°C, annealing (60s) at temperature adjusted to the melting temperature of the primers (Table S1), extension (30-180s) at 72°C- followed by final extension (2 min) at 72°C. PCR products were run through agarose gel electrophoresis (1% w/v agarose, 1 µg mL^{-1} ethidium bromide) at 120mV (30min) and visualized under UV light to confirm product sizes. Images were acquired using a GBox Chemi 16 Bio Imaging System (Syngene, Cambridge, United Kingdom). To confirm the genotype of CRISPR/Cas9-edited *M. paleacea* plants, PCR products were excised and purified using the NucleoSpin® Gel and PCR Clean-up kit (Macherey-Nagel, Düren, Germany) following manufacturer’s instructions. A total of 250-500 ng μL^{-1} of DNA per sample were sequenced through Sanger sequencing (Source BioScience, Cambridge, United Kingdom).

Differential expression analysis

Raw RNAseq reads were quality-filtered to remove low quality reads using FastQC v0.12. Adaptor sequences were trimmed with Trim Galore v0.6.7 (20). All sample libraries were mapped to the *M. paleacea* reference genome (https://www.ncbi.nlm.nih.gov/datasets/genome/GCA_014180765.2) (21) with STAR 2.7.11a (22). Mapped reads were counted with Subread (v2.0.6) using the featureCounts function (23). Only genes with a minimum total of ten reads across all timepoints/conditions were considered for downstream analysis. All *M. paleacea* gene IDs mentioned in this article refer to the reference genome (21). Differential expression analysis was performed with the DESeq2 v1.22.2 (24) R package. Differentially expressed genes were identified by pairwise comparison of mock-inoculated vs. mycorrhizal samples at the same time-point, with a significance threshold of log2 fold-change > |1| and adjusted p-value < 0.05.

Phylogenetic analysis

A reference list of genes associated with AM symbiosis from published literature was employed to identify orthologous genes in *M. paleacea* (Table S4). These genes were subjected to individual

BLAST searches against a local protein model database containing sequences from 59 publicly available plant genomes, encompassing 29 non-seed plant genomes and 30 representative seed plant genomes (Table S5). Alignments of the top 500 BLAST hits for each gene were generated using MAFFT, and columns with fewer than 50% characters across the 500 genes were excluded. The trimmed alignments were then used to construct phylogenetic trees employing FastTree or PhyML with the wag substitution model. Visualization and color coding of the trees were accomplished using FigTree.

Gene ontology enrichment analysis

GO enrichment analysis was performed with OmixBox (25), using a two-tailed Fisher's exact test with p-value correction by False Discovery Rate (FDR) control according to Benjamini-Hochberg (26). The list of enriched GO terms was further reduced by only retaining terms with the lowest hierarchical level in the GO directed acyclic graph. For each RNAseq timepoint, the set of significantly upregulated/downregulated genes was compared to the full reference *M. paleacea* transcriptome. Only GO terms with more than 4 genes in the test set were analysed and the significant threshold for GO enrichment was set as FDR < 0.05.

Comparative transcriptomic analysis

M. truncatula orthologs of candidate *M. paleacea* genes were identified through phylogenetic analysis as described in the relevant section. For the *M. truncatula*-*M. paleacea* comparison, the expression of the *M. paleacea* ortholog in the AMS RNAseq dataset was directly compared to the expression of its *M. truncatula* orthologs at the timepoint with the strongest response to AMS in Luginbuehl *et al*.'s RNAseq dataset (27DPI) (27) (Table S6). Given the difference in methodology for the generation and analysis of the two RNAseq experiments, we adopted the list of *M. truncatula* DE genes as defined by the authors (27).

Figures and Tables

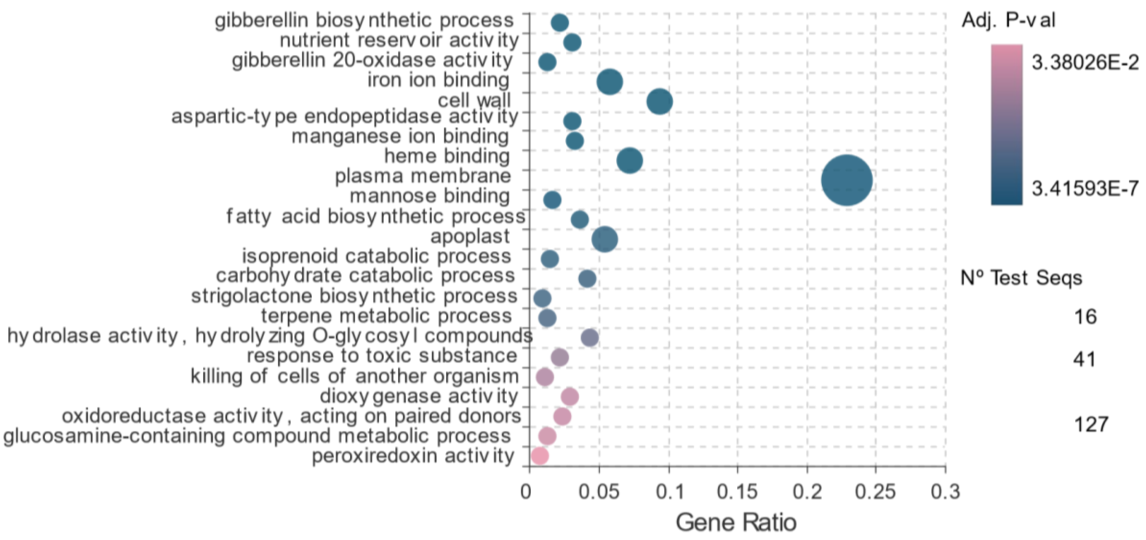

Fig. S1. GO term enrichment of *M. paleacea* genes upregulated after 11 weeks post-inoculation with *R. irregularis.* Enrichment analysis was performed with Fisher's exact test. Heatmap colors represent adjusted Fisher P-values for each GO term, only terms with adjusted P-value > 0.05 and at least 3 genes in the test list are considered significant.

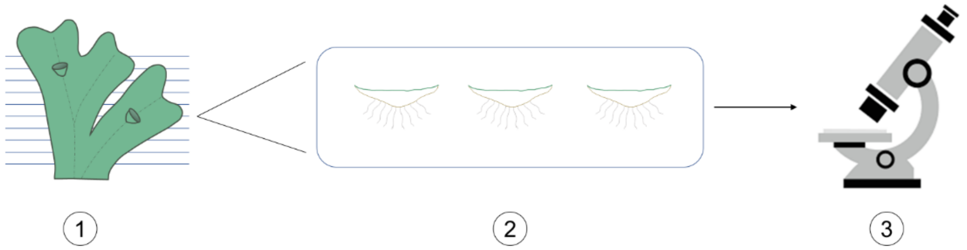

Fig. S2. Imaging and quantitative assessment of arbuscular mycorrhizal fungal structures stained with trypan blue. (1) Thalli are embedded in 3.5% agarose, transversal sections (200μm) are taken at 10 equally distanced sites spanning the length of each thallus but excluding the apical notch; (2) three representative sections are taken for each site and mounted onto a microscope slide; (3) For each slide, the presence/absence of the following structures is noted in a scoring matrix: cell wall pigment, intracellular hyphae, arbuscules, vesicles, spores.

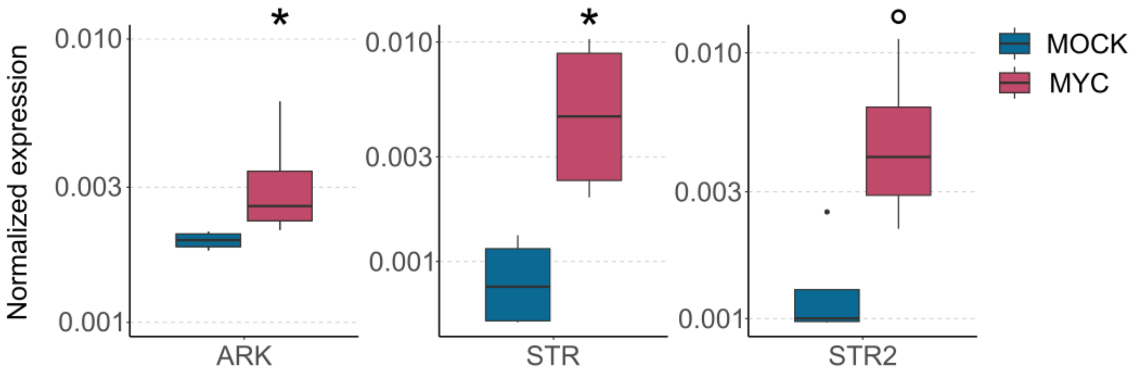

Fig. S3. Gene expression of *M. paleacea* AMS-responsive genes to *R. irregularis* colonization at 5 weeks-post inoculation (5 WPI). Expression levels for each gene were normalized relative to the expression of *MpaACTIN 7*; MOCK = mock-inoculated control samples; MYC= R. irregularis inoculated samples; n = 4. * = Mann–Whitney U test P-value < 0.05; ° = Mann–Whitney U test P-value = 0.057.

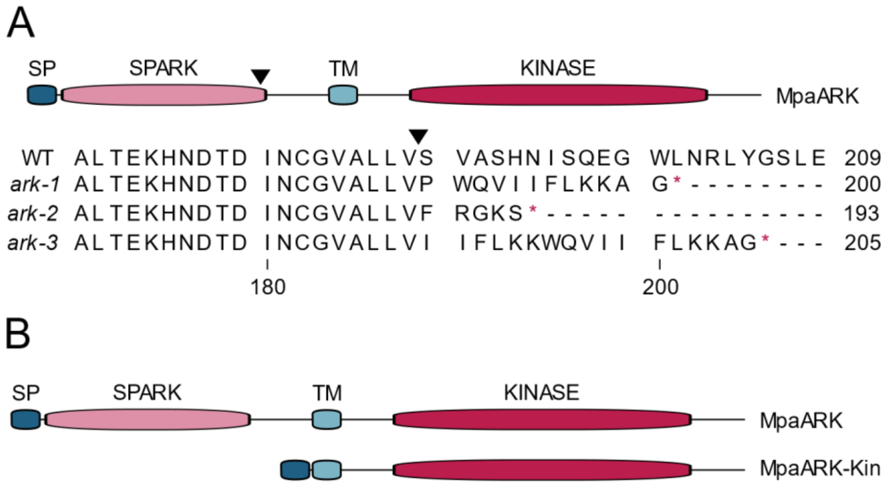

Fig. S4. MpaARK protein structure in WT and *ark M. paleacea* and in complemented *O. sativa*. (*A*) Predicted protein sequence change in the three independent lines harbouring nonsense mutations in the second exon of *MpaARK*; predicted premature stop codons are highlighted in magenta; Black arrow = start of frameshift, SP = signal peptide, TM = transmembrane domain. (*B*) Schematic representation of the codon-optimized *M. paleacea ARK* constructs designed to complement rice *ark1* mutants. All constructs are driven by the endogenous rice *ARK1* promoter. Vector EC85141 encodes the full-length *MpaARK* gene; Vector EC85143 encodes the signal peptide (SP), the transmembrane domain (TM) and the intracellular kinase domain of *MpaARK*, while the SPARK domain was removed to resemble the native *OsARK1* protein structure.

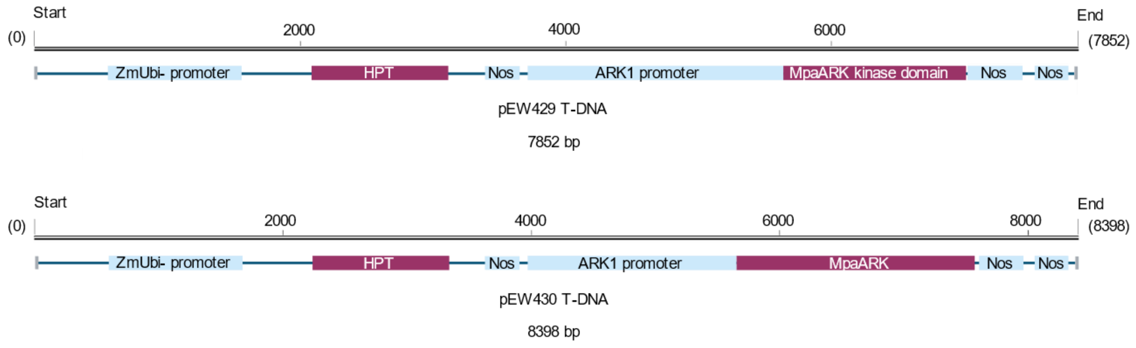

Fig. S5. T-DNA inserts harbouring rice codon-optimized *M. paleacea ARK* sequences. Both pEW429 and pEW430 vectors were transformed into rice *ark1-2* background; ZmUbi: *Zea mays* ubiquitin; HPT: hygromycin phosphotransferase gene.

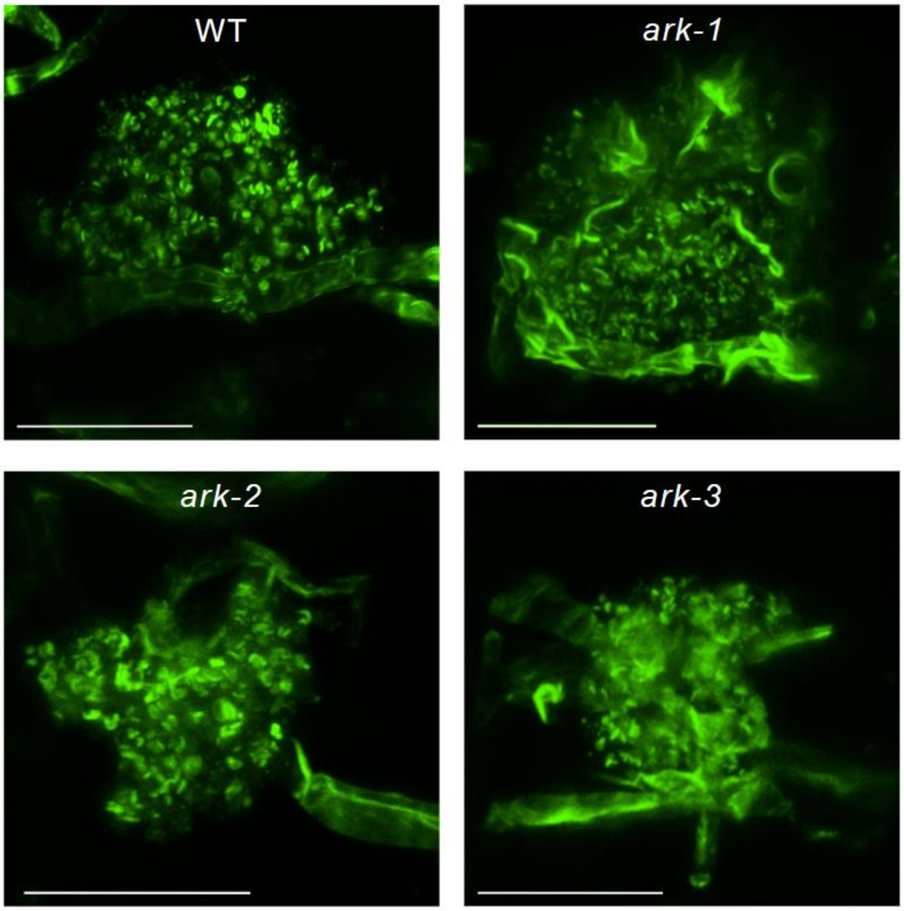

Fig. S6. Confocal microscope images of fully developed *Rhizophagus irregularis* arbuscules in *Marchantia paleacea* thalli after 10 weeks post-inoculation. Fungal structures were stained with wheat germ agglutinin (WGA)-Alexa Fluor 488. Representative images per genotype are provided, scale bar = 20 μm.

Table S1. Primer pairs for genotyping *M. paleacea* and rice transformant lines. Sequences in bold are forward primers, others represent reverse primers; **co-** rice codon optimized.

Primer pair	Target	Primer Sequences	Product size
**OsARK1-WT**	*O. sativa*<br>WT OsARK1 allele	**GGGATTGTGCATCTGTCTTGAGG**<br>CCGCAATTTTCAATATCAACACGA	981 bp
**OsARK1-TOS17**	*O. sativa*<br>TOS17 insertion in OsARK1 allele	**GGGATTGTGCATCTGTCTTGAGG**<br>GACAACACCGGAGCTATACAAATCG	660 bp
**Hyg-UP**	*pEW343-R1R2*<br>hyg/hpt cassette	**GTTTATCGGCACTTTGCATCGGCCG**<br>GATTTGTGTACGCCCGACAGTCC	-
**OsCYP2_PP**	*O. sativa*<br>WT cyclophilin	**TCCCAGTTCTTCATCTGCAC**<br>GCGATATCATAGAAGCAGCGAC	416 bp
**Mpa_ArkEC_PP1**	*M. paleacea* full-length *co-ARK* CDS	**ACACAGCTCAGGTAAAAGGGAG**<br>AGCTTAACCACCATGACGGG	805 bp
**Mpa_ArkEC_PP1**	*M. paleacea* kinase-only *co-ARK* CDS	**ACACAGCTCAGGTAAAAGGGAG**<br>AGCTTAACCACCATGACGGG	259 bp
**mRFP_PP1**	*O. sativa*<br>*ARK1:mRFP* CDS	**CCCCTCAGTTCCAGTACGGC**<br>AAGTTCATCACGCGCTCCCA	112 bp
**OsGAPDH_gen**	*O. sativa*<br>gDNA contamination in total RNA samples	**AGGTTCTTCCTGATTTGAATGG**<br>CAACTGCACTGGACGGCTTA	1057 bp
**MpaCYPb**	*M. paleacea*<br>gDNA contamination in total RNA samples	**AAGGGACTCTGGCTCTCACT**<br>GATGCTGCCGATGCTCA	233bp
**MpU6pro-PP**	*MpU6* promoter in pMpGE010 inserts	**TGCAGTCCATAACGTATCACTCT**<br>GATCACGATGGGGACTTGTACA	289 bp
**AtcoCAS9-PP**	*AtcoCAS9* CDS in pMpGE010 backbone	**TTTCGGAAACTTGATCGCTCTC**<br>AAGAGCCTTGAGAAGTGTGAGA	304 bp
**CRISPR_gARK3_PP3**	sgRNA binding site in *MpaARK* CDS	**GTGTGCAAACTTGGACAAGGA**<br>ACAATCGATTCAACCAGCCTTC	185bp

Table S2. Primer pairs for amplification of *M. paleacea* transcripts by qRT-PCR. Sequences in bold are forward primers, the others represent reverse primers.

Primer pair	Application	Sequences	Amplicon size
**MpaACT-1**	qRT-PCR<br>Reference gene	**AGGCATCCGGTATCCATGAG**<br>ACATGGTGGTTCCTCCAGAC	108 bp
**MpaAPT-1**	qRT-PCR<br>Reference gene	**CGAAAGCCCAGGAAGCTACC**<br>GTACCCCCCGTTGCAATAAG	146 bp
**MpaELF5-2**	qRT-PCR<br>Reference gene	**AGGAAGGATTTGGCGAAGGA**<br>CTGACCAAGGGCAAGTACGA	130 bp
**MpaARK_qPP2**	*MpaARK* expression	**AGCGTGGCGACTTGCTAGACT**<br>CCGTCCTCAGACACTGGCGA	99 bp
**MpaSTR_qPP3**	*MpaSTR* expression	**CTGAAGAACCCGTCTTGCCTGC**<br>TTACCGCTCAGAAAGCACGCC	97 bp
**MpaSTR2_qPP3**	*MpaSTR2* expression	**AGTACCCATACGAAGCTCTGCTGC**<br>GACCTGCGCGGCACGGAAATA	80 bp

Table S3. List of constructs used to complement the mutant phenotype of rice *ark1* -/- lines. *pOsARK1*: endogenous *ARK1* promoter; **CDS**: coding sequence; **C-Tag**: C-terminal protein tag; **mRFP**: monomeric red fluorescent protein; **Term**: transcription terminator;

Vector	Insert	Promoter	CDS	C-Tag	Term	Origin
**pEW430**	EC85141	pOsARK1	*MpaARK*		NosT	This study
**pEW429**	EC85143	pOsARK1	*MpaARK-kinase only*		NosT	This study
**pGWB553+AM14pg\r**	-	pOsARK1	*OsARK*	mRFP	NosT	(2)

Table S4. List of 103 gene families investigated. The homology column indicates whether a ortholog or homolog of the gene family was identified in *M. paleacea*

Gene family	*Medicago* ID	*M. paleacea* ID	Homology	*M. polymorpha* ortholog	*Co-elimination in non-hosts*
AMPa	Medtr3g111900	Marpal_utg000021g0055731	ortholog	no	Land plants
AMT2	Medtr7g115050	Marpal_utg000013g0032291	proto-ortholog	no	Land plants
ARK/KIN3/KIN6	Medtr7g116650	Marpal_utg000090g0143091	proto-ortholog	no	Land plants
DHY	Medtr4g097510	Marpal_utg000167g0186021	ortholog	no	Land plants
DMI2/SYMRK	Medtr5g030920	Marpal_utg000051g0090241	ortholog	no	Land plants
EPP1/HYP	Medtr3g099200	Marpal_utg000002g0001491	ortholog	no	Land plants
ERF1/AP2B/WRI	Medtr7g009410	Marpal_utg000074g0124251	ortholog	no	Land plants
IPD3/CYCLOPS	Medtr5g026850	Marpal_utg000051g0091871	ortholog	no	Land plants
PP2A	Medtr1g112940	IHWO_myc10130	ortholog	no	Land plants
RAD1	Medtr4g104020	Marpal_utg000070g0119331<br>Marpal_utg000071g0120271	ortholog	no	Land plants
RFC	Medtr5g020810	Marpal_utg000064g0107131	proto-ortholog	no	Land plants
STR	Medtr8g107450	Marpal_utg000139g0174371	ortholog	no	Land plants
STR2	Medtr5g030910	Marpal_utg000110g0155891	ortholog	no	Land plants
ABCB20	Medtr3g093430	Marpal_utg000130g0170881<br>Marpal_utg000033g0068061	ortholog	yes	Land plants
AMPb	Medtr2g098490	Marpal_utg000005g0011521<br>Marpal_utg000013g0034161	ortholog	yes	Land plants
DUF538	Medtr2g091210	Marpal_utg000056g0097771	ortholog	yes	Land plants
HYP2	Medtr3g467150	Marpal_utg000070g0118111	ortholog	yes	Land plants
KIN5	Medtr3g104900	Marpal_utg000012g0029631	ortholog	yes	Land plants
LYK10	Medtr5g033490	Marpal_utg000156g0182581	ortholog	yes	Land plants
VPY	Medtr6g027840	Marpal_utg000143g0177401	ortholog	yes	Land plants
ZAS/CCD	Medtr3g110195	Marpal_utg000010g0020551<br>Marpal_utg000077g0127141<br>Marpal_utg000099g0148701	ortholog	yes	Land plants
AP2A	Medtr7g011630	Marpal_utg000040g0076701<br>Marpal_utg000001g0000321	ortholog	yes	Angiosperms
CASTOR	Medtr7g117580	Marpal_utg000046g0082721	proto-ortholog	yes	Angiosperms
EXO	Medtr1g017910	Marpal_utg000068g0113951	proto-ortholog	yes	Angiosperms
HYP3	Medtr4g104750	Marpal_utg000055g0096851	ortholog	yes	Angiosperms
LEA	Medtr2g088610	Marpal_utg000068g0113121<br>Marpal_utg000038g0074401	ortholog	yes	Angiosperms
PT4	Medtr1g028600	Marpal_utg000069g0117021	ortholog	yes	Angiosperms
RAM2	Medtr1g040500	Marpal_utg000108g0154471<br>Marpal_utg000148g0178951<br>Marpal_utg000100g0149511	ortholog	yes	Angiosperms
SYP132	Medtr2g088700	Marpal_utg000126g0168881<br>Marpal_utg000074g0124311	proto-ortholog	yes	Angiosperms
ABCB12	Medtr8g022270	n.a.	homologs	n.a.	Angiosperms
AP2C	Medtr2g016730	n.a.	homologs	n.a.	Angiosperms
AP2D	Medtr8g032610	n.a.	homologs	n.a.	Angiosperms
BCP	Medtr1g090420	n.a.	homologs	n.a.	Angiosperms
CBF	Medtr2g081600	n.a.	homologs	n.a.	Angiosperms
CYTB561	Medtr1g033360	n.a.	homologs	n.a.	Angiosperms
CYTP450	Medtr6g034940	n.a.	homologs	n.a.	Angiosperms
DIP1	Medtr8g093070	n.a.	homologs	n.a.	Angiosperms
DNAJ	Medtr2g008520	n.a.	homologs	n.a.	Angiosperms
DUF4228	Medtr2g104800	n.a.	homologs	n.a.	Angiosperms
FATM	Medtr1g109110	n.a.	homologs	n.a.	Angiosperms
GDSL	Medtr8g074560	n.a.	homologs	n.a.	Angiosperms
GER	Medtr2g086620	n.a.	homologs	n.a.	Angiosperms
GRAS	Medtr1g069725	n.a.	homologs	n.a.	Angiosperms
HEP	Medtr1g062970	n.a.	homologs	n.a.	Angiosperms
HYP4	Medtr1g069620	n.a.	homologs	n.a.	Angiosperms
KELCH	Medtr2g012790	n.a.	homologs	n.a.	Angiosperms
KINA	Medtr2g023150	n.a.	homologs	n.a.	Angiosperms
KINB	Medtr3g064080	n.a.	homologs	n.a.	Angiosperms
KINC	Medtr4g126930	n.a.	homologs	n.a.	Angiosperms
KIND	Medtr8g092290	n.a.	homologs	n.a.	Angiosperms
KINE	Medtr3g088855	n.a.	homologs	n.a.	Angiosperms
KINH	Medtr4g129010	n.a.	homologs	n.a.	Angiosperms
MDR1	Medtr3g086430	n.a.	homologs	n.a.	Angiosperms
MLO	Medtr3g115940	n.a.	homologs	n.a.	Angiosperms
MYB1	Medtr7g068600	n.a.	homologs	n.a.	Angiosperms
NFP	Medtr5g019040	n.a.	homologs	n.a.	Angiosperms
NTR1	Medtr2g017750	n.a.	homologs	n.a.	Angiosperms
P450	Medtr3g051230	n.a.	homologs	n.a.	Angiosperms
PCL	Medtr7g086190	n.a.	homologs	n.a.	Angiosperms
QI	Medtr5g043550	n.a.	homologs	n.a.	Angiosperms
RAM1	Medtr7g027190	n.a.	homologs	n.a.	Angiosperms
SEC	Medtr7g087500	n.a.	homologs	n.a.	Angiosperms
CBP	Medtr6g043700	n.a.	unsolved	n.a.	Angiosperms
GST	Medtr5g076900	n.a.	unsolved	n.a.	Angiosperms
HMAD	Medtr5g020960	n.a.	unsolved	n.a.	Angiosperms
HYP5c	Medtr8g069400	n.a.	unsolved	n.a.	Angiosperms
HYP6	Medtr8g040940	n.a.	unsolved	n.a.	Angiosperms
RHI	Medtr1g023770	n.a.	unsolved	n.a.	Angiosperms
SIN	Medtr3g020800	n.a.	unsolved	n.a.	Angiosperms
SMALL1	Medtr5g018610	n.a.	unsolved	n.a.	Angiosperms
DMI3/CCaMK	Medtr8g043970	Marpal_utg000137g0173321	ortholog	no	no
MAX1/CYP711A	Medtr3g104560	Marpal_utg000021g0055761<br>Marpal_utg000021g0055741	ortholog	no	no
CCD1	Medtr8g037310	Marpal_utg000106g0154041	ortholog	yes	no
CERBERUS	Medtr1g090320	Marpal_utg000112g0157671<br>Marpal_utg000041g0079031	ortholog	yes	no
D14	Medtr1g018320	Marpal_utg000147g0178301	ortholog	yes	no
D14L/KAI2	Medtr4g095310	Marpal_utg000015g0039791	ortholog	yes	no

D27	Medtr1g471050	Marpal_utg000152g0180621	ortholog	yes	no
DELLA1	Medtr3g065980	Marpal_utg000095g0145961<br>Marpal_utg000103g0151291	ortholog	yes	no
DMI1/POLLUX	Medtr2g005870	Marpal_utg000046g0082721	ortholog	yes	no
DXS2a	Medtr8g068300	Marpal_utg000002g0002531	ortholog	yes	no
HA1	Medtr8g006790	Marpal_utg000008g0017601	ortholog	yes	no
GA20ox	Medtr1g102070	Marpal_utg000015g0038871<br>Marpal_utg000039g0074971<br>Marpal_utg000011g0026241<br>Marpal_utg000012g0030651	ortholog	yes	no
KO	Medtr2g105360	Marpal_utg000013g0032101<br>Marpal_utg000013g0032051<br>Marpal_utg000021g0055821	ortholog	yes	no
LYK3/CERK	Medtr5g086130	Marpal_utg000050g0087701	ortholog	yes	no
MAX2/D3	Medtr4g080020	Marpal_utg000086g0138511	ortholog	yes	no
MAX3/CCD7	Medtr7g045370	Marpal_utg000013g0034951	ortholog	yes	no
MAX4/CCD8	Medtr3g109610	Marpal_utg000049g0083651<br>Marpal_utg000036g0072591<br>Marpal_utg000010g0024761	ortholog	yes	no
MCA8	Medtr7g100110	Marpal_utg000088g0140181	ortholog	yes	no
MSBP1	Medtr6g054890	Marpal_utg000062g0105641	ortholog	yes	no
NENA	Medtr6g072020	Marpal_utg000034g0069021	ortholog	yes	no
NOPE1	Medtr3g093270	Marpal_utg000054g0094671<br>Marpal_utg000018g0051451	ortholog	yes	no
NSP1	Medtr8g020840	Marpal_utg000012g0028161	ortholog	yes	no
NSP2	Medtr3g072710	Marpal_utg000049g0085231	ortholog	no	no
NUP133	Medtr5g097260	Marpal_utg000023g0058101	ortholog	yes	no
NUP85	Medtr1g006690	Marpal_utg000023g0058261	ortholog	yes	no
SUNN	Medtr4g070970	Marpal_utg000071g0119821	ortholog	yes	no
SUT2	Medtr8g468330	Not in genome	ortholog	yes	no
CNCG15b	Medtr4g058730	n.a.	homologs	n.a.	no
HA9	Medtr7g117500	n.a.	homologs	n.a.	no
PDR1	Medtr1g011650	n.a.	homologs	n.a.	no
PUB1	Medtr5g083030	n.a.	homologs	n.a.	no
SbtM1a	Medtr5g011320	n.a.	homologs	n.a.	no
VAMP721d	Medtr2g028790	n.a.	homologs	n.a.	no

Table S5. List of genomic resources used as input for phylogenetic analysis. AM = arbuscular mycorrhiza host; Non-AM = arbuscular mycorrhiza non-host

Scientific Name	Protein models	Abbreviation	Taxa	Group	AM	Origin
Chlorokybus atmophyticus	9,300	Cat	Streptophyta	Streptophyta	Non-AM	genome.jgi.doe.gov
Mesostigma viride	9,300	Mvi	Streptophyta	Streptophyta	Non-AM	genome.jgi.doe.gov
Klebsormidium flaccidum	17,055	Kfl	Streptophyta	Streptophyta	Non-AM	genome.jgi.doe.gov
Chara braunii	35,885	Cbr	Streptophyta	Streptophyta	Non-AM	genome.jgi.doe.gov
Mesotaenium endlicherianum	11,080	Men	Streptophyta	Streptophyta	Non-AM	genome.jgi.doe.gov
Penium margaritaceum	53,270	Pma	Streptophyta	Streptophyta	Non-AM	NCBI
Spirogloea muscicola	27,137	Smu	Streptophyta	Streptophyta	Non-AM	genome.jgi.doe.gov
Anthoceros angustus	14,629	Aan	Hornwort	Bryophyta	AM	CNGB
Anthoceros agrestis	39,981	Aag	Hornwort	Bryophyta	AM	www.hornworts.uzh.ch
Anthoceros punctatus	33,187	Apu	Hornwort	Bryophyta	AM	www.hornworts.uzh.ch
Marchantia paleacea	18,769	Mpa	Liverwort	Bryophyta	AM	Rich et al 2020
Marchantia inflexa	23,441	Min	Liverwort	Bryophyta	AM	Marks et al. 2019
Marchantia polymorpha	19,287	Mpo	Liverwort	Bryophyta	Non-AM	Marchantia.info
Lunularia cruciata	28,212	Lcr	Liverwort	Bryophyta	AM	CoGe
Calohypnum plumiforme	30,237	Cpl	moss	Bryophyta	Non-AM	ibi.zju.edu.cn
Ceratodon purpureus	31,482	Cpu	moss	Bryophyta	Non-AM	Phytozome13
Entodon seductrix	25,801	Ese	moss	Bryophyta	Non-AM	CNGB
Fontinalis antipyretica	16,538	Fan	moss	Bryophyta	Non-AM	Gigadb
Hypnum curvifolium	29,077	Hcu	moss	Bryophyta	Non-AM	CNGB
Physcomitrella patens	26,610	Ppa	moss	Bryophyta	Non-AM	phytozome10
Pleurozium schreberi	21,464	Psc	moss	Bryophyta	Non-AM	Pederson et al. 2019
Pohlia nutans	40,905	Pnu	moss	Bryophyta	Non-AM	CNGB
Syntrichia caninervis	17,202	Sca	moss	Bryophyta	Non-AM	NCBI
Sphagnum fallax	25,100	Sfa	moss	Bryophyta	Non-AM	Phytozome13
Sphagnum magellanicum	25,227	Sma	moss	Bryophyta	Non-AM	Phytozome13
Isoetes taiwanensis	41,199	Ita	Lycophyte	Tracheophyta	Non-AM	CoGe
Selaginella lepidophylla	27,204	Sle	Lycophyte	Tracheophyta	AM	NCBI
Selaginella moellendorffii	22,273	Smo	Lycophyte	Tracheophyta	AM	phytozome10
Azolla filiculoides	20,203	Afi	Fern	Tracheophyta	Non-AM	Fernbase
Salvinia cucullata	19,779	Scu	Fern	Tracheophyta	Non-AM	Fernbase
Ceratopteris richardii	36,857	Cri	Fern	Tracheophyta	Non-AM	Phytozome13
Adiantum capillus	31,244	Aca	Fern	Tracheophyta	AM	Fernbase
Adiantum nelumboides	68,601	Ane	Fern	Tracheophyta	AM	NCBI
Alsophila spinulosa	71,488	Asp	Fern	Tracheophyta	AM	Fernbase
Marsilea vestita	22,541	Mve	Fern	Tracheophyta	AM	Fernbase
Abies Alba	97,750	Aal	Gymnospermae	Gymnospermae	Non-AM	treegenesdb.org
Ginkgo biloba	41,309	Gbi	Gymnospermae	Gymnospermae	AM	ginkgo.zju.edu.cn
Gnetum montanum	27,491	Gmo	Gymnospermae	Gymnospermae	AM	bioinformatics.psb.ugent.be
Welwitschia mirabilis	26,990	Wmi	Gymnospermae	Gymnospermae	AM	CNGB
Cycas panzhihuaensis	32,353	Cyp	Gymnospermae	Gymnospermae	AM	CNGB
Picea abies	66,632	Pab	Gymnospermae	Gymnospermae	Non-AM	congenie.org
Pinus lambertiana	38,518	Pla	Gymnospermae	Gymnospermae	Non-AM	treegenesdb.org
Pseudotsuga menziesii	51,419	Pme	Gymnospermae	Gymnospermae	AM	bioinformatics.psb.ugent.be
Pinus taeda	51,751	Pta	Gymnospermae	Gymnospermae	Non-AM	bioinformatics.psb.ugent.be
Taxus chinensis	44,770	Tch	Gymnospermae	Gymnospermae	AM	NCBI
Sequoiadendron giganteum	41,631	Sgi	Gymnospermae	Gymnospermae	AM	treegenesdb.org
Thuja plicata	39,659	Tpl	Gymnospermae	Gymnospermae	AM	Phytozome13
Amborella trichopoda	26,846	Atr	Basal	Basal	AM	amborella.org
Brachypodium distachyon	31,694	Bdi	Monocot	Monocot	AM	phytozome10
Musa acuminata	36,519	Mac	Monocot	Monocot	AM	gramene.org
Oryza sativa	39,049	Osa	Monocot	Monocot	AM	phytozome10
Phoenix dactylifera	28,889	Pda	Monocot	Monocot	AM	qatar-weill.cornell.edu
Spirodela polyrhiza	19,623	Spo	Monocot	Monocot	Non-AM	phytozome10
Zea mays	63,480	Zma	Monocot	Monocot	AM	phytozome10
Zostera marina	20,648	Zos	Monocot	Monocot	Non-AM	Phytozome13
Arabidopsis thaliana	35,386	Ath	Dicot	Dicot	Non-AM	phytozome10
Beta vulgaris	29,831	Bvu	Dicot	Dicot	Non-AM	bvseq.molgen.mpg.de
Dianthus caryophyllus	56,382	Dca	Dicot	Dicot	Non-AM	carnation.kazusa.or.jp.
Glycine max	54,175	Gma	Dicot	Dicot	AM	phytozome10
Lupinus angustifolius	31,408	Lan	Dicot	Dicot	Non-AM	genbank
Lotus japonicus	42,399	Lja	Dicot	Dicot	AM	kazusa.or.jp/lotus/
Medicago truncatula	62,319	Mtr	Dicot	Dicot	AM	jcvi.org
Nelumbo nucifera	26,685	Nnu	Dicot	Dicot	Non-AM	genomevolution.org
Solanum lycopersicum	34,727	Sly	Dicot	Dicot	AM	Solgenomics
Utricularia gibba	28,494	Ugi	Dicot	Dicot	Non-AM	genomevolution.org

Table S6. Expression pattern of *M. paleacea* and *M. truncatula* orthologs at different stages of arbuscular mycorrhiza symbiosis. *M. truncatula* expression data (grey) was sourced from (27) ; L2FC = Log2 fold change; WPI = weeks post inoculation; DPI = days post inoculation; Log2 fold changes are only displayed for genes with adjusted p-value < 0.05 & Log2 Fold Change > |1|.

Gene Family	*M.paleacea* ID	MAPAL 5WPI L2FC	MAPAL 8WPI L2FC	MAPAL 11WPI L2FC	*M. truncatula* ID	MTR 8DPI L2FC	MTR 13DPI L2FC	MTR 27DPI L2FC	Homology
PP2A	IHWO_myc10130	n.s.	n.s.	n.s.	Medtr1g112940	n.s.	n.s.	n.s.	Ortholog
AP2A	Marpal_utg000001g0000321	n.s.	n.s.	n.s.	Medtr7g011630	n.s.	66.161	216.301	Ortholog
EPP1/HYP	Marpal_utg000002g0001491	n.s.	n.s.	n.s.	Medtr3g099200	n.s.	n.s.	n.s.	Ortholog
DXS2a	Marpal_utg000002g0002531	n.s.	n.s.	n.s.	Medtr8g068300	2.738	2.295	1.666	Ortholog
MtGA20ox	Marpal_utg000012g0030651	n.s.	1.579	5.949	Medtr1g102070	1.876	2.317	2.060	Proto-Ortholog
HA1	Marpal_utg000008g0017601	n.s.	n.s.	n.s.	Medtr8g006790	77.739	952.644	1552.447	Proto-Ortholog
CCD/ZAS	Marpal_utg000010g0020551	n.s.	n.s.	n.s.	Medtr3g110195	n.s.	n.s.	n.s.	Ortholog
RAD1	Marpal_utg000070g0119331	1.664	1.899	5.492	Medtr4g104020	39.881	237.890	696.850	Ortholog
MtGA20ox	Marpal_utg000011g0026241	n.s.	n.s.	n.s.	Medtr1g102070	1.876	2.317	2.060	Proto-Ortholog
NSP1	Marpal_utg000012g0028161	n.s.	n.s.	n.s.	Medtr8g020840	1.675	1.950	1.596	Ortholog
KIN5	Marpal_utg000012g0029631	n.s.	n.s.	n.s.	Medtr3g104900	5.008	51.093	150.145	Ortholog
MAX3/CCD7	Marpal_utg000013g0034951	n.s.	1.541	3.184	Medtr7g045370	n.s.	n.s.	n.s.	Ortholog
KO	Marpal_utg000013g0032051	n.s.	n.s.	n.s.	Medtr2g105360	1.899	1.998	1.637	Ortholog
MAX4/CCD8	Marpal_utg000010g0024761	n.s.	n.s.	2.696	Medtr3g109610	1.979	2.069	1.999	Ortholog
DUF538	Marpal_utg000056g0097771	n.s.	n.s.	7.112	Medtr2g091210	n.s.	n.s.	n.s.	Ortholog
STR2	Marpal_utg000110g0155891	n.s.	1.594	3.818	Medtr5g030910	7.247	204.843	364.363	Ortholog
ARK	Marpal_utg000090g0143091	n.s.	n.s.	3.360	Medtr7g116650	2.553	16.189	42.756	Proto-Ortholog
MtGA20ox	Marpal_utg000015g0038871	n.s.	n.s.	n.s.	Medtr1g102070	1.876	2.317	2.060	Proto-Ortholog
D14L/KAI2	Marpal_utg000015g0039791	n.s.	n.s.	n.s.	Medtr4g095310	n.s.	n.s.	-1.557	Ortholog
NOPE1	Marpal_utg000018g0051451	n.s.	n.s.	n.s.	Medtr3g093270	6.431	11.989	15.667	Ortholog
AMPa	Marpal_utg000021g0055731	n.s.	n.s.	n.s.	Medtr3g111900	6.859	8.364	6.834	Ortholog
KO	Marpal_utg000013g0032101	n.s.	2.059	3.739	Medtr2g105360	1.899	1.998	1.637	Ortholog
MAX1/CYP711A	Marpal_utg000021g0055761	n.s.	n.s.	n.s.	Medtr3g104560	n.s.	n.s.	n.s.	Ortholog
LEA	Marpal_utg000068g0113121	n.s.	n.s.	-18.501	Medtr2g088610	n.s.	n.s.	174.169	Ortholog
NUP133	Marpal_utg000023g0058101	n.s.	n.s.	n.s.	Medtr5g097260	n.s.	n.s.	n.s.	Ortholog
NUP85	Marpal_utg000023g0058261	n.s.	n.s.	n.s.	Medtr1g006690	n.s.	n.s.	n.s.	Ortholog
AMPb	Marpal_utg000005g0011521	n.s.	n.s.	2.858	Medtr2g098490	68.864	249.524	282.967	Ortholog
NENA	Marpal_utg000034g0069021	n.s.	n.s.	n.s.	Medtr6g072020	n.s.	n.s.	n.s.	Ortholog
MAX4/CCD8	Marpal_utg000036g0072591	n.s.	n.s.	n.s.	Medtr3g109610	1.979	2.069	1.999	Ortholog
LEA	Marpal_utg000038g0074401	n.s.	n.s.	n.s.	Medtr2g088610	n.s.	n.s.	174.169	Ortholog
MtGA20ox	Marpal_utg000039g0074971	n.s.	n.s.	n.s.	Medtr1g102070	1.876	2.317	2.060	Proto-Ortholog
AP2A	Marpal_utg000040g0076701	n.s.	n.s.	n.s.	Medtr7g011630	n.s.	66.161	216.301	Ortholog
CERBERUS	Marpal_utg000041g0079031	n.s.	n.s.	n.s.	Medtr1g090320	n.s.	n.s.	1.732	Proto-Ortholog
POLLUX/DMI1	Marpal_utg000046g0082721	n.s.	n.s.	n.s.	Medtr2g005870	n.s.	n.s.	n.s.	Proto-Ortholog
CASTOR	Marpal_utg000046g0082721	n.s.	n.s.	n.s.	Medtr7g117580	n.s.	n.s.	n.s.	Proto-Ortholog
STR	Marpal_utg000139g0174371	n.s.	n.s.	6.028	Medtr8g107450	3.192	33.331	70.275	Ortholog
NSP2	Marpal_utg000049g0085231	n.s.	n.s.	n.s.	Medtr3g072710	n.s.	1.775	1.639	Ortholog
LYK3/CERK	Marpal_utg000050g0087701	n.s.	n.s.	n.s.	Medtr5g086130	n.s.	n.s.	n.s.	Ortholog
SYMRK/DMI2	Marpal_utg000051g0090241	n.s.	n.s.	n.s.	Medtr5g030920	n.s.	n.s.	n.s.	Ortholog
CYCLOPS/IPD3	Marpal_utg000051g0091871	n.s.	n.s.	n.s.	Medtr5g026850	n.s.	n.s.	n.s.	Ortholog
AMT2	Marpal_utg000013g0032291	n.s.	5.999	8.907	Medtr7g115050	15.350	364.807	1042.993	Proto-Ortholog
HYP3	Marpal_utg000055g0096851	n.s.	n.s.	n.s.	Medtr4g104750	5.112	13.359	11.793	Ortholog
MAX1/CYP711A	Marpal_utg000021g0055741	n.s.	n.s.	2.607	Medtr3g104560	n.s.	n.s.	n.s.	Ortholog
MSBP1	Marpal_utg000062g0105641	n.s.	n.s.	n.s.	Medtr6g054890	n.s.	n.s.	n.s.	Ortholog
LYK10	Marpal_utg000156g0182581	n.s.	n.s.	1.791	Medtr5g033490	n.s.	2.143	3.097	Ortholog
AMPB	Marpal_utg000013g0034161	n.s.	n.s.	3.297	Medtr2g098490	68.864	249.524	282.967	Ortholog
EXO	Marpal_utg000068g0113951	n.s.	n.s.	n.s.	Medtr1g017910	1.998	13.061	17.991	Proto-Ortholog
PT4	Marpal_utg000069g0117021	n.s.	n.s.	n.s.	Medtr1g028600	1297.762	22666.226	12471.769	Proto-Ortholog
HYP2	Marpal_utg000070g0118111	n.s.	n.s.	n.s.	Medtr3g467150	2.453	3.993	4.864	Ortholog
KO	Marpal_utg000021g0055821	n.s.	n.s.	1.609	Medtr2g105360	1.899	1.998	1.637	Ortholog
SUNN	Marpal_utg000071g0119821	n.s.	n.s.	n.s.	Medtr4g070970	n.s.	n.s.	n.s.	Proto-Ortholog
DHY	Marpal_utg000167g0186021	n.s.	n.s.	4.887	Medtr4g097510	56.687	68.803	28.678	Ortholog
VPY	Marpal_utg000143g0177401	n.s.	n.s.	4.544	Medtr6g027840	6.193	6.681	5.570	Ortholog
SYP132	Marpal_utg000074g0124311	n.s.	n.s.	n.s.	Medtr2g088700	n.s.	n.s.	n.s.	Proto-Ortholog
CCD/ZAS	Marpal_utg000077g0127141	n.s.	n.s.	n.s.	Medtr3g110195	n.s.	n.s.	n.s.	Ortholog
MAX2/D3	Marpal_utg000086g0138511	n.s.	n.s.	n.s.	Medtr4g080020	n.s.	n.s.	n.s.	Ortholog
MCA8	Marpal_utg000088g0140181	n.s.	n.s.	n.s.	Medtr7g100110	n.s.	n.s.	n.s.	Ortholog
RFC	Marpal_utg000064g0107131	n.s.	n.s.	3.106	Medtr5g020810	4.755	63.890	175.447	Proto-Ortholog
DELLA1	Marpal_utg000095g0145961	n.s.	n.s.	n.s.	Medtr3g065980	n.s.	n.s.	n.s.	Ortholog
CCD/ZAS	Marpal_utg000099g0148701	n.s.	n.s.	n.s.	Medtr3g110195	n.s.	n.s.	n.s.	Ortholog
DELLA1	Marpal_utg000103g0151291	n.s.	n.s.	n.s.	Medtr3g065980	n.s.	n.s.	n.s.	Ortholog
CCD1	Marpal_utg000106g0154041	n.s.	n.s.	n.s.	Medtr8g037310	n.s.	n.s.	n.s.	Ortholog
RAM2	Marpal_utg000108g0154471	n.s.	n.s.	2.057	Medtr1g040500	3.968	39.325	71.857	Proto-Ortholog
AP2B/WRI5/ERF1	Marpal_utg000074g0124251	n.s.	n.s.	3.829	Medtr7g009410	37.232	512.595	731.632	Ortholog
D27	Marpal_utg000152g0180621	n.s.	n.s.	1.619	Medtr1g471050	2.303	2.487	2.136	Ortholog
SYP132	Marpal_utg000126g0168881	n.s.	n.s.	n.s.	Medtr2g088700	n.s.	n.s.	n.s.	Proto-Ortholog
ABCB20b	Marpal_utg000033g0068061	n.s.	n.s.	2.552	Medtr3g093430	n.s.	1.554	2.149	Ortholog
CCaMK/DMI3	Marpal_utg000137g0173321	n.s.	n.s.	n.s.	Medtr8g043970	n.s.	n.s.	n.s.	Ortholog
NOPE1	Marpal_utg000054g0094671	n.s.	n.s.	5.172	Medtr3g093270	6.431	11.989	15.667	Ortholog
ABCB20a	Marpal_utg000130g0170881	n.s.	n.s.	2.223	Medtr3g093430	n.s.	1.554	2.149	Ortholog
D14	Marpal_utg000147g0178301	n.s.	n.s.	n.s.	Medtr1g018320	n.s.	n.s.	n.s.	Proto-Ortholog
MAX4/CCD8	Marpal_utg000049g0083651	n.s.	n.s.	1.344	Medtr3g109610	1.979	2.069	1.999	Ortholog
CERBERUS	Marpal_utg000112g0157671	n.s.	n.s.	1.591	Medtr1g090320	n.s.	n.s.	1.732	Proto-Ortholog
RAD1	Marpal_utg000071g0120271	n.s.	n.s.	3.180	Medtr4g104020	39.881	237.890	696.850	Ortholog

Dataset S1. Genes differentially expressed in *M. paleacea* after 5, 8, or 11 weeks post inoculation with *R. irregularis*. Only genes with an adjusted p-value < 0.05 and Log2 Fold Change (L2FC) > |1| at at least one time point are shown.

Dataset S2. GO enrichment analysis of genes differentially expressed after 5, 8 or 11 weeks post inoculation with *R. irregularis,* reduced to most specific terms. Significantly enriched terms (adjusted P-value < 0.05 and at least 4 genes in the test set) were identified with a two-tailed Fisher's exact test with p-value correction according to Benjamini-Hochberg.

Dataset S3. Phylogenetic trees of 29 arbuscular mycorrhizal gene families that are conserved in angiosperms and liverwort host plants. Phylogenies were generated using genomic data from 65 plant and algal species. Clades conserved for AM symbiosis are labeled in purple. Dark blue = Angiosperm, Host; Light blue = Non-flowering plant, Host; Magenta = Angiosperm, Non-host; Pink = Non-flowering plant, Non-host. Names of gene families are included in each phylogeny.

SI References

1. A. Miyao, *et al.*, Target site specificity of the Tos17 retrotransposon shows a preference for insertion within genes and against insertion in retrotransposon-rich regions of the genome. *Plant Cell* (2003).
2. R. Roth, *et al.*, A rice Serine/Threonine receptor-like kinase regulates arbuscular mycorrhizal symbiosis at the peri-arbuscular membrane. *Nat Commun* (2018).
3. U. Paszkowski, T. Boller, The growth defect of lrt1, a maize mutant lacking lateral roots, can be complemented by symbiotic fungi or high phosphate nutrition. *Planta* (2002).
4. G. Tyler, P. A. Olsson, The calcifuge behaviour of Visearía vulgarís . *Journal of Vegetation Science* (1993).
5. G. Bécard, J. A. Fortin, Early events of vesicular–arbuscular mycorrhiza formation on Ri T-DNA transformed roots. *New Phytologist* **108**, 211–218 (1988).
6. U. Paszkowski, L. Jakovleva, T. Boller, Maize mutants affected at distinct stages of the arbuscular mycorrhizal symbiosis. *Plant Journal* (2006).
7. S. S. Shapiro, M. B. Wilk, An Analysis of Variance Test for Normality (Complete Samples). *Biometrika* (1965).
8. Student, The Probable Error of a Mean. *Biometrika* (1908).
9. H. B. Mann, D. R. Whitney, On a Test of Whether one of Two Random Variables is Stochastically Larger than the Other. *The Annals of Mathematical Statistics* (1947).
10. C. P. Humphreys, *et al.*, Mutualistic mycorrhiza-like symbiosis in the most ancient group of land plants. *Nat Commun* **1**, 103 (2010).
11. C. Gutjahr, *et al.*, Arbuscular mycorrhiza-specific signaling in rice transcends the common symbiosis signaling pathway. *Plant Cell* **20**, 2989–3005 (2008).
12. S. S. Sugano, *et al.*, Efficient CRISPR/Cas9-based genome editing and its application to conditional genetic analysis in Marchantia polymorpha. *PLoS One* (2018).
13. M. Kearse, *et al.*, Geneious Basic: An integrated and extendable desktop software platform for the organization and analysis of sequence data. *Bioinformatics* **28**, 1647–1649 (2012).
14. J. G. Doench, *et al.*, Rational design of highly active sgRNAs for CRISPR-Cas9–mediated gene inactivation. *Nat Biotechnol* **32**, 1262–1267 (2014).
15. S. Sauret-Güeto, *et al.*, Systematic Tools for Reprogramming Plant Gene Expression in a Simple Model, Marchantia polymorpha. *ACS Synth Biol* (2020).
16. A. Kubota, K. Ishizaki, M. Hosaka, T. Kohchi, Efficient Agrobacterium-mediated transformation of the liverwort Marchantia polymorpha using regenerating thalli. *Biosci Biotechnol Biochem* (2013).
17. S. Toki, *et al.*, Early infection of scutellum tissue with Agrobacterium allows high-speed transformation of rice. *The Plant Journal* **47**, 969–976 (2006).

18. C. Sallaud, *et al.*, Highly efficient production and characterization of T-DNA plants for rice (Oryza sativa L.) functional genomics. *Theoretical and Applied Genetics* **106**, 1396–1408 (2003).
19. K. Berendzen, *et al.*, A rapid and versatile combined DNA/RNA extraction protocol and its application to the analysis of a novel DNA marker set polymorphic between Arabidopsis thaliana ecotypes Col-0 and Landsberg erecta. *Plant Methods* **1**, 1–15 (2005).
20. F. Krueger, Trim Galore!: A wrapper around Cutadapt and FastQC to consistently apply adapter and quality trimming to FastQ files, with extra functionality for RRBS data. *Babraham Institute* (2015) (September 18, 2023).
21. M. K. Rich, *et al.*, Lipid exchanges drove the evolution of mutualism during plant terrestrialization. *Science* **372**, 864–868 (2021).
22. A. Dobin, *et al.*, STAR: ultrafast universal RNA-seq aligner. *Bioinformatics* **29**, 15 (2013).
23. Y. Liao, G. K. Smyth, W. Shi, featureCounts: an efficient general purpose program for assigning sequence reads to genomic features. *Bioinformatics* **30**, 923–930 (2014).
24. M. I. Love, W. Huber, S. Anders, Moderated estimation of fold change and dispersion for RNA-seq data with DESeq2. *Genome Biol* (2014).
25. BioBam Bioinformatics, OmicsBox - Bioinformatics made easy (2019).
26. F. Al-Shahrour, R. Díaz-Uriarte, J. Dopazo, FatiGO: A web tool for finding significant associations of Gene Ontology terms with groups of genes. *Bioinformatics* (2004).
27. L. H. Luginbuehl, *et al.*, Fatty acids in arbuscular mycorrhizal fungi are synthesized by the host plant. *Science* **356**, 1175–1178 (2017).
