# Supplementary material for: The receptor-like kinase ARK controls symbiotic balance across land plants
Source: Proc Natl Acad Sci U S A. 2024 Jul 16;121(30):e2318982121. doi: 10.1073/pnas.2318982121 (PMC11287157; doi:10.1073/pnas.2318982121)
Supplement: Supplementary file 4 — Dataset S03 (PDF) [file pnas.2318982121.sd03.pdf]

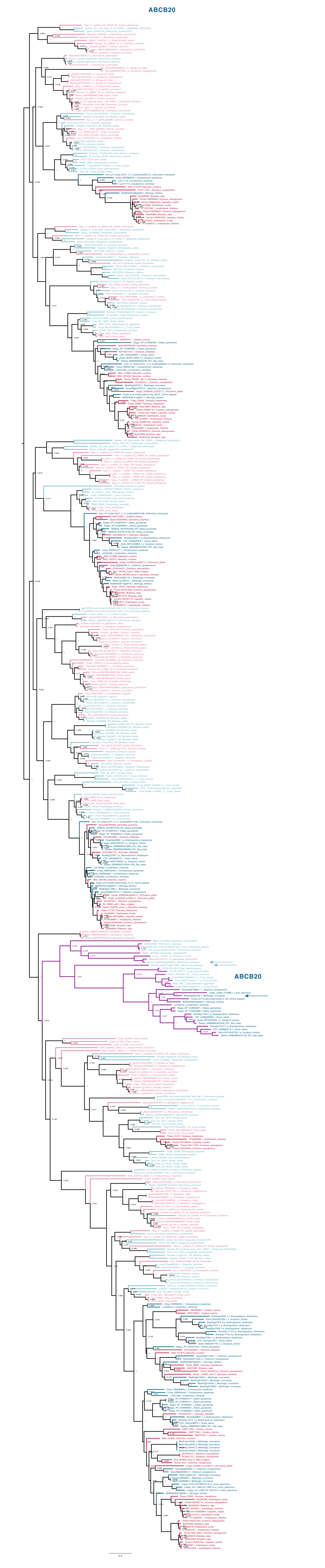
ABCB20
ABCB20

AMPa & AMPB

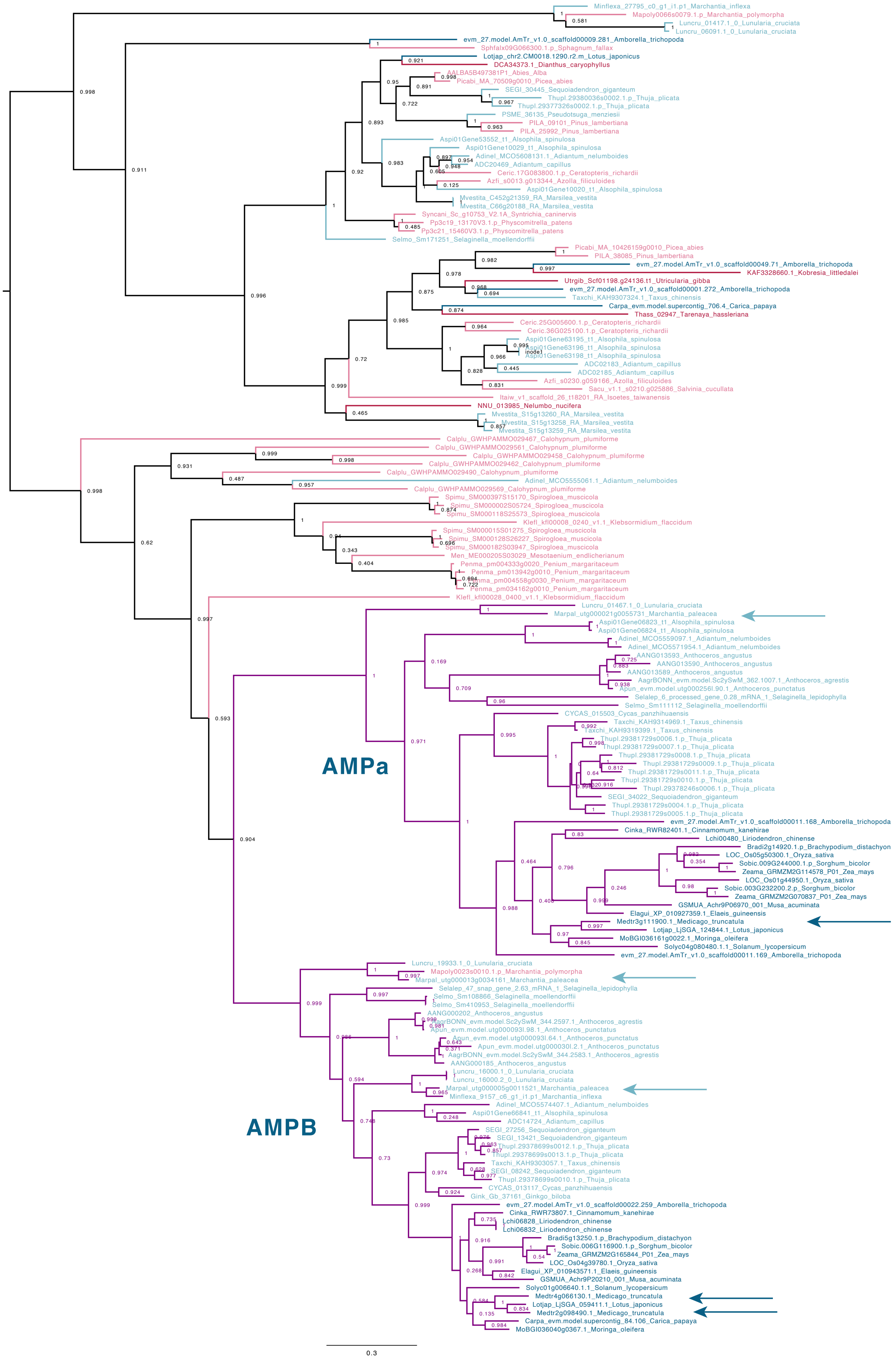

Minflexa_27795_c0_g1_i1.p1_Marchantia_inflexa
Mapoly0066s0079.1.p_Marchantia_polymorpha
Luncru_01417.1_0_Lunularia_cruciata
Luncru_06091.1_0_Lunularia_cruciata
evm_27.model.AmTr_v1.0_scaffold00009.281_Amborella_trichopoda
Sphfalx09G066300.1.p_Sphagnum_fallax
Lotjap_chr2.CM0018.1290.r2.m_Lotus_japonicus
DCA34373.1_Dianthus_caryophyllus
AALBA5B497381P1_Abies_Alba
Picabi_MA_70509g0010_Picea_abies
SEGI_30445_Sequoiadendron_giganteum
Thupl.29380036s0002.1.p_Thuja_plicata
Thupl.29377326s0002.1.p_Thuja_plicata
PSME_36135_Pseudotsuga_menziesii
PILA_09101_Pinus_lambertiana
PILA_25992_Pinus_lambertiana
Aspi01Gene53552_t1_Alsophila_spinulosa
Aspi01Gene10029_t1_Alsophila_spinulosa
Adinel_MCO5608131.1_Adiantum_nelumboides
ADC20469_Adiantum_capillus
Ceric.17G083800.1.p_Ceratopteris_richardii
Azfi_s0013.g013344_Azolla_filiculoides
Aspi01Gene10020_t1_Alsophila_spinulosa
Mvestita_C452g21359_RA_Marsilea_vestita
Mvestita_C66g20188_RA_Marsilea_vestita
Syncani_Sc_g10753_V2.1A_Syntrichia_caninervis
Pp3c19_13170V3.1.p_Physcomitrella_patens
Pp3c21_15460V3.1.p_Physcomitrella_patens
Selmo_Sm171251_Selaginella_moellendorffii
Picabi_MA_10426159g0010_Picea_abies
PILA_38085_Pinus_lambertiana
evm_27.model.AmTr_v1.0_scaffold00049.71_Amborella_trichopoda
KAF3328660.1_Kobresia_littledalei
Utrgib_Scf01198.g24136.t1_Utricularia_gibba
evm_27.model.AmTr_v1.0_scaffold00001.272_Amborella_trichopoda
Taxchi_KAH9307324.1_Taxus_chinensis
Carpa_evm.model.supercontig_706.4_Carica_papaya
Thass_02947_Tarenaya_hassleriana
Ceric.25G005600.1.p_Ceratopteris_richardii
Ceric.36G025100.1.p_Ceratopteris_richardii
Aspi01Gene63195_t1_Alsophila_spinulosa
Aspi01Gene63196_t1_Alsophila_spinulosa
Aspi01Gene63198_t1_Alsophila_spinulosa
ADC02183_Adiantum_capillus
ADC02185_Adiantum_capillus
Azfi_s0230.g059166_Azolla_filiculoides
Sacu_v1.1_s0210.g025886_Salvinia_cucullata
Itaiw_v1_scaffold_26_t18201_RA_Isoetes_taiwanensis
NNU_013985_Nelumbo_nucifera
Mvestita_S15g13260_RA_Marsilea_vestita
Mvestita_S15g13258_RA_Marsilea_vestita
Mvestita_S15g13259_RA_Marsilea_vestita
Calplu_GWHPAMMO029467_Calohypnum_plumiforme
Calplu_GWHPAMMO029561_Calohypnum_plumiforme
Calplu_GWHPAMMO029458_Calohypnum_plumiforme
Calplu_GWHPAMMO029462_Calohypnum_plumiforme
Calplu_GWHPAMMO029490_Calohypnum_plumiforme
Adinel_MCO5555061.1_Adiantum_nelumboides
Calplu_GWHPAMMO029569_Calohypnum_plumiforme
Spimu_SM000397S15170_Spirogloea_muscicola
Spimu_SM000002S05724_Spirogloea_muscicola
Spimu_SM000118S25573_Spirogloea_muscicola
Klefl_kfl00008_0240_v1.1_Klebsormidium_flaccidum
Spimu_SM000015S01275_Spirogloea_muscicola
Spimu_SM000128S26227_Spirogloea_muscicola
Spimu_SM000182S03947_Spirogloea_muscicola
Men_ME000205S03029_Mesotaenium_endlicherianum
Penma_pm004333g0020_Penium_margaritaceum
Penma_pm013942g0010_Penium_margaritaceum
Penma_pm004558g0030_Penium_margaritaceum
Penma_pm034162g0010_Penium_margaritaceum
Klefl_kfl00028_0400_v1.1_Klebsormidium_flaccidum
Luncru_01467.1_0_Lunularia_cruciata
Marpal_utg000021g0055731_Marchantia_paleacea
Aspi01Gene06823_t1_Alsophila_spinulosa
Aspi01Gene06824_t1_Alsophila_spinulosa
Adinel_MCO5559097.1_Adiantum_nelumboides
Adinel_MCO5571954.1_Adiantum_nelumboides
AANG013593_Anthoceros_angustus
AANG013590_Anthoceros_angustus
AANG013589_Anthoceros_angustus
AagrBONN_evm.model.Sc2ySwM_362.1007.1_Anthoceros_agrestis
Apun_evm.model.utg000256l.90.1_Anthoceros_punctatus
Selalep_6_processed_gene_0.28_mRNA_1_Selaginella_lepidophylla
Selmo_Sm111112_Selaginella_moellendorffii
CYCAS_015503_Cycas_panzhihuaensis
Taxchi_KAH9314969.1_Taxus_chinensis
Taxchi_KAH9319399.1_Taxus_chinensis
Thupl.29381729s0006.1.p_Thuja_plicata
Thupl.29381729s0007.1.p_Thuja_plicata
Thupl.29381729s0008.1.p_Thuja_plicata
Thupl.29381729s0009.1.p_Thuja_plicata
Thupl.29381729s0011.1.p_Thuja_plicata
Thupl.29381729s0010.1.p_Thuja_plicata
Thupl.29378246s0006.1.p_Thuja_plicata
SEGI_34022_Sequoiadendron_giganteum
Thupl.29381729s0004.1.p_Thuja_plicata
Thupl.29381729s0005.1.p_Thuja_plicata
AMPa
evm_27.model.AmTr_v1.0_scaffold00011.168_Amborella_trichopoda
Cinka_RWR82401.1_Cinnamomum_kanehirae
Lchi00480_Liriodendron_chinense
Bradi2g14920.1.p_Brachypodium_distachyon
LOC_Os05g50300.1_Oryza_sativa
Sobic.009G244000.1.p_Sorghum_bicolor
Zeama_GRMZM2G114578_P01_Zea_mays
LOC_Os01g44950.1_Oryza_sativa
Sobic.003G232200.2.p_Sorghum_bicolor
Zeama_GRMZM2G070837_P01_Zea_mays
GSMUA_Achr9P06970_001_Musa_acuminata
Elagui_XP_010927359.1_Elaeis_guineensis
Medtr3g111900.1_Medicago_truncatula
Lotjap_LjSGA_124844.1_Lotus_japonicus
MoBGI036161g0022.1_Moringa_oleifera
Solyc04g080480.1.1_Solanum_lycopersicum
evm_27.model.AmTr_v1.0_scaffold00011.169_Amborella_trichopoda
Luncru_19933.1_0_Lunularia_cruciata
Mapoly0023s0010.1.p_Marchantia_polymorpha
Marpal_utg000013g0034161_Marchantia_paleacea
Selalep_47_snap_gene_2.63_mRNA_1_Selaginella_lepidophylla
Selmo_Sm108866_Selaginella_moellendorffii
Selmo_Sm410953_Selaginella_moellendorffii
AANG000202_Anthoceros_angustus
AagrBONN_evm.model.Sc2ySwM_344.2597.1_Anthoceros_agrestis
Apun_evm.model.utg000093l.98.1_Anthoceros_punctatus
Apun_evm.model.utg000093l.64.1_Anthoceros_punctatus
Apun_evm.model.utg000030l.2.1_Anthoceros_punctatus
AagrBONN_evm.model.Sc2ySwM_344.2583.1_Anthoceros_agrestis
AANG000185_Anthoceros_angustus
Luncru_16000.1_0_Lunularia_cruciata
Luncru_16000.2_0_Lunularia_cruciata
Marpal_utg000005g0011521_Marchantia_paleacea
Minflexa_9157_c6_g1_i1.p1_Marchantia_inflexa
Adinel_MCO5574407.1_Adiantum_nelumboides
Aspi01Gene66841_t1_Alsophila_spinulosa
ADC14724_Adiantum_capillus
AMPB
SEGI_27256_Sequoiadendron_giganteum
SEGI_13421_Sequoiadendron_giganteum
Thupl.29378699s0012.1.p_Thuja_plicata
Thupl.29378699s0013.1.p_Thuja_plicata
Taxchi_KAH9303057.1_Taxus_chinensis
SEGI_08242_Sequoiadendron_giganteum
Thupl.29378699s0010.1.p_Thuja_plicata
CYCAS_013117_Cycas_panzhihuaensis
Gink_Gb_37161_Ginkgo_biloba
evm_27.model.AmTr_v1.0_scaffold00022.259_Amborella_trichopoda
Cinka_RWR73807.1_Cinnamomum_kanehirae
Lchi06828_Liriodendron_chinense
Lchi06832_Liriodendron_chinense
Bradi5g13250.1.p_Brachypodium_distachyon
Sobic.006G116900.1.p_Sorghum_bicolor
Zeama_GRMZM2G165844_P01_Zea_mays
LOC_Os04g39780.1_Oryza_sativa
Elagui_XP_010943571.1_Elaeis_guineensis
GSMUA_Achr9P20210_001_Musa_acuminata
Solyc01g006640.1.1_Solanum_lycopersicum
Medtr4g066130.1_Medicago_truncatula
Lotjap_LjSGA_059411.1_Lotus_japonicus
Medtr2g098490.1_Medicago_truncatula
Carpa_evm.model.supercontig_84.106_Carica_papaya
MoBGI036040g0367.1_Moringa_oleifera
0.3

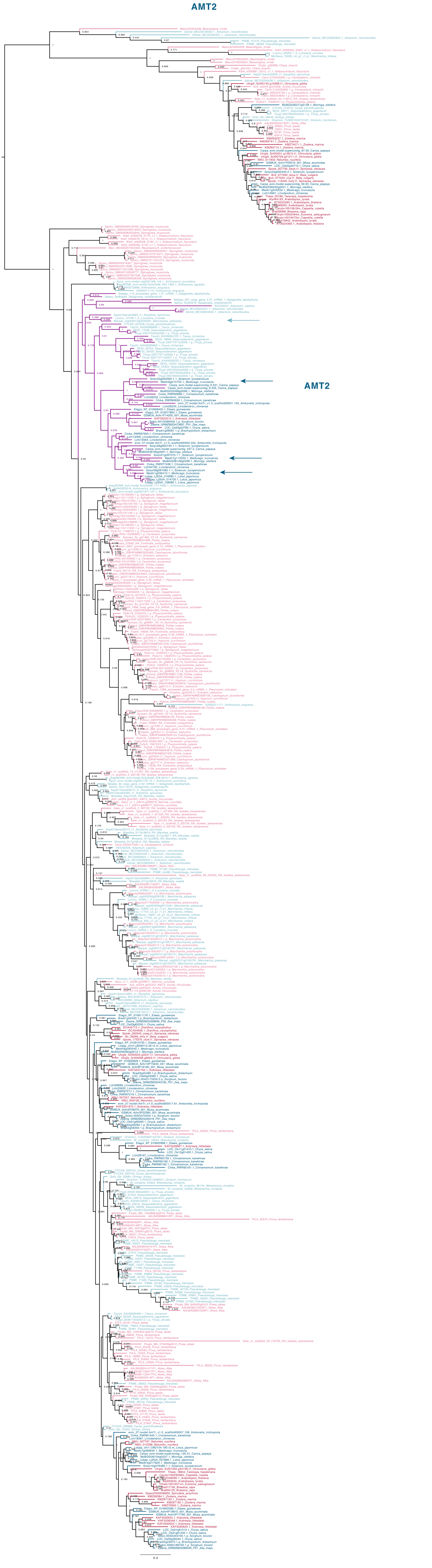
AMT2
AMT2
0.3

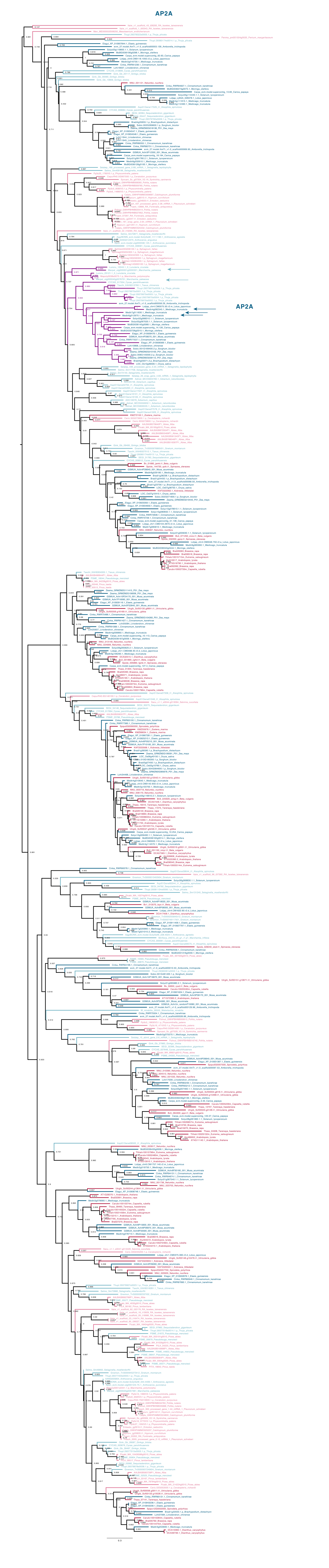
AP2A
AP2A

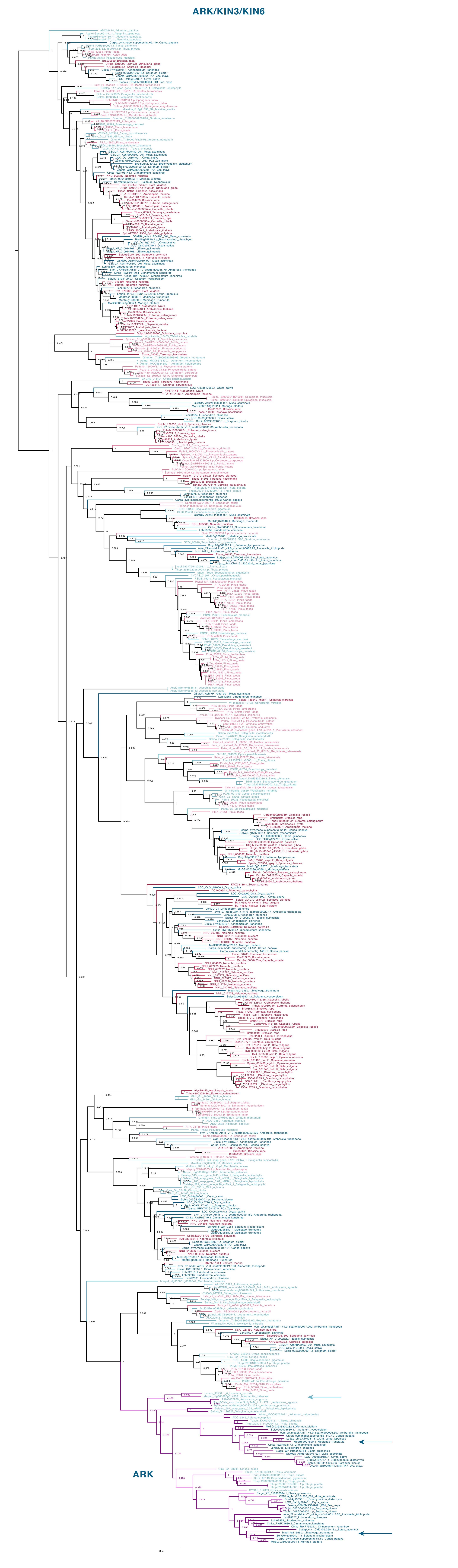
ARK/KIN3/KIN6
ARK
0.4

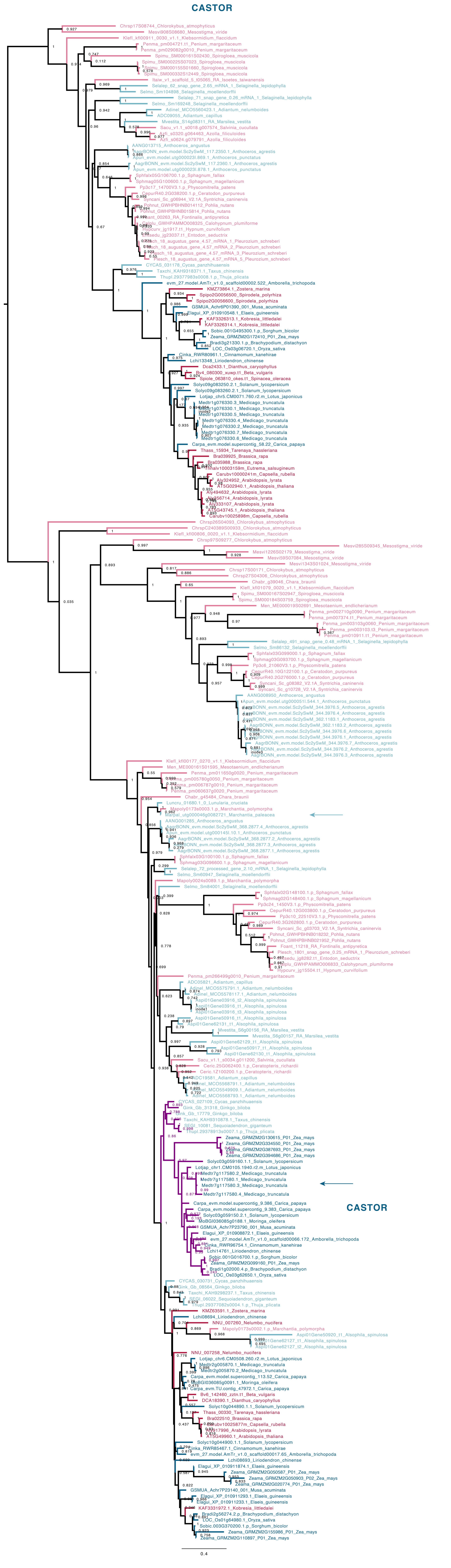
CASTOR
CASTOR
0.4

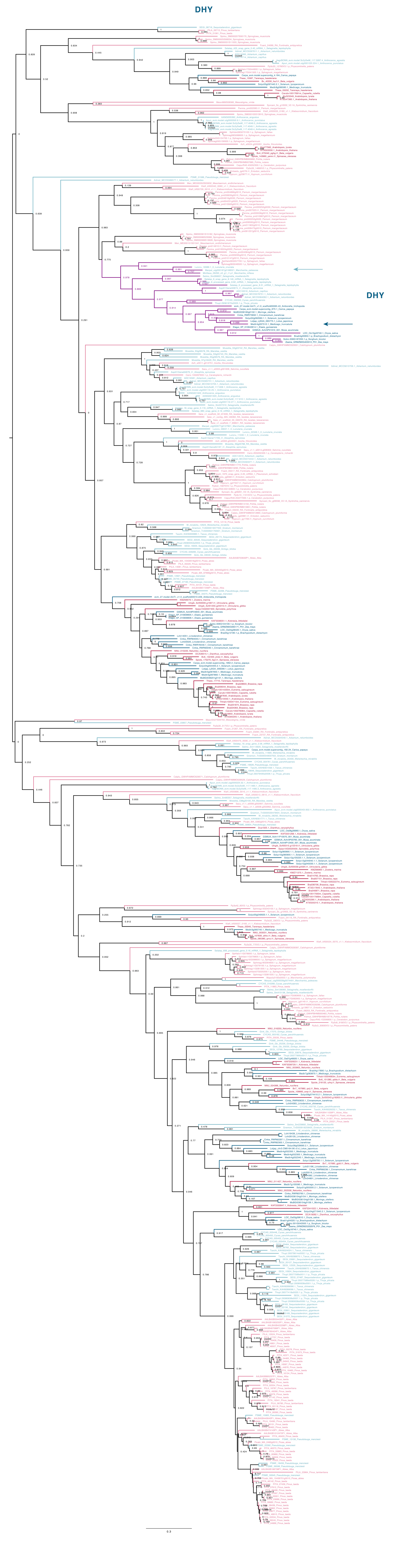
DHY
DHY
0.3

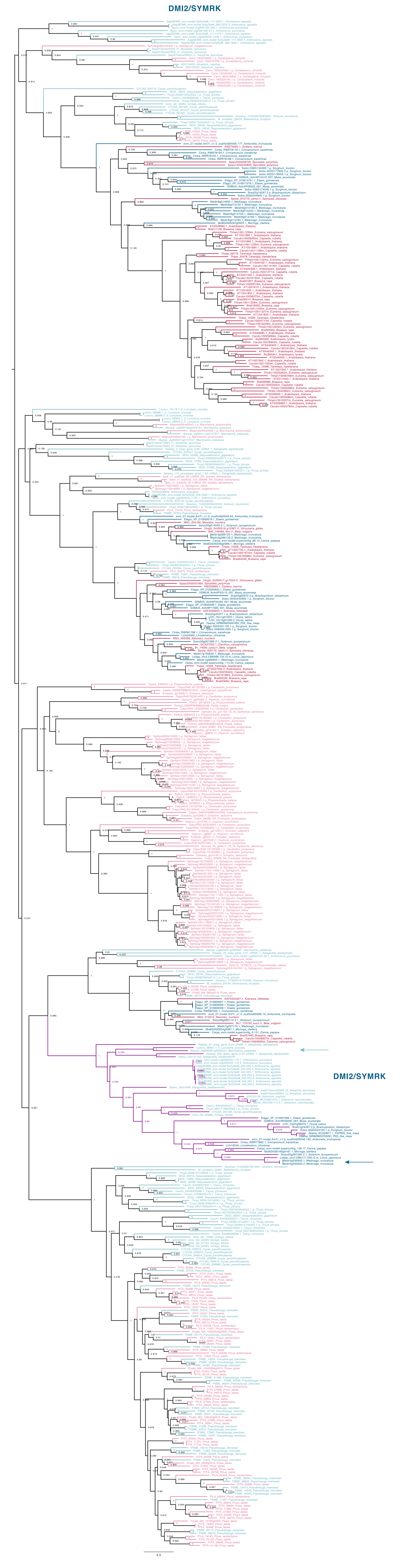
DMI2/SYMRK
DMI2/SYMRK
0.2

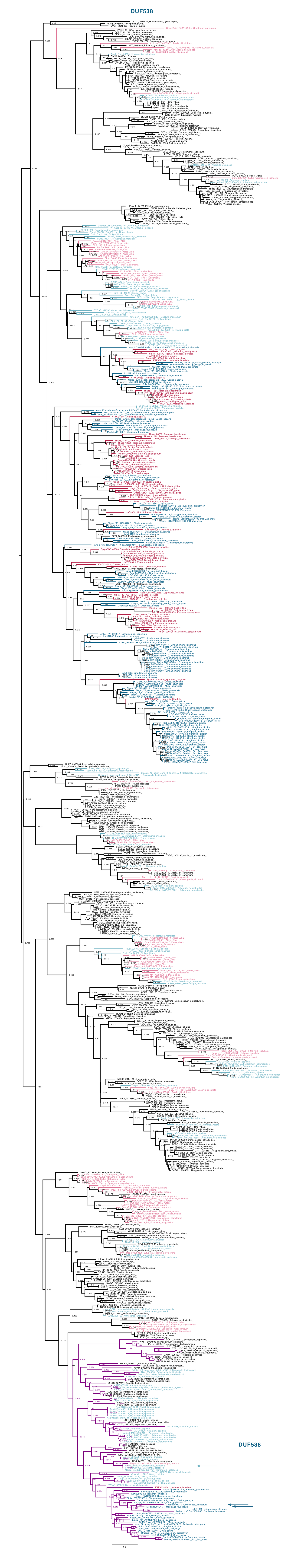
DUF538
DUF538
0.5

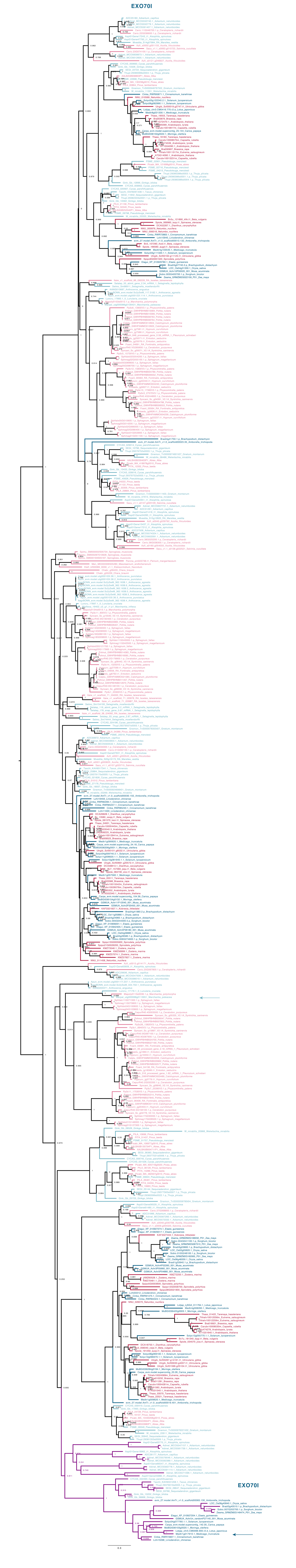
EXO70I
EXO70I
0.5

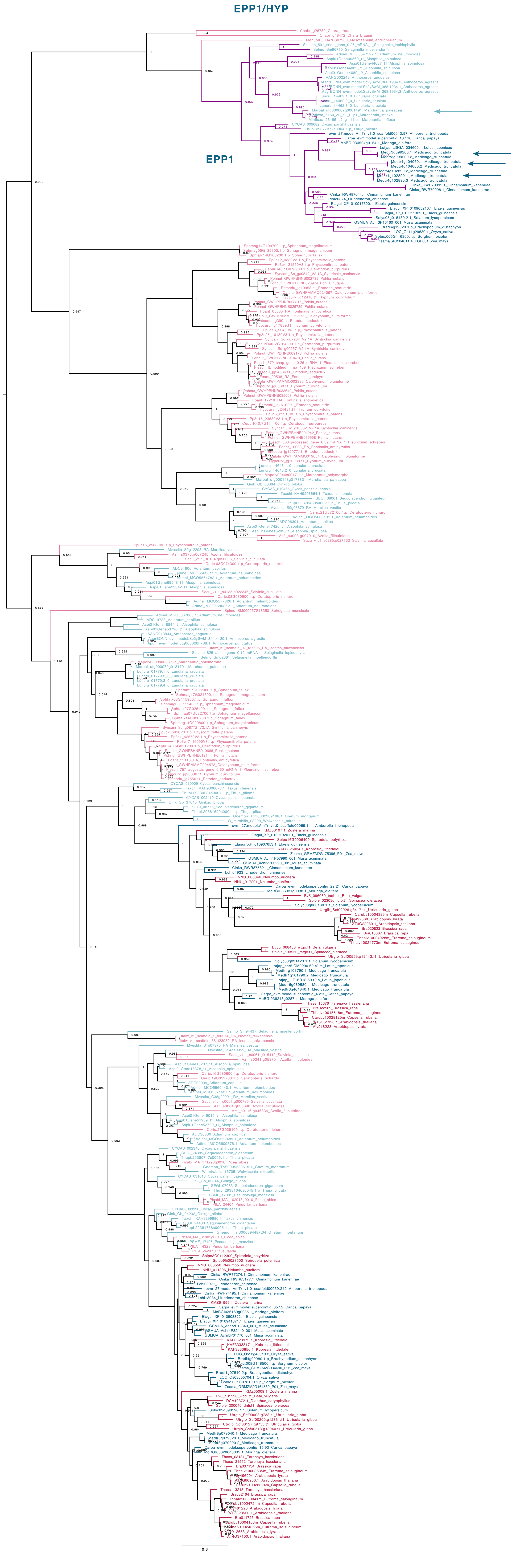

EPP1/HYP
EPP1
0.3

HYP2

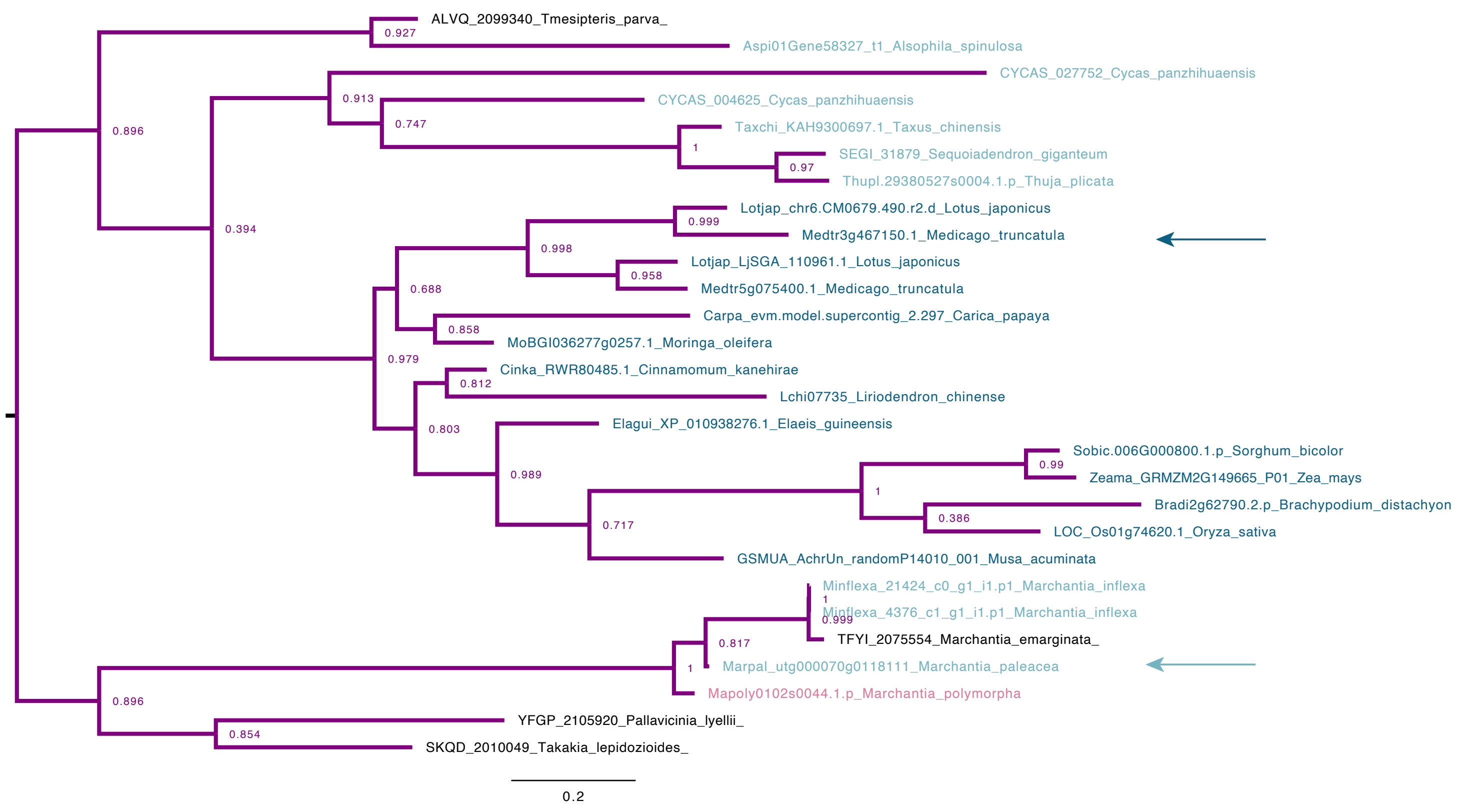

ALVQ_2099340_Tmesipteris_parva_
0.927
Aspi01Gene58327_t1_Alsophila_spinulosa
0.896
CYCAS_027752_Cycas_panzhihuaensis
0.913
CYCAS_004625_Cycas_panzhihuaensis
0.747
Taxchi_KAH9300697.1_Taxus_chinensis
1
SEGI_31879_Sequoiadendron_giganteum
0.97
Thupl.29380527s0004.1.p_Thuja_plicata
0.394
Lotjap_chr6.CM0679.490.r2.d_Lotus_japonicus
0.999
Medtr3g467150.1_Medicago_truncatula
0.998
Lotjap_LjSGA_110961.1_Lotus_japonicus
0.958
Medtr5g075400.1_Medicago_truncatula
0.688
Carpa_evm.model.supercontig_2.297_Carica_papaya
0.858
MoBGI036277g0257.1_Moringa_oleifera
0.979
Cinka_RWR80485.1_Cinnamomum_kanehirae
0.812
Lchi07735_Liriodendron_chinense
0.803
Elagui_XP_010938276.1_Elaeis_guineensis
0.989
Sobic.006G000800.1.p_Sorghum_bicolor
0.99
Zeama_GRMZM2G149665_P01_Zea_mays
1
Bradi2g62790.2.p_Brachypodium_distachyon
0.386
LOC_Os01g74620.1_Oryza_sativa
0.717
GSMUA_AchrUn_randomP14010_001_Musa_acuminata
Minflexa_21424_c0_g1_i1.p1_Marchantia_inflexa
1
Minflexa_4376_c1_g1_i1.p1_Marchantia_inflexa
0.999
TFYI_2075554_Marchantia_emarginata_
0.817
Marpal_utg000070g0118111_Marchantia_paleacea
1
Mapoly0102s0044.1.p_Marchantia_polymorpha
0.896
YFGP_2105920_Pallavicinia_lyellii_
0.854
SKQD_2010049_Takakia_lepidozioides_
0.2

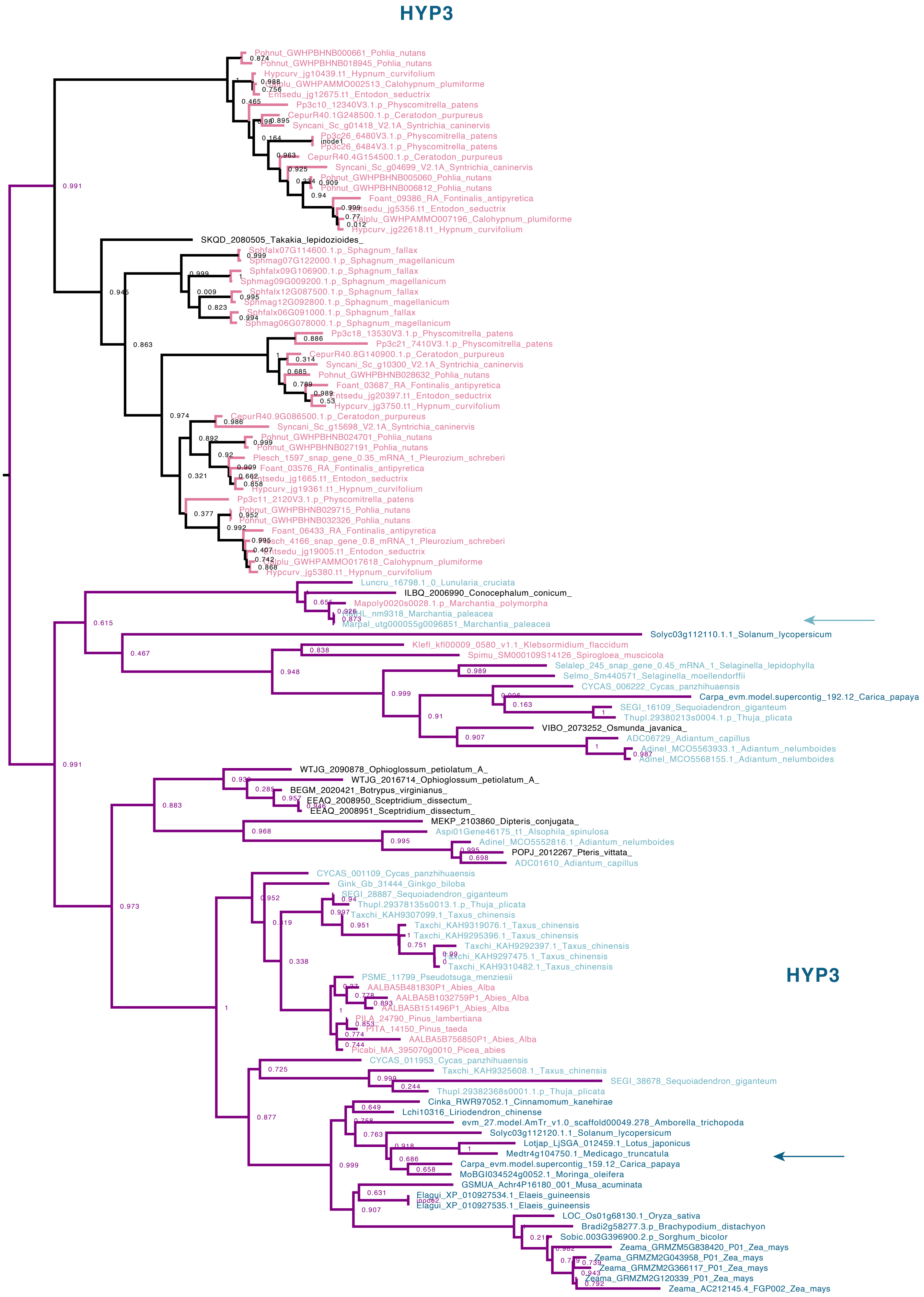

HYP3
Pohnut_GWHPBHNB000661_Pohlia_nutans
Pohnut_GWHPBHNB018945_Pohlia_nutans
Hypcurv_jg10439.t1_Hypnum_curvifolium
Calplu_GWHPAMMO002513_Calohypnum_plumiforme
Entsedu_jg12675.t1_Entodon_seductrix
Pp3c10_12340V3.1.p_Physcomitrella_patens
CepurR40.1G248500.1.p_Ceratodon_purpureus
Syncani_Sc_g01418_V2.1A_Syntrichia_caninervis
Pp3c26_6480V3.1.p_Physcomitrella_patens
Pp3c26_6484V3.1.p_Physcomitrella_patens
CepurR40.4G154500.1.p_Ceratodon_purpureus
Syncani_Sc_g04699_V2.1A_Syntrichia_caninervis
Pohnut_GWHPBHNB005060_Pohlia_nutans
Pohnut_GWHPBHNB006812_Pohlia_nutans
Foant_09386_RA_Fontinalis_antipyretica
Entsedu_jg5356.t1_Entodon_seductrix
Calplu_GWHPAMMO007196_Calohypnum_plumiforme
Hypcurv_jg22618.t1_Hypnum_curvifolium
SKQD_2080505_Takakia_lepidozioides_
Sphfalx07G114600.1.p_Sphagnum_fallax
Sphmag07G122000.1.p_Sphagnum_magellanicum
Sphfalx09G106900.1.p_Sphagnum_fallax
Sphmag09G009200.1.p_Sphagnum_magellanicum
Sphfalx12G087500.1.p_Sphagnum_fallax
Sphmag12G092800.1.p_Sphagnum_magellanicum
Sphfalx06G091000.1.p_Sphagnum_fallax
Sphmag06G078000.1.p_Sphagnum_magellanicum
Pp3c18_13530V3.1.p_Physcomitrella_patens
Pp3c21_7410V3.1.p_Physcomitrella_patens
CepurR40.8G140900.1.p_Ceratodon_purpureus
Syncani_Sc_g10300_V2.1A_Syntrichia_caninervis
Pohnut_GWHPBHNB028632_Pohlia_nutans
Foant_03687_RA_Fontinalis_antipyretica
Entsedu_jg20397.t1_Entodon_seductrix
Hypcurv_jg3750.t1_Hypnum_curvifolium
CepurR40.9G086500.1.p_Ceratodon_purpureus
Syncani_Sc_g15698_V2.1A_Syntrichia_caninervis
Pohnut_GWHPBHNB024701_Pohlia_nutans
Pohnut_GWHPBHNB027191_Pohlia_nutans
Plesch_1597_snap_gene_0.35_mRNA_1_Pleurozium_schreberi
Foant_03576_RA_Fontinalis_antipyretica
Entsedu_jg1665.t1_Entodon_seductrix
Hypcurv_jg19361.t1_Hypnum_curvifolium
Pp3c11_2120V3.1.p_Physcomitrella_patens
Pohnut_GWHPBHNB029715_Pohlia_nutans
Pohnut_GWHPBHNB032326_Pohlia_nutans
Foant_06433_RA_Fontinalis_antipyretica
Plesch_4166_snap_gene_0.8_mRNA_1_Pleurozium_schreberi
Entsedu_jg19005.t1_Entodon_seductrix
Calplu_GWHPAMMO017618_Calohypnum_plumiforme
Hypcurv_jg5380.t1_Hypnum_curvifolium
Luncru_16798.1_0_Lunularia_cruciata
ILBQ_2006990_Conocephalum_conicum_
Mapoly0020s0028.1.p_Marchantia_polymorpha
TFWHL_nm9318_Marchantia_paleacea
Marpal_utg000055g0096851_Marchantia_paleacea
Solyc03g112110.1.1_Solanum_lycopersicum
Klefl_kfl00009_0580_v1.1_Klebsormidium_flaccidum
Spimu_SM000109S14126_Spirogloea_muscicola
Selalep_245_snap_gene_0.45_mRNA_1_Selaginella_lepidophylla
Selmo_Sm440571_Selaginella_moellendorffii
CYCAS_006222_Cycas_panzhihuaensis
Carpa_evm.model.supercontig_192.12_Carica_papaya
SEGI_16109_Sequoiadendron_giganteum
Thupl.29380213s0004.1.p_Thuja_plicata
VIBO_2073252_Osmunda_javanica_
ADC06729_Adiantum_capillus
Adinel_MCO5563933.1_Adiantum_nelumboides
Adinel_MCO5568155.1_Adiantum_nelumboides
WTJG_2090878_Ophioglossum_petiolatum_A_
WTJG_2016714_Ophioglossum_petiolatum_A_
BEGM_2020421_Botrypus_virginianus_
EEAQ_2008950_Sceptridium_dissectum_
EEAQ_2008951_Sceptridium_dissectum_
MEKP_2103860_Dipteris_conjugata_
Aspi01Gene46175_t1_Alsophila_spinulosa
Adinel_MCO5552816.1_Adiantum_nelumboides
POPJ_2012267_Pteris_vittata_
ADC01610_Adiantum_capillus
CYCAS_001109_Cycas_panzhihuaensis
Gink_Gb_31444_Ginkgo_biloba
SEGI_28887_Sequoiadendron_giganteum
Thupl.29378135s0013.1.p_Thuja_plicata
Taxchi_KAH9307099.1_Taxus_chinensis
Taxchi_KAH9319076.1_Taxus_chinensis
Taxchi_KAH9295396.1_Taxus_chinensis
Taxchi_KAH9292397.1_Taxus_chinensis
Taxchi_KAH9297475.1_Taxus_chinensis
Taxchi_KAH9310482.1_Taxus_chinensis
PSME_11799_Pseudotsuga_menziesii
AALBA5B481830P1_Abies_Alba
AALBA5B1032759P1_Abies_Alba
AALBA5B151496P1_Abies_Alba
PILA_24790_Pinus_lambertiana
PITA_14150_Pinus_taeda
AALBA5B756850P1_Abies_Alba
Picabi_MA_395070g0010_Picea_abies
CYCAS_011953_Cycas_panzhihuaensis
Taxchi_KAH9325608.1_Taxus_chinensis
SEGI_38678_Sequoiadendron_giganteum
Thupl.29382368s0001.1.p_Thuja_plicata
Cinka_RWR97052.1_Cinnamomum_kanehirae
Lchi10316_Liriodendron_chinense
evm_27.model.AmTr_v1.0_scaffold00049.278_Amborella_trichopoda
Solyc03g112120.1.1_Solanum_lycopersicum
Lotjap_LjSGA_012459.1_Lotus_japonicus
Medtr4g104750.1_Medicago_truncatula
Carpa_evm.model.supercontig_159.12_Carica_papaya
MoBGI034524g0052.1_Moringa_oleifera
GSMUA_Achr4P16180_001_Musa_acuminata
Elagui_XP_010927534.1_Elaeis_guineensis
Elagui_XP_010927535.1_Elaeis_guineensis
LOC_Os01g68130.1_Oryza_sativa
Bradi2g58277.3.p_Brachypodium_distachyon
Sobic.003G396900.2.p_Sorghum_bicolor
Zeama_GRMZM5G838420_P01_Zea_mays
Zeama_GRMZM2G043958_P01_Zea_mays
Zeama_GRMZM2G366117_P01_Zea_mays
Zeama_GRMZM2G120339_P01_Zea_mays
Zeama_AC212145.4_FGP002_Zea_mays
HYP3

CYCLOPS/IPD3

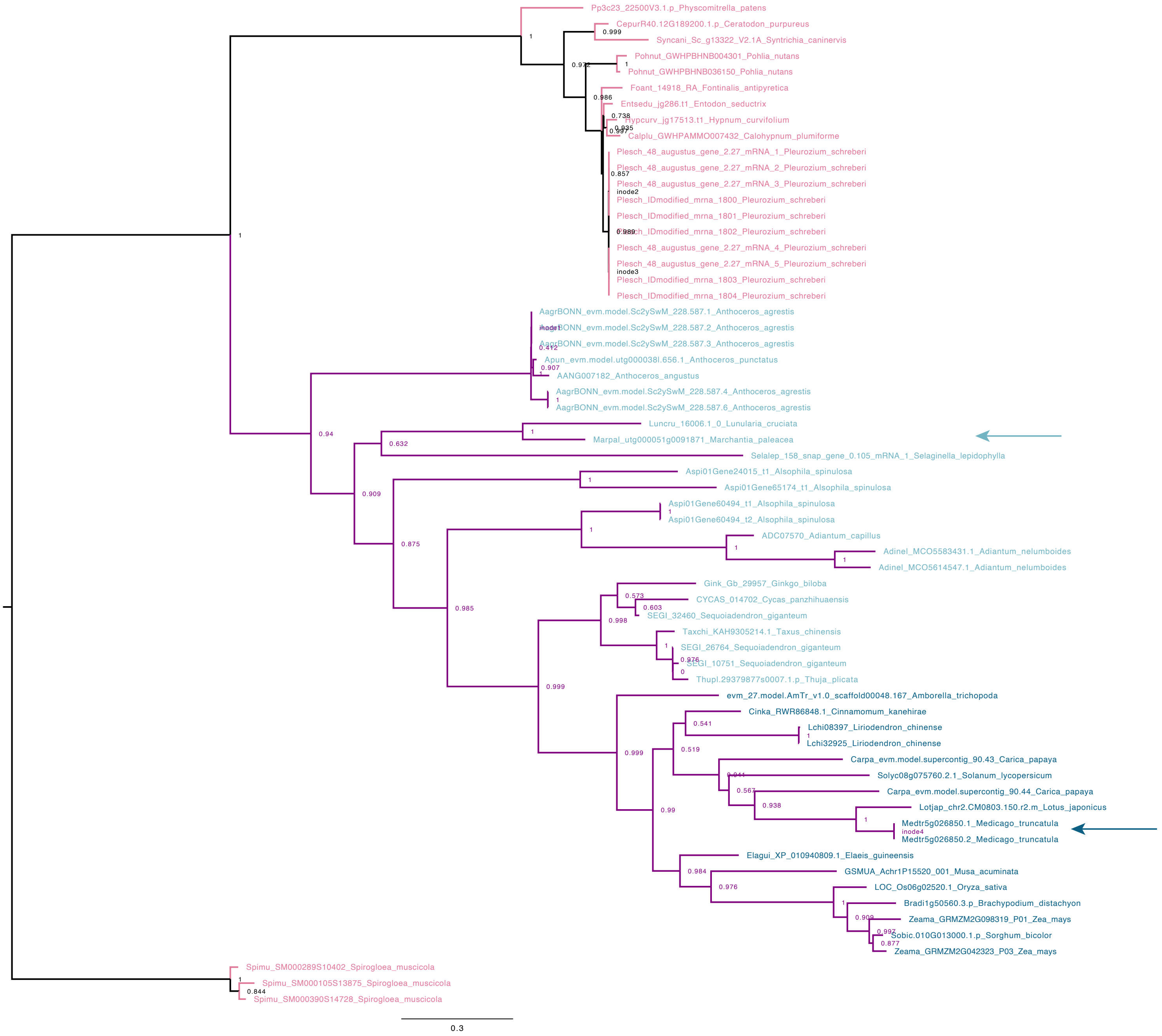

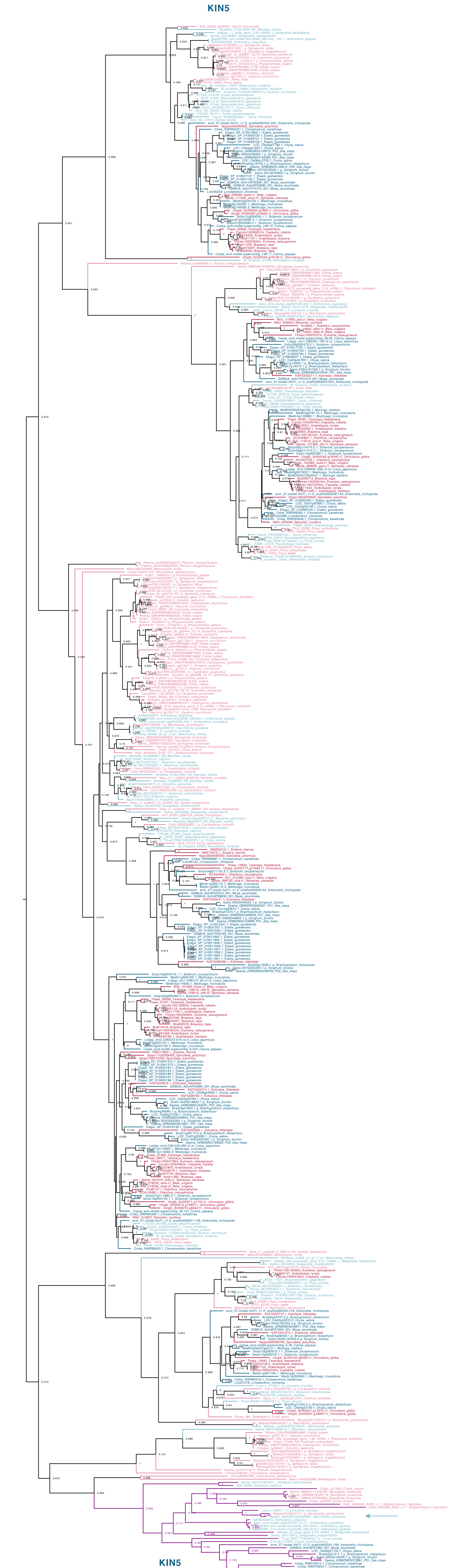
KIN5
KIN5

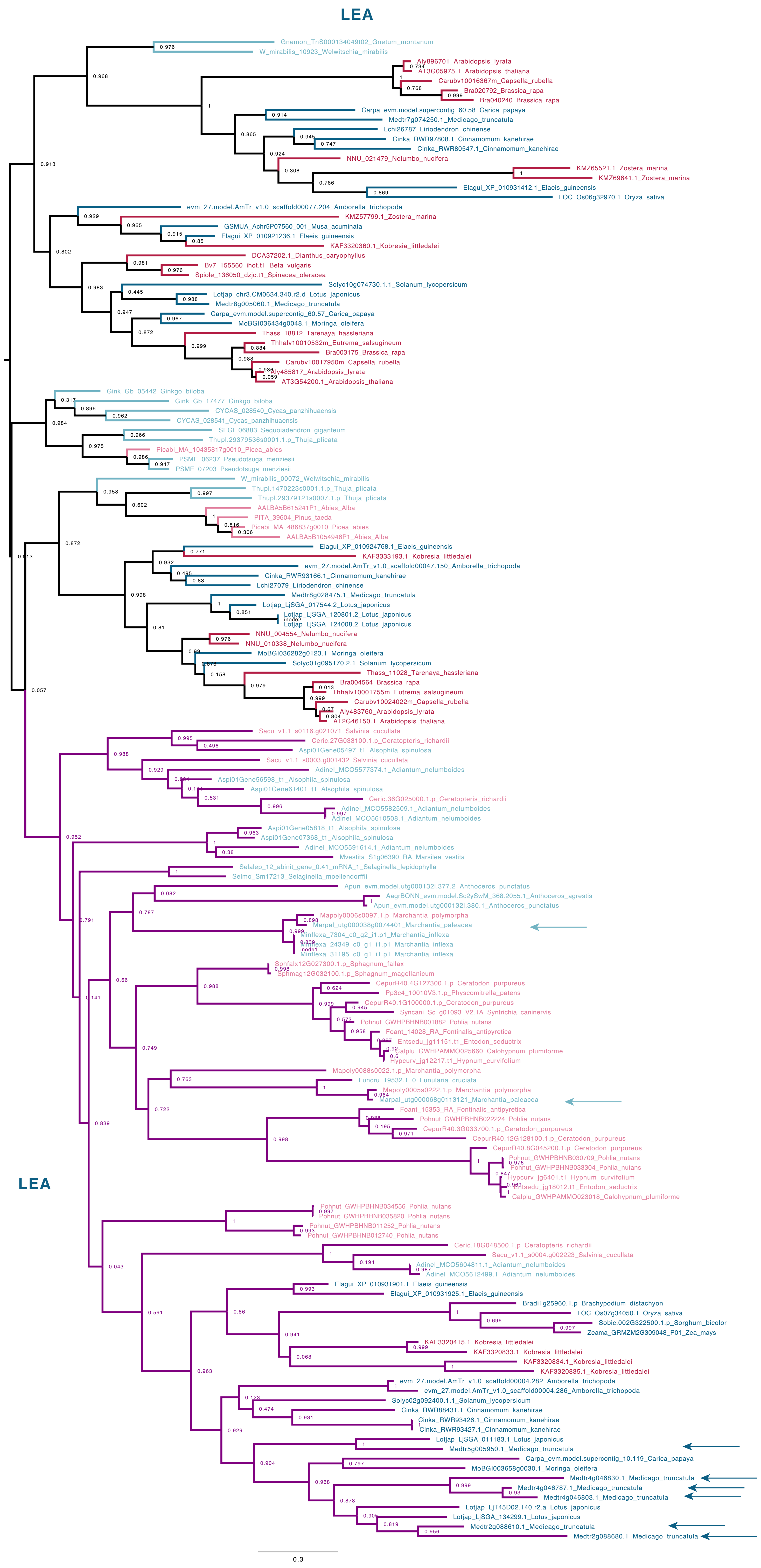
LEA
Gnemon_TnS000134049t02_Gnetum_montanum
W_mirabilis_10923_Welwitschia_mirabilis
Aly896701_Arabidopsis_lyrata
AT3G05975.1_Arabidopsis_thaliana
Carubv10016367m_Capsella_rubella
Bra020792_Brassica_rapa
Bra040240_Brassica_rapa
Carpa_evm.model.supercontig_60.58_Carica_papaya
Medtr7g074250.1_Medicago_truncatula
Lchi26787_Liriodendron_chinense
Cinka_RWR97808.1_Cinnamomum_kanehirae
Cinka_RWR80547.1_Cinnamomum_kanehirae
NNU_021479_Nelumbo_nucifera
KMZ65521.1_Zostera_marina
KMZ69641.1_Zostera_marina
Elagui_XP_010931412.1_Elaeis_guineensis
LOC_Os06g32970.1_Oryza_sativa
evm_27.model.AmTr_v1.0_scaffold00077.204_Amborella_trichopoda
KMZ57799.1_Zostera_marina
GSMUA_Achr5P07560_001_Musa_acuminata
Elagui_XP_010921236.1_Elaeis_guineensis
KAF3320360.1_Kobresia_littledalei
DCA37202.1_Dianthus_caryophyllus
Bv7_155560_ihot.t1_Beta_vulgaris
Spiole_136050_dzjc.t1_Spinacea_oleracea
Solyc10g074730.1.1_Solanum_lycopersicum
Lotjap_chr3.CM0634.340.r2.d_Lotus_japonicus
Medtr8g005060.1_Medicago_truncatula
Carpa_evm.model.supercontig_60.57_Carica_papaya
MoBGI036434g0048.1_Moringa_oleifera
Thass_18812_Tarenaya_hassleriana
Thhalv10010532m_Eutrema_salsugineum
Bra003175_Brassica_rapa
Carubv10017950m_Capsella_rubella
Aly485817_Arabidopsis_lyrata
AT3G54200.1_Arabidopsis_thaliana
Gink_Gb_05442_Ginkgo_biloba
Gink_Gb_17477_Ginkgo_biloba
CYCAS_028540_Cycas_panzhihuaensis
CYCAS_028541_Cycas_panzhihuaensis
SEGI_06883_Sequoiadendron_giganteum
Thupl.29379536s0001.1.p_Thuja_plicata
Picabi_MA_10435817g0010_Picea_abies
PSME_06237_Pseudotsuga_menziesii
PSME_07203_Pseudotsuga_menziesii
W_mirabilis_00072_Welwitschia_mirabilis
Thupl.1470223s0001.1.p_Thuja_plicata
Thupl.29379121s0007.1.p_Thuja_plicata
AALBA5B615241P1_Abies_Alba
PITA_39604_Pinus_taeda
Picabi_MA_486837g0010_Picea_abies
AALBA5B1054946P1_Abies_Alba
Elagui_XP_010924768.1_Elaeis_guineensis
KAF3333193.1_Kobresia_littledalei
evm_27.model.AmTr_v1.0_scaffold00047.150_Amborella_trichopoda
Cinka_RWR93166.1_Cinnamomum_kanehirae
Lchi27079_Liriodendron_chinense
Medtr8g028475.1_Medicago_truncatula
Lotjap_LjSGA_017544.2_Lotus_japonicus
Lotjap_LjSGA_120801.2_Lotus_japonicus
Lotjap_LjSGA_124008.2_Lotus_japonicus
NNU_004554_Nelumbo_nucifera
NNU_010338_Nelumbo_nucifera
MoBGI036282g0123.1_Moringa_oleifera
Solyc01g095170.2.1_Solanum_lycopersicum
Thass_11028_Tarenaya_hassleriana
Bra004564_Brassica_rapa
Thhalv10001755m_Eutrema_salsugineum
Carubv10024022m_Capsella_rubella
Aly483760_Arabidopsis_lyrata
AT2G46150.1_Arabidopsis_thaliana
Sacu_v1.1_s0116.g021071_Salvinia_cucullata
Ceric.27G033100.1.p_Ceratopteris_richardii
Aspi01Gene05497_t1_Alsophila_spinulosa
Sacu_v1.1_s0003.g001432_Salvinia_cucullata
Adinel_MCO5577374.1_Adiantum_nelumboides
Aspi01Gene56598_t1_Alsophila_spinulosa
Aspi01Gene61401_t1_Alsophila_spinulosa
Ceric.36G025000.1.p_Ceratopteris_richardii
Adinel_MCO5582509.1_Adiantum_nelumboides
Adinel_MCO5610508.1_Adiantum_nelumboides
Aspi01Gene05818_t1_Alsophila_spinulosa
Aspi01Gene07368_t1_Alsophila_spinulosa
Adinel_MCO5591614.1_Adiantum_nelumboides
Mvestita_S1g06390_RA_Marsilea_vestita
Selalep_12_abinit_gene_0.41_mRNA_1_Selaginella_lepidophylla
Selmo_Sm17213_Selaginella_moellendorffii
Apun_evm.model.utg000132l.377.2_Anthoceros_punctatus
AagrBONN_evm.model.Sc2ySwM_368.2055.1_Anthoceros_agrestis
Apun_evm.model.utg000132l.380.1_Anthoceros_punctatus
Mapoly0006s0097.1.p_Marchantia_polymorpha
Marpal_utg000038g0074401_Marchantia_paleacea
Minflexa_7304_c0_g2_i1.p1_Marchantia_inflexa
Minflexa_24349_c0_g1_i1.p1_Marchantia_inflexa
Minflexa_31195_c0_g1_i1.p1_Marchantia_inflexa
Sphfalx12G027300.1.p_Sphagnum_fallax
Sphmag12G032100.1.p_Sphagnum_magellanicum
CepurR40.4G127300.1.p_Ceratodon_purpureus
Pp3c4_10010V3.1.p_Physcomitrella_patens
CepurR40.1G100000.1.p_Ceratodon_purpureus
Syncani_Sc_g01093_V2.1A_Syntrichia_caninervis
Pohnut_GWHPBHNB001882_Pohlia_nutans
Foant_14028_RA_Fontinalis_antipyretica
Entsedu_jg11151.t1_Entodon_seductrix
Calplu_GWHPAMMO025660_Calohypnum_plumiforme
Hypcurv_jg12217.t1_Hypnum_curvifolium
Mapoly0088s0022.1.p_Marchantia_polymorpha
Luncru_19532.1_0_Lunularia_cruciata
Mapoly0005s0222.1.p_Marchantia_polymorpha
Marpal_utg000068g0113121_Marchantia_paleacea
Foant_15353_RA_Fontinalis_antipyretica
Pohnut_GWHPBHNB022224_Pohlia_nutans
CepurR40.3G033700.1.p_Ceratodon_purpureus
CepurR40.12G128100.1.p_Ceratodon_purpureus
CepurR40.8G045200.1.p_Ceratodon_purpureus
Pohnut_GWHPBHNB030709_Pohlia_nutans
Pohnut_GWHPBHNB033304_Pohlia_nutans
Hypcurv_jg6401.t1_Hypnum_curvifolium
Entsedu_jg18012.t1_Entodon_seductrix
Calplu_GWHPAMMO023018_Calohypnum_plumiforme
LEA
Pohnut_GWHPBHNB034556_Pohlia_nutans
Pohnut_GWHPBHNB035820_Pohlia_nutans
Pohnut_GWHPBHNB011252_Pohlia_nutans
Pohnut_GWHPBHNB012740_Pohlia_nutans
Ceric.18G048500.1.p_Ceratopteris_richardii
Sacu_v1.1_s0004.g002223_Salvinia_cucullata
Adinel_MCO5604811.1_Adiantum_nelumboides
Adinel_MCO5612499.1_Adiantum_nelumboides
Elagui_XP_010931901.1_Elaeis_guineensis
Elagui_XP_010931925.1_Elaeis_guineensis
Bradi1g25960.1.p_Brachypodium_distachyon
LOC_Os07g34050.1_Oryza_sativa
Sobic.002G322500.1.p_Sorghum_bicolor
Zeama_GRMZM2G309048_P01_Zea_mays
KAF3320415.1_Kobresia_littledalei
KAF3320833.1_Kobresia_littledalei
KAF3320834.1_Kobresia_littledalei
KAF3320835.1_Kobresia_littledalei
evm_27.model.AmTr_v1.0_scaffold00004.282_Amborella_trichopoda
evm_27.model.AmTr_v1.0_scaffold00004.286_Amborella_trichopoda
Solyc02g092400.1.1_Solanum_lycopersicum
Cinka_RWR88431.1_Cinnamomum_kanehirae
Cinka_RWR93426.1_Cinnamomum_kanehirae
Cinka_RWR93427.1_Cinnamomum_kanehirae
Lotjap_LjSGA_011183.1_Lotus_japonicus
Medtr5g005950.1_Medicago_truncatula
Carpa_evm.model.supercontig_10.119_Carica_papaya
MoBGI003658g0030.1_Moringa_oleifera
Medtr4g046830.1_Medicago_truncatula
Medtr4g046787.1_Medicago_truncatula
Medtr4g046803.1_Medicago_truncatula
Lotjap_LjT45D02.140.r2.a_Lotus_japonicus
Lotjap_LjSGA_134299.1_Lotus_japonicus
Medtr2g088610.1_Medicago_truncatula
Medtr2g088680.1_Medicago_truncatula
0.3

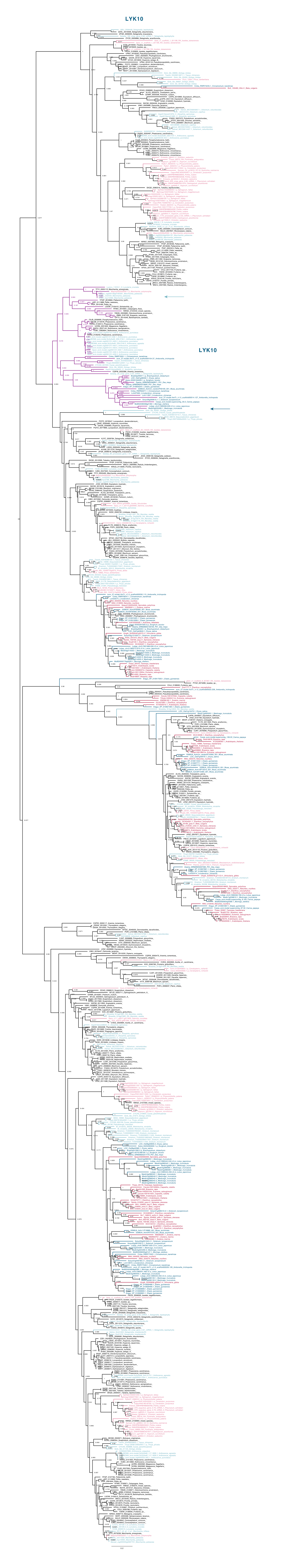
LYK10
LYK10

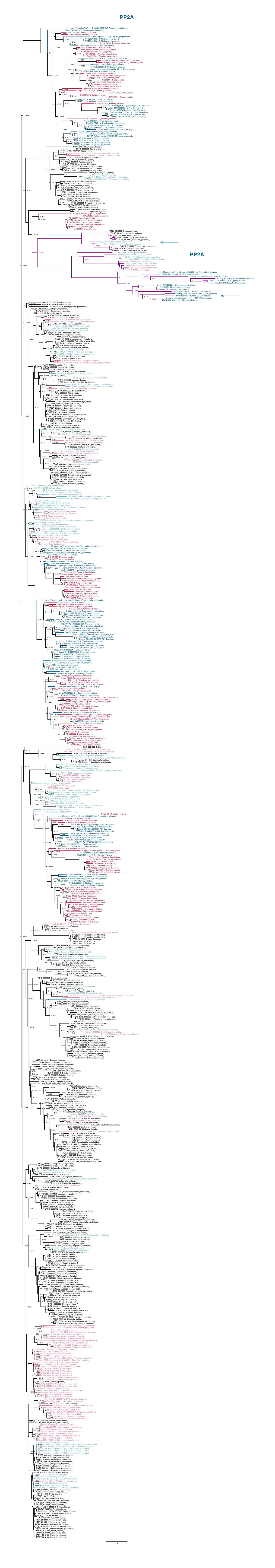
PP2A
PP2A

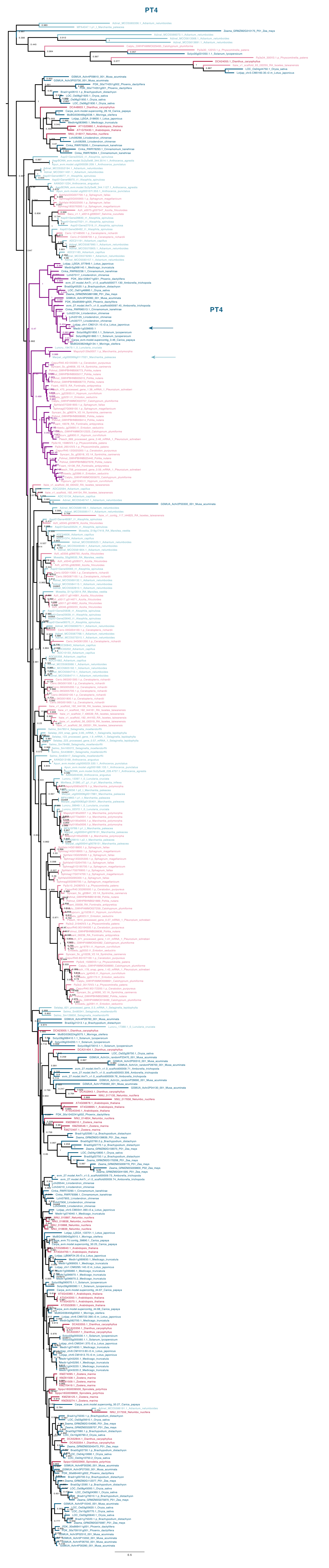
PT4
PT4
0.5

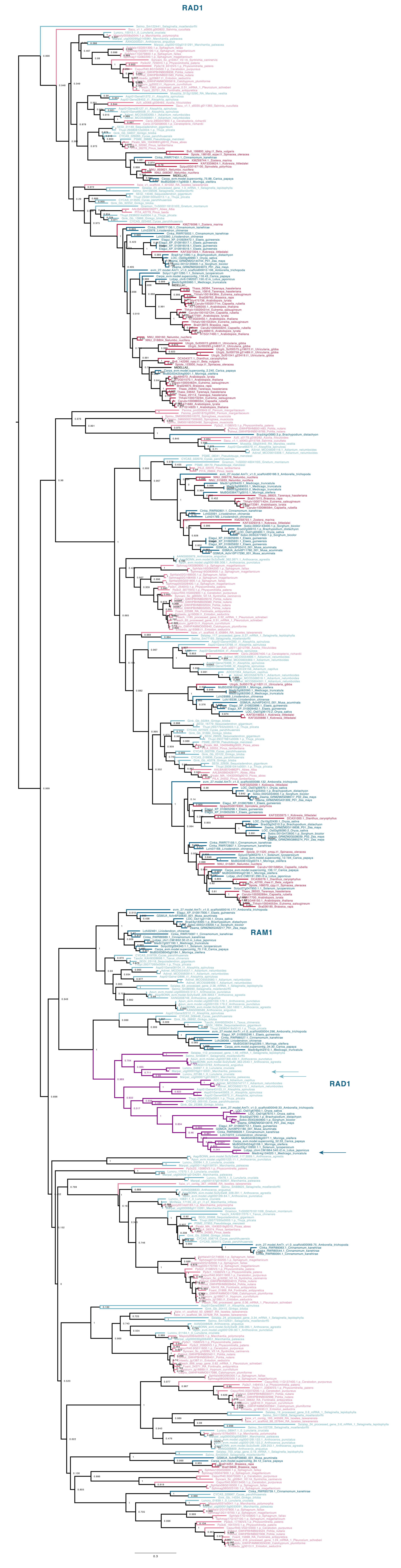
RAD1
RAM1
RAD1
0.3

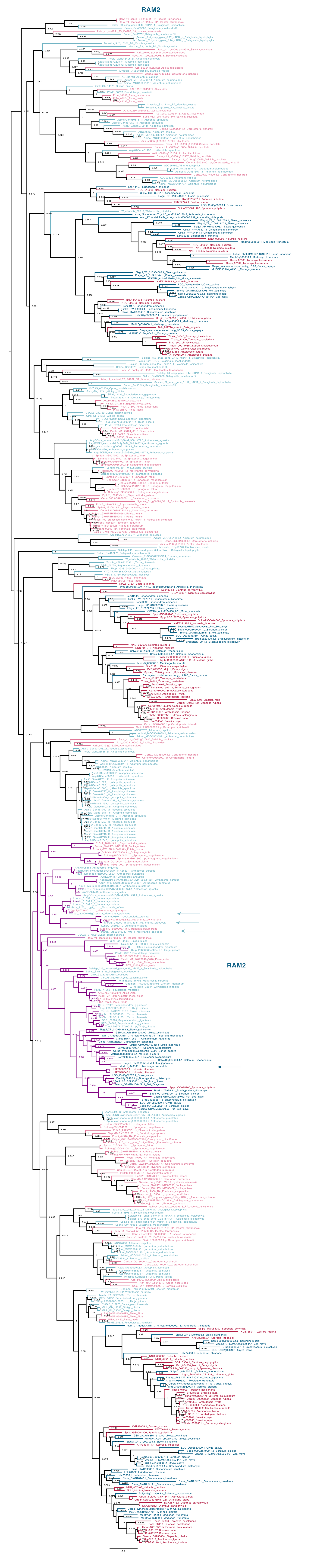
RAM2
RAM2

RFC

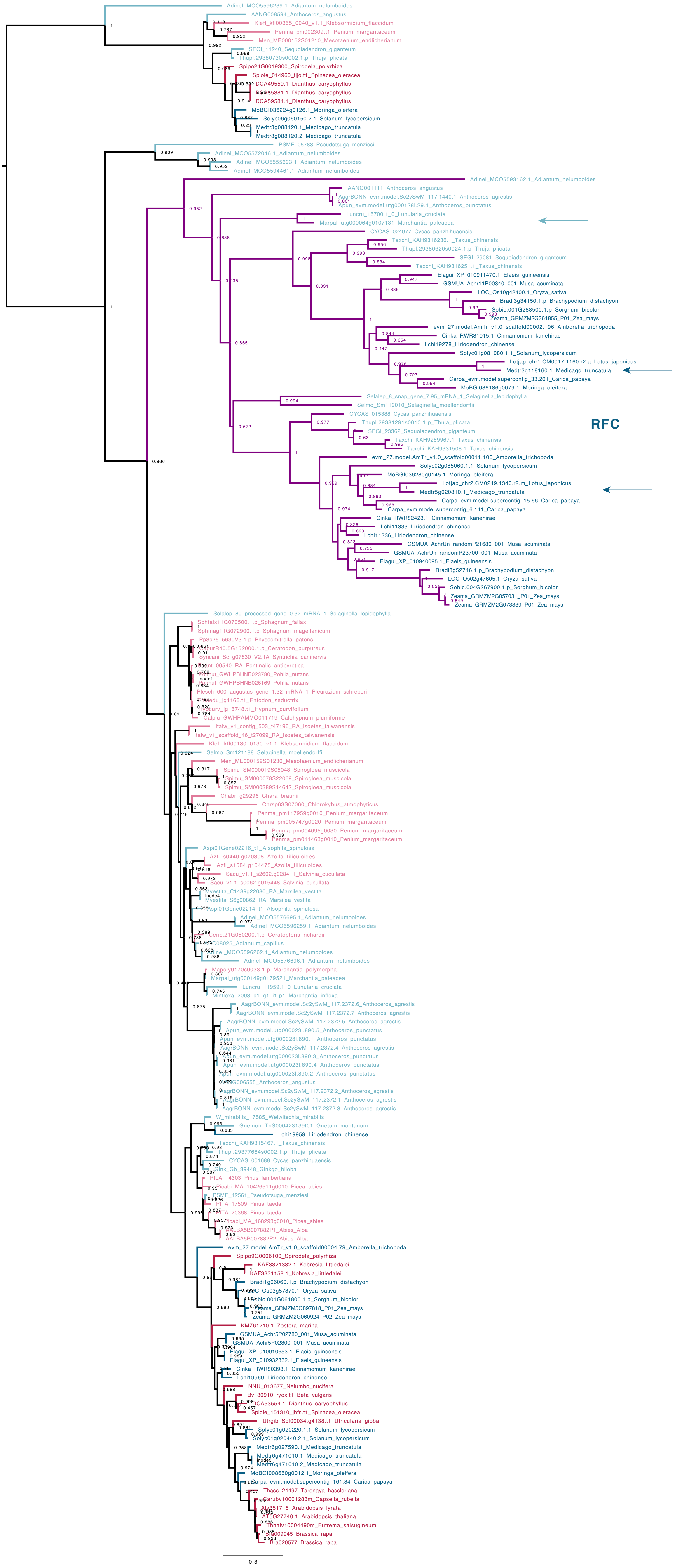
Adinel_MCO5596239.1_Adiantum_nelumboides
AANG008594_Anthoceros_angustus
Klefl_kfl00355_0040_v1.1_Klebsormidium_flaccidum
Penma_pm002309.t1_Penium_margaritaceum
Men_ME000152S01210_Mesotaenium_endlicherianum
SEGI_11240_Sequoiadendron_giganteum
Thupl.29380730s0002.1.p_Thuja_plicata
Spipo24G0019300_Spirodela_polyrhiza
Spiole_014960_fjjo.t1_Spinacea_oleracea
DCA49559.1_Dianthus_caryophyllus
DCA55381.1_Dianthus_caryophyllus
DCA59584.1_Dianthus_caryophyllus
MoBGI036224g0126.1_Moringa_oleifera
Solyc06g060150.2.1_Solanum_lycopersicum
Medtr3g088120.1_Medicago_truncatula
Medtr3g088120.2_Medicago_truncatula
PSME_05783_Pseudotsuga_menziesii
Adinel_MCO5572046.1_Adiantum_nelumboides
Adinel_MCO5555693.1_Adiantum_nelumboides
Adinel_MCO5594461.1_Adiantum_nelumboides
Adinel_MCO5593162.1_Adiantum_nelumboides
AANG001111_Anthoceros_angustus
AagrBONN_evm.model.Sc2ySwM_117.1440.1_Anthoceros_agrestis
Apun_evm.model.utg000128l.29.1_Anthoceros_punctatus
Luncru_15700.1_0_Lunularia_cruciata
Marpal_utg000064g0107131_Marchantia_paleacea
CYCAS_024977_Cycas_panzhihuaensis
Taxchi_KAH9316236.1_Taxus_chinensis
Thupl.29380620s0024.1.p_Thuja_plicata
SEGI_29081_Sequoiadendron_giganteum
Taxchi_KAH9316251.1_Taxus_chinensis
Elagui_XP_010911470.1_Elaeis_guineensis
GSMUA_Achr11P00340_001_Musa_acuminata
LOC_Os10g42400.1_Oryza_sativa
Bradi3g34150.1.p_Brachypodium_distachyon
Sobic.001G288500.1.p_Sorghum_bicolor
Zeama_GRMZM2G361855_P01_Zea_mays
evm_27.model.AmTr_v1.0_scaffold00002.196_Amborella_trichopoda
Cinka_RWR81015.1_Cinnamomum_kanehirae
Lchi19278_Liriodendron_chinense
Solyc01g081080.1.1_Solanum_lycopersicum
Lotjap_chr1.CM0017.1160.r2.a_Lotus_japonicus
Medtr3g118160.1_Medicago_truncatula
Carpa_evm.model.supercontig_33.201_Carica_papaya
MoBGI036186g0079.1_Moringa_oleifera
Selalep_8_snap_gene_7.95_mRNA_1_Selaginella_lepidophylla
Selmo_Sm119010_Selaginella_moellendorffii
CYCAS_015388_Cycas_panzhihuaensis
Thupl.29381291s0010.1.p_Thuja_plicata
SEGI_23362_Sequoiadendron_giganteum
Taxchi_KAH9289967.1_Taxus_chinensis
Taxchi_KAH9331508.1_Taxus_chinensis
RFC
evm_27.model.AmTr_v1.0_scaffold00011.106_Amborella_trichopoda
Solyc02g085060.1.1_Solanum_lycopersicum
MoBGI036280g0145.1_Moringa_oleifera
Lotjap_chr2.CM0249.1340.r2.m_Lotus_japonicus
Medtr5g020810.1_Medicago_truncatula
Carpa_evm.model.supercontig_15.66_Carica_papaya
Carpa_evm.model.supercontig_6.141_Carica_papaya
Cinka_RWR82423.1_Cinnamomum_kanehirae
Lchi11333_Liriodendron_chinense
Lchi11336_Liriodendron_chinense
GSMUA_AchrUn_randomP21680_001_Musa_acuminata
GSMUA_AchrUn_randomP23700_001_Musa_acuminata
Elagui_XP_010940095.1_Elaeis_guineensis
Bradi3g52746.1.p_Brachypodium_distachyon
LOC_Os02g47605.1_Oryza_sativa
Sobic.004G267900.1.p_Sorghum_bicolor
Zeama_GRMZM2G057031_P01_Zea_mays
Zeama_GRMZM2G073339_P01_Zea_mays
Selalep_80_processed_gene_0.32_mRNA_1_Selaginella_lepidophylla
Sphfalx11G070500.1.p_Sphagnum_fallax
Sphmag11G072900.1.p_Sphagnum_magellanicum
Pp3c25_5630V3.1.p_Physcomitrella_patens
CepurR40.5G152000.1.p_Ceratodon_purpureus
Syncani_Sc_g07830_V2.1A_Syntrichia_caninervis
Fontant_00540_RA_Fontinalis_antipyretica
Pohnut_GWHPBHNB023780_Pohlia_nutans
Pohnut_GWHPBHNB026169_Pohlia_nutans
Plesch_600_augustus_gene_1.32_mRNA_1_Pleurozium_schreberi
Entsedu_jg1166.t1_Entodon_seductrix
Hypcurv_jg18748.t1_Hypnum_curvifolium
Calplu_GWHPAMMO011719_Calohypnum_plumiforme
Itaiw_v1_contig_503_t47196_RA_Isoetes_taiwanensis
Itaiw_v1_scaffold_46_t27099_RA_Isoetes_taiwanensis
Klefl_kfl00130_0130_v1.1_Klebsormidium_flaccidum
Selmo_Sm121188_Selaginella_moellendorffii
Men_ME000152S01230_Mesotaenium_endlicherianum
Spimu_SM000019S05048_Spirogloea_muscicola
Spimu_SM000078S22069_Spirogloea_muscicola
Spimu_SM000389S14642_Spirogloea_muscicola
Chabr_g29296_Chara_braunii
Chrsp63S07060_Chlorokybus_atmophyticus
Penma_pm117959g0010_Penium_margaritaceum
Penma_pm005747g0020_Penium_margaritaceum
Penma_pm004095g0030_Penium_margaritaceum
Penma_pm011463g0010_Penium_margaritaceum
Aspi01Gene02216_t1_Alsophila_spinulosa
Azfi_s0440.g070308_Azolla_filiculoides
Azfi_s1584.g104475_Azolla_filiculoides
Sacu_v1.1_s2602.g028411_Salvinia_cucullata
Sacu_v1.1_s0062.g015448_Salvinia_cucullata
Mvestita_C1489g22080_RA_Marsilea_vestita
Mvestita_S6g00862_RA_Marsilea_vestita
Aspi01Gene02214_t1_Alsophila_spinulosa
Adinel_MCO5576695.1_Adiantum_nelumboides
Adinel_MCO5596259.1_Adiantum_nelumboides
Ceric.21G050200.1.p_Ceratopteris_richardii
AC08025_Adiantum_capillus
Adinel_MCO5596262.1_Adiantum_nelumboides
Adinel_MCO5576696.1_Adiantum_nelumboides
Mapoly0170s0033.1.p_Marchantia_polymorpha
Marpal_utg000149g0179521_Marchantia_paleacea
Luncru_11959.1_0_Lunularia_cruciata
Minflexa_2008_c1_g1_i1.p1_Marchantia_inflexa
AagrBONN_evm.model.Sc2ySwM_117.2372.6_Anthoceros_agrestis
AagrBONN_evm.model.Sc2ySwM_117.2372.7_Anthoceros_agrestis
AagrBONN_evm.model.Sc2ySwM_117.2372.5_Anthoceros_agrestis
Apun_evm.model.utg000023l.890.5_Anthoceros_punctatus
Apun_evm.model.utg000023l.890.1_Anthoceros_punctatus
AagrBONN_evm.model.Sc2ySwM_117.2372.4_Anthoceros_agrestis
Apun_evm.model.utg000023l.890.3_Anthoceros_punctatus
Apun_evm.model.utg000023l.890.4_Anthoceros_punctatus
Apun_evm.model.utg000023l.890.2_Anthoceros_punctatus
AANG006555_Anthoceros_angustus
AagrBONN_evm.model.Sc2ySwM_117.2372.2_Anthoceros_agrestis
AagrBONN_evm.model.Sc2ySwM_117.2372.1_Anthoceros_agrestis
AagrBONN_evm.model.Sc2ySwM_117.2372.3_Anthoceros_agrestis
W_mirabilis_17585_Welwitschia_mirabilis
Gnemon_TnS000423139t01_Gnetum_montanum
Lchi19959_Liriodendron_chinense
Taxchi_KAH9315467.1_Taxus_chinensis
Thupl.29377664s0002.1.p_Thuja_plicata
CYCAS_001688_Cycas_panzhihuaensis
Gink_Gb_39448_Ginkgo_biloba
PILA_14303_Pinus_lambertiana
Picabi_MA_10426511g0010_Picea_abies
PSME_42561_Pseudotsuga_menziesii
PITA_17509_Pinus_taeda
PITA_20368_Pinus_taeda
Picabi_MA_168293g0010_Picea_abies
AALBA5B007882P1_Abies_Alba
AALBA5B007882P2_Abies_Alba
evm_27.model.AmTr_v1.0_scaffold00004.79_Amborella_trichopoda
Spipo9G0006100_Spirodela_polyrhiza
KAF3321382.1_Kobresia_littledalei
KAF3331158.1_Kobresia_littledalei
Bradi1g06060.1.p_Brachypodium_distachyon
LOC_Os03g57870.1_Oryza_sativa
Sobic.001G061800.1.p_Sorghum_bicolor
Zeama_GRMZM5G897818_P01_Zea_mays
Zeama_GRMZM2G060924_P02_Zea_mays
KMZ61210.1_Zostera_marina
GSMUA_Achr5P02780_001_Musa_acuminata
GSMUA_Achr5P02800_001_Musa_acuminata
Elagui_XP_010910653.1_Elaeis_guineensis
Elagui_XP_010932332.1_Elaeis_guineensis
Cinka_RWR80393.1_Cinnamomum_kanehirae
Lchi19960_Liriodendron_chinense
NNU_013677_Nelumbo_nucifera
Bv_30910_ryox.t1_Beta_vulgaris
DCA53554.1_Dianthus_caryophyllus
Spiole_151310_jhfs.t1_Spinacea_oleracea
Utrgib_Scf00034.g4138.t1_Utricularia_gibba
Solyc01g020220.1.1_Solanum_lycopersicum
Solyc01g020440.2.1_Solanum_lycopersicum
Medtr6g027590.1_Medicago_truncatula
Medtr6g471010.1_Medicago_truncatula
Medtr6g471010.2_Medicago_truncatula
MoBGI008650g0012.1_Moringa_oleifera
Carpa_evm.model.supercontig_161.34_Carica_papaya
Thass_24497_Tarenaya_hassleriana
Carubv10001283m_Capsella_rubella
AL351718_Arabidopsis_lyrata
AT5G27740.1_Arabidopsis_thaliana
Thhalv10004490m_Eutrema_salsugineum
Bra009945_Brassica_rapa
Bra020577_Brassica_rapa
0.3

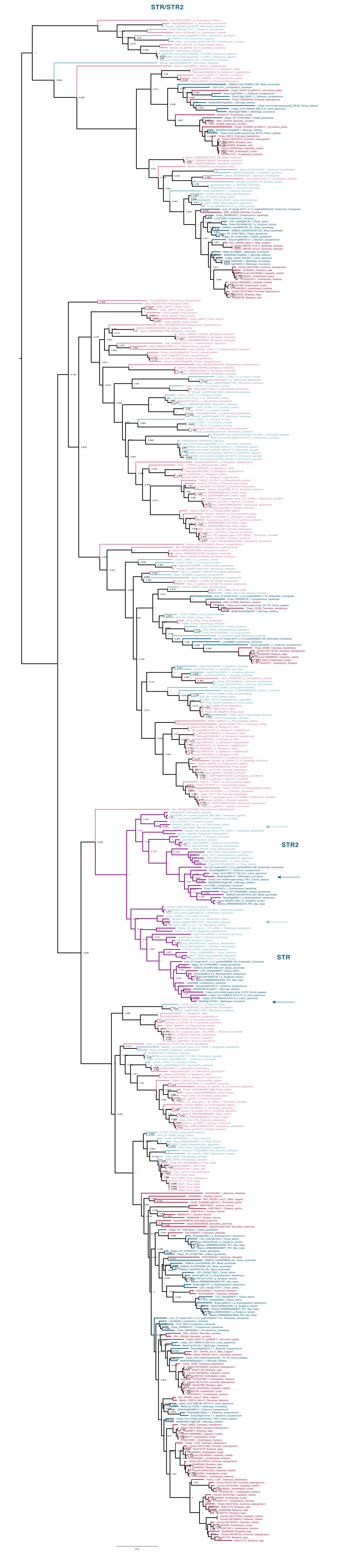

STR/STR2
STR2
STR
0.4

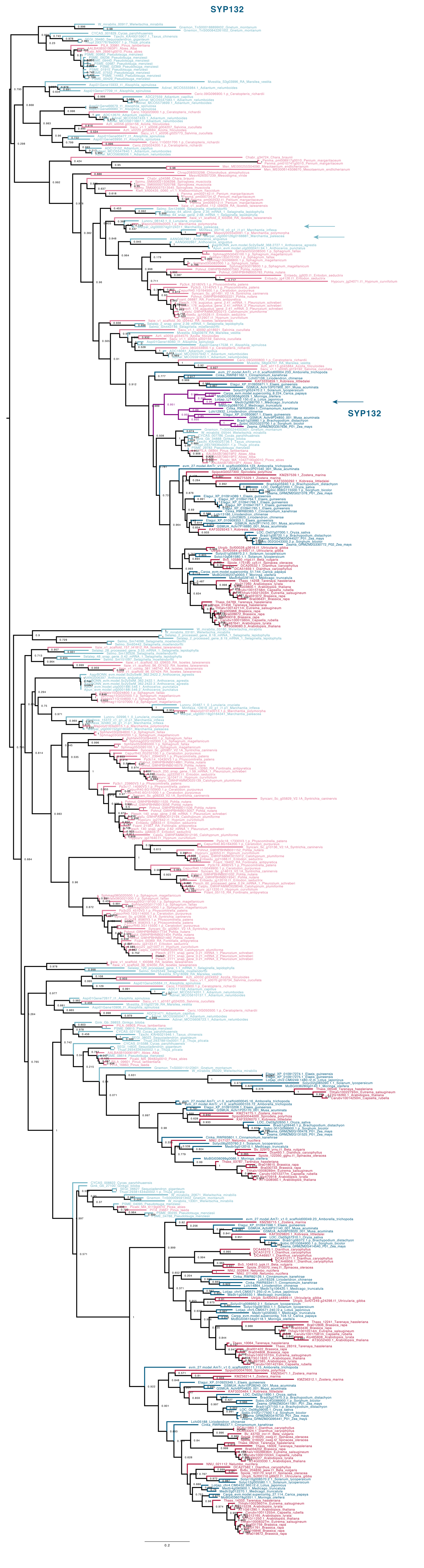
SYP132
SYP132
0.2

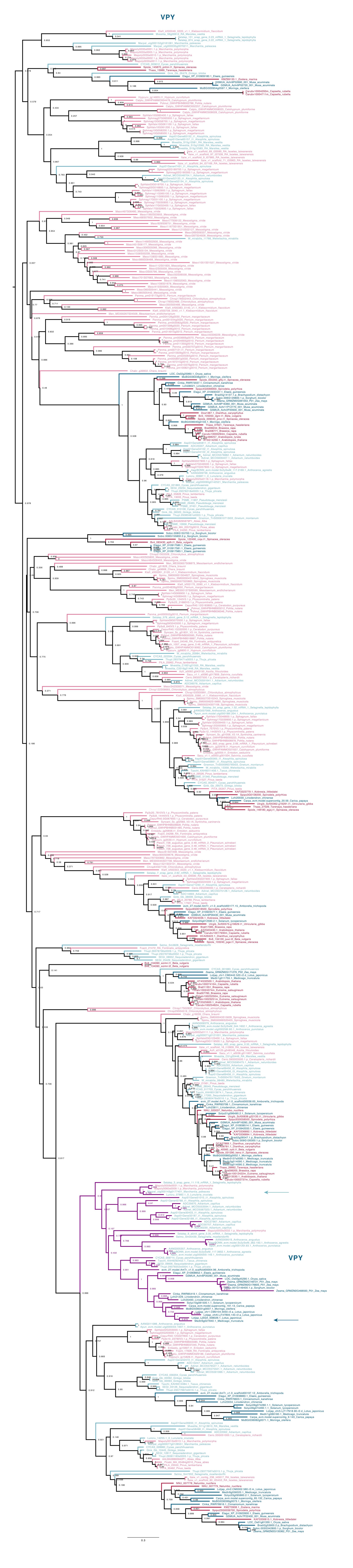
VPY
VPY
0.3

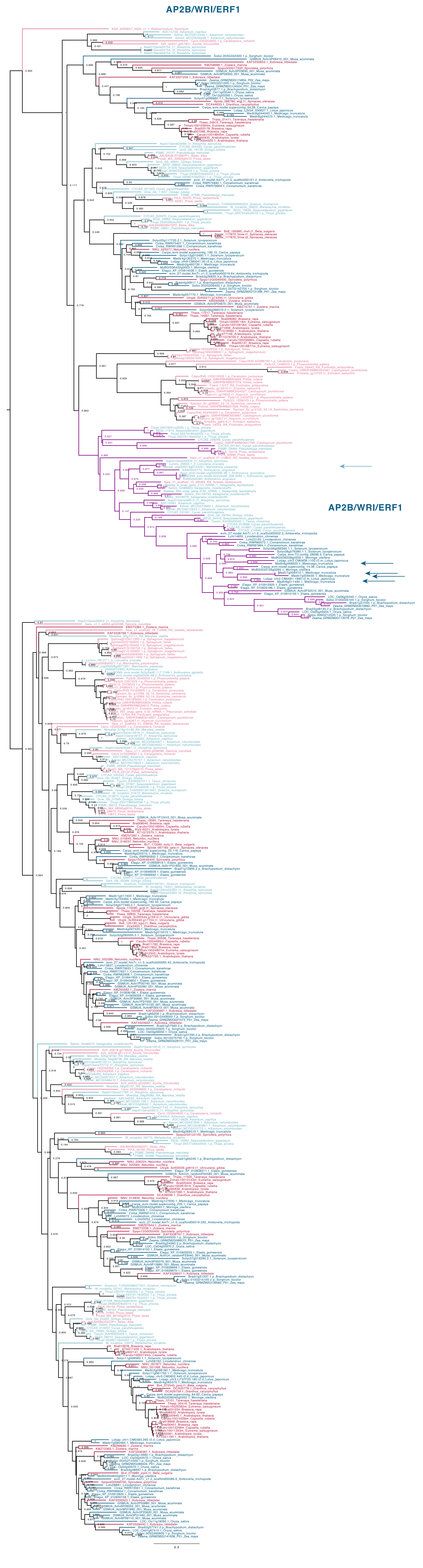
AP2B/WRI/ERF1
AP2B/WRI/ERF1
0.3

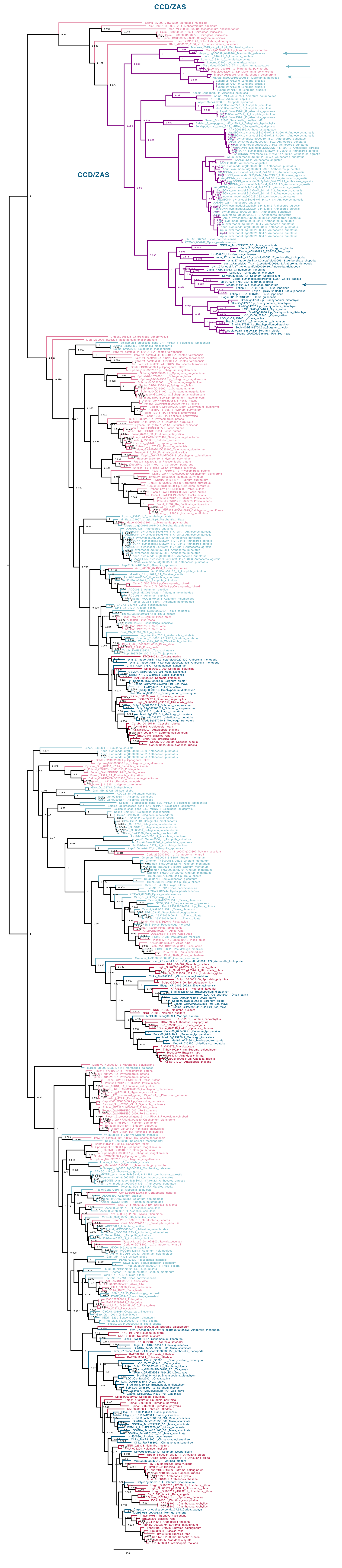
CCD/ZAS
CCD/ZAS
0.3
